# Association of fine particulate matter and its constituents with hypertension: the modifying effect of dietary patterns

**DOI:** 10.1186/s12940-023-01000-y

**Published:** 2023-08-09

**Authors:** Kun Huang, Dongmei Yu, Hongyun Fang, Lahong Ju, Wei Piao, Qiya Guo, Xiaoli Xu, Xiaoqi Wei, Yuxiang Yang, Liyun Zhao

**Affiliations:** 1 National Institute of Nutrition and Health, Chinese Center for Disease Control and Prevention, 27 Nanwei Road, Xicheng District, Beijing, 100050 China; 2 NHC Key Laboratory of Trace Element Nutrition, National Institute for Nutrition and Health, Chinese Center for Disease Control and Prevention, Beijing, 100050 China

**Keywords:** Hypertension, Stage 1 hypertension, Air pollution, Fine particulate matter, Chemical constituents, Diet

## Abstract

**Background:**

Studies have shown that nutritional supplements could reduce the adverse effects induced by air pollution. However, whether dietary patterns can modify the association of long-term exposure to fine particulate matter (PM_2.5_) and its constituents with hypertension defined by the 2017 ACC/AHA guideline has not been evaluated.

**Methods:**

We included 47,501 Chinese adults from a nationwide cross-sectional study. PM_2.5_ and five constituents were estimated by satellite-based random forest models. Dietary approaches to stop hypertension (DASH) and alternative Mediterranean diet (AMED) scores were calculated for each participant. Interactions between dietary patterns and air pollution were examined by adding a multiplicative interaction term to logistic models.

**Results:**

Long-term exposure to PM_2.5_ and its constituents was associated with an increased risk of hypertension and stage 1–2 hypertension. The DASH and AMED scores significantly modified these associations, as individuals with higher scores had a significantly lower risk of air pollution-related hypertension and stage 1–2 hypertension (*P*-interaction < 0.05), except for interaction between PM_2.5_, sulfate, nitrate, ammonium, and AMED score on stage 1 hypertension. For each IQR increase in PM_2.5_, participants with the lowest DASH and AMED quintiles had hypertension risk with ORs (95%CI) of 1.20 (1.10, 1.30) and 1.19 (1.09, 1.29), whereas those with the highest DASH and AMED quintiles had lower risks with 0.98 (0.91, 1.05) and 1.04 (0.97, 1.11). The stratified analysis found modification effect was more prominent in the < 65 years age group. Consuming more fresh vegetables, fruits, whole grains, and dairy would reduce the risk of hypertension caused by PM_2.5_ and its constituents.

**Conclusions:**

Dietary patterns rich in antioxidants can reduce long-term exposure to PM_2.5_ and its constituents-induced hypertension defined by the 2017 ACC/AHA guideline, especially in young and middle-aged individuals. Compared to the Mediterranean diet, the DASH diet offers superior dietary guidance to prevent stage 1 hypertension caused by air pollution.

**Supplementary Information:**

The online version contains supplementary material available at 10.1186/s12940-023-01000-y.

## Introduction

Hypertension (HTN) has been the major preventable risk factor for death and disease burden, responsible for 10.8 million deaths and 237 million disability-adjusted life-years globally in 2019 [1]. In 2010, 1.39 billion adults worldwide suffered from HTN, and the prevalence rate was as high as 31.1% [2]. Due to the population aging and adverse lifestyle, this prevalence is still increasing, especially in low- and middle-income countries (LMICs) [3–5]. In 2018, more than a quarter of Chinese adults had HTN, with high rates of disability and mortality from related complications [6]. Hence, there is an urgent need to control HTN to reduce the related disease burden and mortality.

Ambient fine particulate matter (PM_2.5_) is one of the major air pollutants, ranking fifth among global risk factors for death in 2015 [7]. Previous epidemiological research has shown that long-term exposure to PM_2.5_ was associated with an increased risk of HTN and elevated blood pressure (BP) levels [8–10]. Numerous biological pathways may be involved in the negative impact of ambient PM_2.5_ on BP [11], including oxidative stress and inflammatory response [12]. Ambient PM_2.5_ is a complicated mixture, and its effects on BP depend largely on chemical composition, whereas the components of PM_2.5_ differ depending on the place and the season [13]. To clarify the critical components of PM_2.5_ that increase the risk of HTN is of great significance to reveal the biological mechanism. In China, previous studies on the relationship between PM_2.5_ components and BP are still relatively few and the findings were inconsistent [14–16].

It is well known that diet is also a significant contributor to HTN, and the intake of certain foods and nutrients, such as fruits, vegetables, sodium, and potassium, can affect BP levels [17, 18]. Recent intervention trials have suggested that antioxidants and anti-inflammatory substances can alleviate the negative health consequences of air pollution by lowering oxidative stress and inflammation [19, 20]. Similar mechanisms provide biological plausibility for dietary interventions to reduce HTN risk caused by air pollution. However, recent epidemiology studies have focused on the interaction of a single nutrient or food with air pollution on HTN [21, 22]. The confounding effect caused by food interactions during eating cannot be explained by studying separate food or nutrient. Therefore, it is more reasonable to study the interaction between dietary patterns and the adverse effects of air pollution, and its results are more easily translated into public health guidelines or policies.

There are few studies on the modifying effect of dietary patterns on the relationship between air pollution and HTN, and cohort studies conducted in the United States, Spain and China have not yielded consistent results [23–25]. Additionally, the updated guideline released by the American College of Cardiology (ACC) and the American Heart Association (AHA) in 2017 promoted the stricter definition of HTN [26]. Emerging evidence showed an increased risk of cardiovascular disease (CVD) in stage 1 HTN defined by the 2017 ACC/AHA guideline, highlighting the importance of focusing on stage 1 HTN [27, 28]. However, whether dietary patterns can modify the relationship between air pollution and stage 1 HTN remains unclear.

In this study, we used a nationally representative sample of Chinese adults to assess the relationships between healthy dietary patterns, long-term exposure to PM_2.5_ and its chemical components, and HTN classification as defined by the 2017 ACC/AHA guideline. Furthermore, we explored whether dietary patterns modified the association between air pollution and HTN classification. This study aims to understand better the potential role of healthy dietary patterns in reducing the risk of air pollution-induced HTN and provide evidence for individual-level dietary interventions in the early stages of HTN.

## Methods

### Study population

The study was based on the China Nutrition and Health Surveillance (CNHS) 2015–2017, a nationally representative cross-sectional investigation. The CNHS was divided into two stages by population; the first was the 18-year-old and above people, followed by 2~17-year-old children and adolescents. In this study, only 18 years and above subjects from the first stage (the survey period was from July 2015 to May 2016) of CNHS were selected, which is described in detail elsewhere [29]. Briefly, the Chinese Center for Disease Control and Prevention (CCDC) administered a nationwide nutrition and health surveillance program at 298 Disease Surveillance Points in 31 mainland Chinese provinces, autonomous regions, or municipalities. To ensure representation at the national level, multi-stage cluster random sampling was used to obtain representative samples of adults aged 18 years and above. Only those who have lived in their current residence for more than six months in a year were eligible to participate in the survey. The survey consisted of face-to-face questionnaires, medical examinations, laboratory tests, and dietary surveys, which trained and qualified investigators carried out. A series of standard questionnaires were designed to gather data on demographic characteristics, socioeconomic characteristics, lifestyle behaviors, diet habits, physical activity, physician-diagnosed diseases, family medical history, etc. The medical examination included measurements of height, weight, waist circumference, heart rate, and blood pressure. Laboratory tests include biochemical markers of urine and blood samples. A dietary survey was carried out on a subset of respondents by weighing method, 24-h recall method, and food frequency questionnaire (FFQ). A total of 80,902 subjects took part in the above survey project.

We excluded participants who were > 2,500 m above sea level, had no available information on residence address, had no available exposure, outcome, or adjusted covariates information, or with HTN previously diagnosed by a physician. In total, 47,501 participants aged 18 ~ 79 years were included this analysis. More details on the screening of participants are presented in Fig. S1.

The project was reviewed by the Ethics Review Committee of the CCDC (No. 201519-B), and all subjects signed an informed consent form before the survey.

### Dietary intake assessment

Habitual diets were evaluated for the participants using quantitative FFQ with 64 items, the reproducibility and validity of which have been validated [30]. For each food item, participants were asked to describe the frequency of consumption (times/day, times/week, times/month, and times/year) and the amount (average grams/meal) over the past 12 months. A separate questionnaire was used to ask participants about consumption information on their household condiments and cooking oil in the past month. The individual consumption of condiments and cooking oils was calculated based on the average consumption of a household and the size of the household population. According to the China Food Composition Tables (Standard Edition) [31], daily energy and nutrient intake were calculated for participants.

To capture the dietary characteristics of participants, we assigned each participant a score according to their compliance with two priori dietary patterns associated with cardiovascular health, including the Dietary approaches to stop hypertention (DASH) and the Mediterranean diet. We utilized a modified DASH score to represent diet compliance [32], which replaced skim and low-fat dairy products with all dairy products, as intake of them was very low in this study. Besides, sugar-sweetened beverages were excluded from the dietary components due to their extremely low consumption. As the consumption rate and consumption of nuts is relatively low in the Chinese population, we have combined nuts with legumes into one category. The DASH score includes seven parts: fresh vegetables, fruits, dairy, whole grains, legumes and nuts, red and processed meat, and sodium. The alternative Mediterranean diet (AMED) score was used by us to measure adherence to the Mediterranean diet, which is an adapted version of the traditional Mediterranean for non-Greeks [33, 34]. The AMED score consists of 8 components, including fresh vegetables, fruits, legumes and nuts, fish, ratio of monounsaturated fat acid (MUFA) to saturated fat acid (SFA), whole grains, red and processed meat, and alcohol. According to the sex-specific intake ranking of each dietary component (except for alcohol), we categorized them into quintiles and scored each participant from 1 to 5 score [35, 36]. Fresh vegetables, fruits, dairy, whole grains, legumes and nuts, fish, and MUFA to SFA ratio were assigned points in ascending order of intake. In contrast, red and processed meat and sodium were given points in descending order. Alcohol was scored in five groups according to the appropriate level of intake. Finally, all dietary component scores were added up to obtain corresponding total DASH and AMED scores. More details about the dietary intake assessment are provided in Table S1 and Table S2.

### Exposure assessment

The daily 10 km gridded PM_2.5_ and its constituent concentrations were downloaded from the Tracking Air Pollution in China (TAP) platform (http://tapdata.org.cn/). More details about PM_2.5_ and its chemical composition prediction model can be found in previous publications [37–39]. In short, to create a two-stage machine learning model for PM_2.5_ prediction, the TAP combined observational data on PM_2.5_ with satellite aerosol optical depth retrievals, online Community Multiscale Air Quality (CMAQ) simulations, meteorological reanalysis information, land use, elevation, and population distribution data. The first-stage model uses the synthetic minority oversampling technique to resample the model training data and forecast high pollution episodes. Then the model trained with the resampled data predicts high pollution events. To increase prediction accuracy, the second-stage model predicts the residuals between CMAQ simulations and PM_2.5_ observations using random forests and substitutes PM_2.5_ measurements with residuals. The gaps in satellite retrievals were filled by a decision-tree-based modeling technique. The PM_2.5_ predictions from the two-stage model showed that the out-of-bag R^2^ ranged between 0.80 and 0.88, which indicated this model was quite robust [37].

The PM_2.5_ constituent prediction model was developed based on 10 km gridded PM_2.5_ concentrations. The model extracted PM_2.5_ component information from CMAQ simulations under the constraint of PM_2.5_ total mass. The TAP first improved the wind-blown dust module and then trained the model to correct the simulated PM_2.5_ components ratios based on the ground-based measurements and extreme gradient boosting algorithm. Finally, the PM_2.5_ constituent concentrations were calculated based on the PM_2.5_ species conversion factors. The PM_2.5_ chemical components in TAP agreed well with available ground-based measurements (monthly correlation coefficients of 0.64 to 0.75 from 2000 to 2020 and daily correlation coefficients of 0.67 to 0.80 from 2013 to 2020) [39].

Based on the geocoded residential address, we estimated the three-year average concentrations of PM_2.5_ and its five chemical constituents for each participant before BP measurement (16 July 2015 to 26 May 2016) as surrogates of PM_2.5_ and component exposure. These constituents included sulfate (${\text{SO}}_4^{2-}$), nitrate (${\text{NO}}_3^{-}$), ammonium (${\text{NH}}_4^{+}$), black carbon (BC), and organic matter (OM). The TAP platform estimated annual average ozone concentrations to further adjust for ozone exposure [40].

### Outcome assessment

Blood pressure measurements for each participant were taken by trained medical staff using an electronic sphygmomanometer (OMRON HBP-1300, accuracy: 1mmHg). Participants were asked to avoid smoking, drinking alcohol (coffee or tea), eating, or exercising for half an hour before the blood pressure measurement. Three seated blood pressure measurements were taken in a suitably warm and quiet room, with a one-minute interval between each measurement and a five-minute break before the first measurement. To ensure measurement stability, we took the average of the three readings as the subjects’ BP levels.

According to the 2017 ACC/AHA HTN guideline, we defined the participants as without HTN with systolic blood pressure (SBP) < 130 mmHg and diastolic blood pressure (DBP) < 80 mmHg, HTN (SBP/DBP ≥ 130/80 mmHg), stage 1 HTN (SBP: 130 ~ 139 mmHg or DBP: 80 ~ 89 mmHg), and stage 2 HTN (SBP/DBP ≥ 140/90 mmHg) [26].

### Covariates

Potential confounders were adjusted based on the previous literature about air pollution and BP levels. These covariates included age (strata variable by one year), sex (male and female), education (illiteracy, primary, junior high, senior high, and college and higher education), marital status (unmarried, married or cohabiting, separated or divorced, and widowed), annual household income (< 20,000 CNY, 20,000 ~ 50,000 CNY, > 50,000 CNY, and unknown), smoking status (never and quit smoking or smoking), passive smoking (yes and no), alcohol status (never, < 3 times/month, 1 ~ 4 times/week, and > 5 times/week), physical activity (< 600 MET*min/week, 600 ~ 3,000 MET*min/week, and > 3,000 MET*min/week), outdoor time (quintiles), body mass index (< 18.5 kg/m^2^, 18.5 ~ 23.9 kg/m^2^, 24.0 ~ 27.9 kg/m^2^, and > 28.0 kg/m^2^), household solid fuel use (yes and no), HTN family history (yes and no), residence (urban and rural), region (eastern, southern, central, northern, northwestern, southwestern, and northeastern), diabetes (yes and no), dyslipidemia (yes and no), CVD (yes and no), ozone (continuous variable), and anti-hypertensive drugs use (yes and no, only those subjects with self-reported hypertension were asked about the use of anti-hypertensive medication). Body mass index (BMI) was calculated by dividing body weight by height squared. Physical activity was assessed based on Chinese guidelines for data processing concerning the International Physical Activity Questionnaire [41]. Diabetes was defined as fasting plasma glucose ≥ 7.0 mmol/L, glycated hemoglobin ≥ 6.5%, or physical-diagnosed diabetes [42]. Dyslipidemia was defined as total cholesterol ≥ 6.2 mmol/L, triglycerides ≥ 2.3 mmol/L, high-density lipoprotein cholesterol < 1.0 mmol/L, low-density lipoprotein cholesterol ≥ 4.1 mmol/L, or physical-diagnosed dyslipidemia [43].

### Statistical analysis

The characteristic distribution of continuous variables was described using means and standard deviations (SD), and categorical variables were described using counts and percentages. The one-way analysis of variance and Chi-square tests were used to test for continuous and categorical variables, respectively. The relationships between PM_2.5_ and its components were evaluated using Spearman correlation. Multivariable logistic regression was used to explore the relationship between PM_2.5_ and its species and HTN, stage 1 HTN, and stage 2 HTN. Fully models were adjusted for age, sex, education, marital status, annual household income, smoking status, passive smoking, alcohol status, physical activity, outdoor time, BMI, household solid fuel use, HTN family history, residence, and region. We performed a multicollinearity diagnostic of the model and reported variance inflation factors, and Table S3 shows that the independent variables included in the model were appropriate (GVIF^(1/(2*DF))^ < 2).

In accordance with the quintile distribution of their total scores, the DASH and AMED scores were separated into five levels. We explored the relationship between dietary patterns and outcomes using multivariable logistic regression adjusted for the same covariates as the full model. By deducting alternately one component at a time from the starting score, we assessed the impact of each dietary component on HTN categories related to the DASH and AMED scores, with additional adjustment for the deducted dietary component [44].

To explore the potential effect modification of dietary patterns, we added a multiplicative interaction term between air pollution and dietary patterns into the model. We tested the statistical significance of the interaction through a likelihood ratio test, including and excluding the interaction term. Additionally, we applied the Johnson-Neyman technique and plot to elucidate and visualize the conditional effect of HTN, stage 1 HTN, and stage 2 HTN according to the change in dietary scores and PM_2.5_ and its constituents [45, 46]. In this case, the DASH and AMED scores were considered as continuous variables.

We performed a stratified analysis (< 65 and ≥ 65 years age group) to estimate the age-specificity of the interaction between air pollution and dietary patterns. In addition, we explored the modification effects of each dietary component by incorporating each component into the model as a component score and further adjusting for other dietary components.

We carried out a series of sensitivity analyses to assess the robustness of the results. First, we used different exposure window concentrations before BP measurement to analyze the association of PM_2.5_ and its chemical components with HTN classification. Second, two-pollutant models adjusted for ozone concentrations were conducted to evaluate the effect of concomitant ozone on the interaction between dietary patterns, PM_2.5,_ and its constituents. Third, we controlled for preexisting conditions (diabetes, hyperlipidemia, stroke, and ischemic heart disease) to reduce the impact of comorbidities. Fourth, the participant previously diagnosed with HTN (n = 10,123) were included to estimate the modifying effect of dietary patterns. In this analysis, the HTN was defined as SBP/DBP ≥ 140/90 mmHg or physician-diagnosed HTN, and we additionally adjusted for anti-hypertensive drug use.

In the above analysis, the effect estimates were presented as odds ratios (ORs) with corresponding 95% confidence intervals (CIs). The risk for the three outcomes of HTN, stage 1 HTN and stage 2 HTN was assessed using participants without HTN as a control group. All statistical analyses were conducted in SAS version 9.4(SAS Institute, Inc., Cary, NC) and R version 4.1.2(Vienna, Austria). *P*-values < 0.05 for two-sided tests were considered statistically significant.

## Results

The characteristics of the participants are shown in Table 1. A total of 47,501 participants aged 18 ~ 79 years were included in the current study. Compared to those without HTN, the participants with HTN were older, more male, more married, had a higher BMI, used more solid fuels, mainly were from rural areas, spent more time outdoors, smokers more, drank more frequently, were less educated and had lower household incomes. In addition, participants with HTN had lower dietary scores and consumed less fruits, dairy, legumes and nuts, sodium, and fish than participants without HTN. Participants with HTN also tended to be more exposed to PM_2.5_ and its components. Table S4 shows the basic characteristics of the participants based on the quintiles of the three-year average PM_2.5_ concentration.

**Table 1:** Characteristics of participants by HTN status

Characteristics	HTN	Without HTN	Stage 1 HTN	Stage 2 HTN	*P* values
	(n = 27,004)	(n = 20,497)	(n = 12,552)	(n = 14,452)	
Age, years, mean (SD)	54.2 (12.7)	45.5 (13.8)	50.8 (12.9)	57.1 (11.7)	< 0.001
SBP, mm Hg, mean (SD)	143.1 (16.2)	116.1 (8.4)	130.8 (6.2)	153.7 (14.7)	< 0.001
DBP, mm Hg, mean (SD)	83.9 (9.6)	70.0 (6.4)	80.2 (6.2)	87.1 (10.8)	< 0.001
**Air pollutants, µg/m** ^**3**^ ** (SD)**					
PM_2.5_	54.5 (23.1)	51.9 (22.7)	54.1 (23.1)	54.9 (23.1)	< 0.001
${\text{SO}}_4^{2-}$	10.0 (3.9)	9.6 (3.9)	10.0 (3.9)	10.1 (3.9)	< 0.001
${\text{NO}}_3^{-}$	11.1 (5.4)	10.3 (5.4)	11.0 (5.4)	11.3 (5.4)	< 0.001
${\text{NH}}_4^{+}$	7.7 (3.3)	7.2 (3.4)	7.6 (3.4)	7.8 (3.4)	< 0.001
BC	2.7 (0.9)	2.6 (0.9)	2.7 (0.9)	2.7 (0.9)	< 0.001
OM	13.3 (5.4)	12.7 (5.3)	13.2 (5.4)	13.4 (5.4)	< 0.001
**Dietary score**					
DASH	20.0 (4.6)	20.6 (4.7)	20.2 (4.6)	19.9 (4.6)	< 0.001
AMED	24.1 (4.3)	24.2 (4.2)	24.2 (4.3)	24.0 (4.3)	0.211
Fruits	2.9 (1.4)	3.1 (1.4)	3.0 (1.4)	2.8 (1.4)	< 0.001
Vegetables	3.1 (1.4)	3.1 (1.4)	3.1 (1.4)	3.1 (1.4)	0.054
Dairy	2.3 (1.7)	2.6 (1.8)	2.3 (1.7)	2.2 (1.7)	< 0.001
Whole grains	2.8 (1.7)	2.7 (1.6)	2.7 (1.6)	2.8 (1.7)	< 0.001
Legumes and nuts	3.0 (1.4)	3.1 (1.4)	3.0 (1.4)	2.9 (1.4)	< 0.001
Red meat	3.1 (1.4)	2.9 (1.4)	3.0 (1.4)	3.2 (1.4)	< 0.001
Sodium	2.9 (1.4)	3.1 (1.4)	3.0 (1.4)	2.9 (1.4)	< 0.001
MUFA: SFA	3.1 (1.4)	2.9 (1.4)	3.0 (1.4)	3.2 (1.4)	< 0.001
Fish	2.9 (1.4)	3.1 (1.4)	3.0 (1.4)	2.8 (1.4)	< 0.001
Alcohol	3.3 (0.8)	3.3 (0.7)	3.3 (0.8)	3.3 (0.9)	< 0.001
**Sex (%)**					< 0.001
Male	14,068 (52.1)	8066 (39.4)	6630 (52.8)	7438 (51.5)	
Female	12,936 (47.9)	12,431 (60.6)	5922 (47.2)	7014 (48.5)	
**Education level (%)**					< 0.001
Illiteracy	7818 (29.0)	4102 (20.0)	3038 (24.2)	4780 (33.1)	
Primary	5807 (21.5)	3786 (18.5)	2586 (20.6)	3221 (22.3)	
Junior high	8428 (31.2)	6882 (33.6)	4240 (33.8)	4188 (29.0)	
Senior high	3417 (12.7)	3189 (15.6)	1761 (14.0)	1656 (11.5)	
College and higher	1534 (5.6)	2538 (12.3)	927 (7.4)	607 (4.1)	
**Marital status (%)**					< 0.001
Married or cohabiting	25,120 (93.0)	18,735 (91.4)	11,691 (93.1)	13,429 (92.9)	
Widowed	939 (3.5)	351 (1.7)	291 (2.3)	648 (4.5)	
Separated or divorced	190 (0.7)	162 (0.8)	87 (0.7)	103 (0.7)	
Never married	755 (2.8)	1249 (6.1)	483 (3.9)	272 (1.9)	
**Annual family income, CYN (%)**					< 0.001
< 20,000	6118 (22.7)	3644 (17.8)	2503 (19.9)	3615 (25.0)	
20,000~49,999	9394 (34.8)	6940 (33.9)	4475 (35.7)	4919 (34.0)	
≥ 50,000	7198 (26.7)	6740 (32.9)	3677 (29.3)	3521 (24.4)	
Unknown	4294 (15.8)	3173 (15.4)	1897 (15.1)	2397 (16.6)	
**BMI, kg/m** ^**2**^ ** (%)**					< 0.001
18.5~23.9	11,495 (42.6)	12,030 (58.7)	5817 (46.3)	5678 (39.3)	
< 18.5	778 (2.9)	1225 (6.0)	393 (3.1)	385 (2.7)	
24.0~27.9	10,285 (38.1)	5852 (28.6)	4625 (36.9)	5660 (39.2)	
≥ 28.0	4446 (16.4)	1390 (6.7)	1717 (13.7)	2729 (18.8)	
**Smoking status (%)**					< 0.001
Never	17,154 (63.5)	14,792 (72.2)	7938 (63.2)	9216 (63.8)	
Smoking or quit smoking	9850 (36.5)	5705 (27.8)	4614 (36.8)	5236 (36.2)	
**Passive smoking (%)**					< 0.001
Yes	17,931 (66.4)	13,930 (68.0)	8560 (68.2)	9371 (64.8)	
No	9073 (33.6)	6567 (32.0)	3992 (31.8)	5081 (35.2)	
**Alcohol consumption (%)**					< 0.001
Never	16,579 (61.4)	13,600 (66.4)	7537 (60.1)	9042 (62.6)	
≤ 3 times/ month	4306 (16.0)	4062 (19.8)	2229 (17.8)	2077 (14.4)	
1–4 times/ week	2623 (9.7)	1591 (7.8)	1333 (10.6)	1290 (8.9)	
≥ 5 times/ week	3496 (13.0)	1244 (6.0)	1453 (11.5)	2043 (14.1)	
**Household solid-fuel use (%)**					< 0.001
Yes	10,637 (39.4)	6926 (33.8)	4583 (36.5)	6054 (41.9)	
No	16,367 (60.6)	13,571 (66.2)	7969 (63.5)	8398 (58.1)	
**Physical activity (%)**					< 0.001
Low	6439 (23.8)	4720 (23.0)	2960 (23.6)	3479 (24.1)	
Moderate	6498 (24.1)	5364 (26.2)	3074 (24.5)	3424 (23.7)	
High	14,067 (52.1)	10,413 (50.8)	6518 (51.9)	7549 (52.2)	
**HTN family history (%)**					0.084
Yes	7794 (28.9)	6065 (29.6)	3670 (29.2)	4124 (28.5)	
No	19,210 (71.1)	14,432 (70.4)	8882 (70.8)	10,328 (71.5)	
**Residence (%)**					< 0.001
Urban	10,498 (38.9)	9064 (44.2)	5090 (40.6)	5408 (37.4)	
Rural	16,506 (61.1)	11,433 (55.8)	7462 (59.4)	9044 (62.6)	
**Region (%)**					< 0.001
North	4366 (16.2)	2496 (12.2)	1916 (15.3)	2450 (17.0)	
Northeast	3060 (11.3)	1954 (9.4)	1423 (11.3)	1637 (11.3)	
East	7305 (27.1)	5403 (26.4)	3498 (27.9)	3807 (26.3)	
Central	3571 (13.2)	2475 (12.1)	1644 (13.1)	1927 (13.3)	
Southwest	3408 (12.6)	3028 (14.8)	1546 (12.3)	1862 (12.9)	
Northwest	2614 (9.7)	2484 (12.1)	1191 (9.5)	1423 (9.9)	
South	2680 (9.9)	2657 (13.0)	1334 (10.6)	1346 (9.3)	
**Outdoor time, mins/day (%)**					< 0.001
Quintile 1 [0,75]	5118 (19.0)	4552 (22.2)	2498 (19.9)	2620 (18.1)	
Quintile 2 [76,142]	5136 (19.0)	4038 (19.7)	2416 (19.3)	2720 (18.8)	
Quintile 3 [143,225]	5493 (20.3)	4090 (20.0)	2488 (19.8)	3005 (20.8)	
Quintile 4 [226,357]	5334 (19.8)	3705 (18.1)	2380 (19.0)	2954 (20.4)	
Quintile 5 [360,750]	5923 (21.9)	4112 (20.0)	2770 (22.0)	3153 (21.9)	

Abbreviations: HTN, hypertension; SD, standard deviation; SBP, systolic blood pressure; DBP, diastolic blood pressure; PM_2.5_, fine particulate matter; ${\text{SO}}_4^{2-}$, sulfate; ${\text{NO}}_3^{-}$, nitrate; ${\text{NH}}_4^{+}$, ammonium; BC, black carbon; OM, organic matter; DASH, dietary approaches to stop hypertension; AMED, alternative Mediterranean diet; MUFA: SFA, the ratio of monounsaturated to saturated fatty acids; BMI, body mass index

Note: Data were expressed as Mean (SD) for continuous variables and counts (%) for categorical variables. *P* values for the test of variance between participants with HTN and those without HTN were calculated

**Fig. 1:**
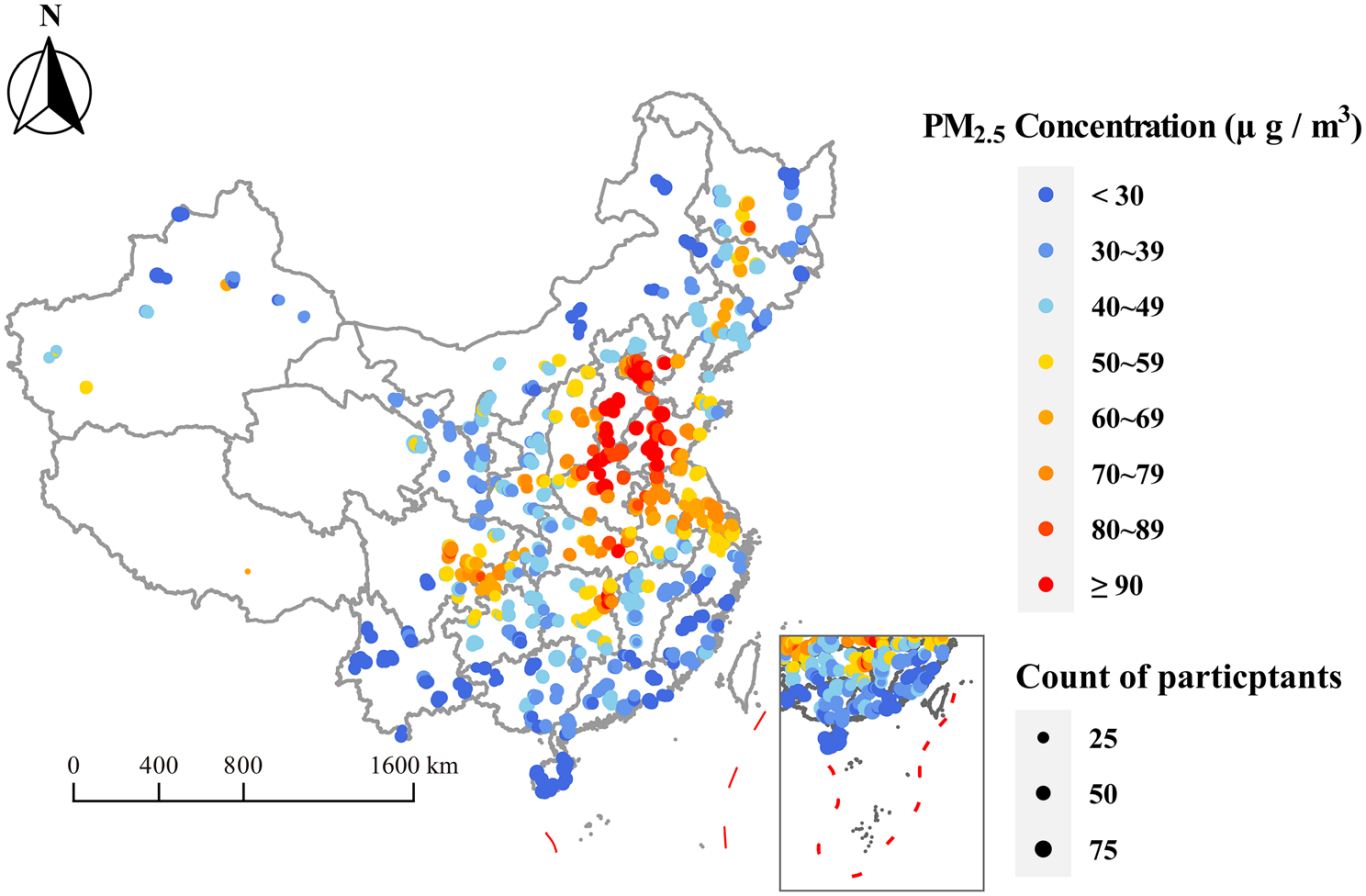
The map of participants’ locations and 3-year average PM_2.5_ concentrations

Figure 1 shows the location of the study sites and the 3-year PM_2.5_ exposure level for 47,501 participants. Table 2 summarizes the distribution of the long-term exposure to PM_2.5_ and its components for the participants before BP measurement. The 3-year mean (SD) PM_2.5_ concentration was 53.4 (23.0) µg/m^3^ with a range of 13.4 to 116.5 µg/m^3^. For the five particulate constituents, OM had the highest average exposure concentration (13.1 µg/m^3^), followed by ${\text{NO}}_3^{-}$ (10.8 µg/m^3^), and the lowest was BC (2.6 µg/m^3^), with an interquartile range (IQR) of 1.3 to 8.6 µg/m^3^. Table 3 shows a high positive correlation between PM_2.5_ and its chemical components, with the Spearman correlation coefficients ranging from 0.86 to 0.99.

**Table 2:** Summary distributions of the three-year average PM_2.5_ and its constituents before BP measurement

Pollutants (µg/m^3^)	Mean	SD	Min	P25	Median	P75	Max	IQR
PM_2.5_	53.4	23.0	13.4	35.6	48.0	70.3	116.5	34.6
${\text{SO}}_4^{2-}$	9.9	3.9	3.2	6.6	9.0	13.0	21.3	6.3
${\text{NO}}_3^{-}$	10.8	5.4	2.0	6.4	9.6	15.0	26.1	8.6
${\text{NH}}_4^{+}$	7.5	3.4	1.8	4.7	6.9	10.1	17.3	5.4
BC	2.6	0.9	1.0	1.9	2.4	3.2	5.7	1.3
OM	13.1	5.3	5.3	9.1	11.7	16.7	32.2	7.6

Abbreviations: PM_2.5_, fine particulate matter; ${\text{SO}}_4^{2-}$, sulfate; ${\text{NO}}_3^{-}$, nitrate; ${\text{NH}}_4^{+}$, ammonium; BC, black carbon; OM, organic matter; SD, standard deviation; IQR, interquartile range.

**Table 3:** Spearman correlations between PM_2.5_ and its chemical constituents

	${\text{SO}}_4^{2-}$	${\text{NO}}_3^{-}$	${\text{NH}}_4^{+}$	BC	OM
PM_2.5_	0.95	0.95	0.93	0.94	0.97
${\text{SO}}_4^{2-}$		0.95	0.96	0.94	0.95
${\text{NO}}_3^{-}$			0.99	0.86	0.91
${\text{NH}}_4^{+}$				0.86	0.90
BC					0.98

Abbreviations: PM_2.5_, fine particulate matter; ${\text{SO}}_4^{2-}$, sulfate; ${\text{NO}}_3^{-}$, nitrate; ${\text{NH}}_4^{+}$, ammonium; BC, black carbon; OM, organic matter.

Table 4 shows significant positive relations between long-term exposures to PM_2.5_ and its constituents and HTN, stage 1 HTN, and stage 2 HTN in crude models. The ORs of HTN classification risk decreased after fully controlling for covariates. For per IQR increase in PM_2.5_, the ORs (95%CIs) of HTN, stage 1 HTN, and stage 2 HTN were 1.08 (1.03, 1.12), 1.06 (1.01, 1.12), and 1.08 (1.03, 1.13), respectively. The five PM_2.5_ components were likewise significantly associated with the risks for HTN, with the largest ORs per IQR increase observed in ${\text{NO}}_3^{-}$ (OR = 1.16, 95%CI: 1.11, 1.20) and ${\text{NH}}_4^{+}$ (OR = 1.16, 95%CI: 1.12, 1.21), followed by ${\text{SO}}_4^{2-}$ (OR = 1.11, 95%CI: 1.07, 1.16), OM (OR = 1.07, 95%CI: 1.03, 1.11), BC (OR = 1.05, 95%CI: 1.02, 1.09). The ORs between PM_2.5_ and its components and the risk of stage 2 HTN were higher than those of stage 1 HTN. For PM_2.5_ constituents associated with risk of HTN categories, ${\text{SO}}_4^{2-}$, ${\text{NO}}_3^{-}$, and ${\text{NH}}_4^{+}$ showed more significant ORs than PM_2.5_ total mass, while OM and BC showed lower ORs than PM_2.5_ total mass.

**Table 4:** Odds ratios and 95% confidence intervals for HTN, stage 1 HTN, and stage 2 HTN associated with an IQR increase in the 3-year average concentration of PM_2.5_ and its constituents

Pollutants	HTN			Stage 1 HTN			Stage 2 HTN	
	Crude model	Fully model		Crude model	Fully model		Crude model	Fully model
PM_2.5_	1.19 (1.16, 1.23)	1.08 (1.03, 1.12)		1.15 (1.12, 1.19)	1.06 (1.01, 1.12)		1.22 (1.18, 1.26)	1.08 (1.03, 1.13)
${\text{SO}}_4^{2-}$	1.20 (1.16, 1.23)	1.11 (1.07, 1.16)		1.16 (1.12, 1.20)	1.09 (1.04, 1.14)		1.23 (1.18, 1.27)	1.12 (1.07, 1.18)
${\text{NO}}_3^{-}$	1.28 (1.24, 1.32)	1.16 (1.11, 1.20)		1.22 (1.18, 1.27)	1.12 (1.07, 1.18)		1.32 (1.27, 1.36)	1.17 (1.12, 1.23)
${\text{NH}}_4^{+}$	1.28 (1.24, 1.31)	1.16 (1.12, 1.21)		1.21 (1.18, 1.27)	1.13 (1.08, 1.18)		1.32 (1.28, 1.37)	1.18 (1.13, 1.24)
BC	1.13 (1.10, 1.16)	1.05 (1.02, 1.09)		1.10 (1.06, 1.13)	1.04 (1.00, 1.08)		1.16 (1.13, 1.20)	1.06 (1.02, 1.11)
OM	1.17 (1.14, 1.20)	1.07 (1.03, 1.11)		1.12 (1.09, 1.16)	1.04 (1.00, 1.09)		1.21 (1.17, 1.24)	1.08 (1.04, 1.13)

Abbreviations: HTN, hypertension; PM_2.5_, fine particulate matter; ${\text{SO}}_4^{2-}$, sulfate; ${\text{NO}}_3^{-}$, nitrate; ${\text{NH}}_4^{+}$, ammonium; BC, black carbon; OM, organic matter; IQR, interquartile range.

Note: Fully models were adjusted for age, sex, education, marital status, annual household income, smoking status, passive smoking, alcohol status, physical activity, outdoor time, BMI, household solid fuel use, HTN family history, residence, and region.

**Fig. 2:**
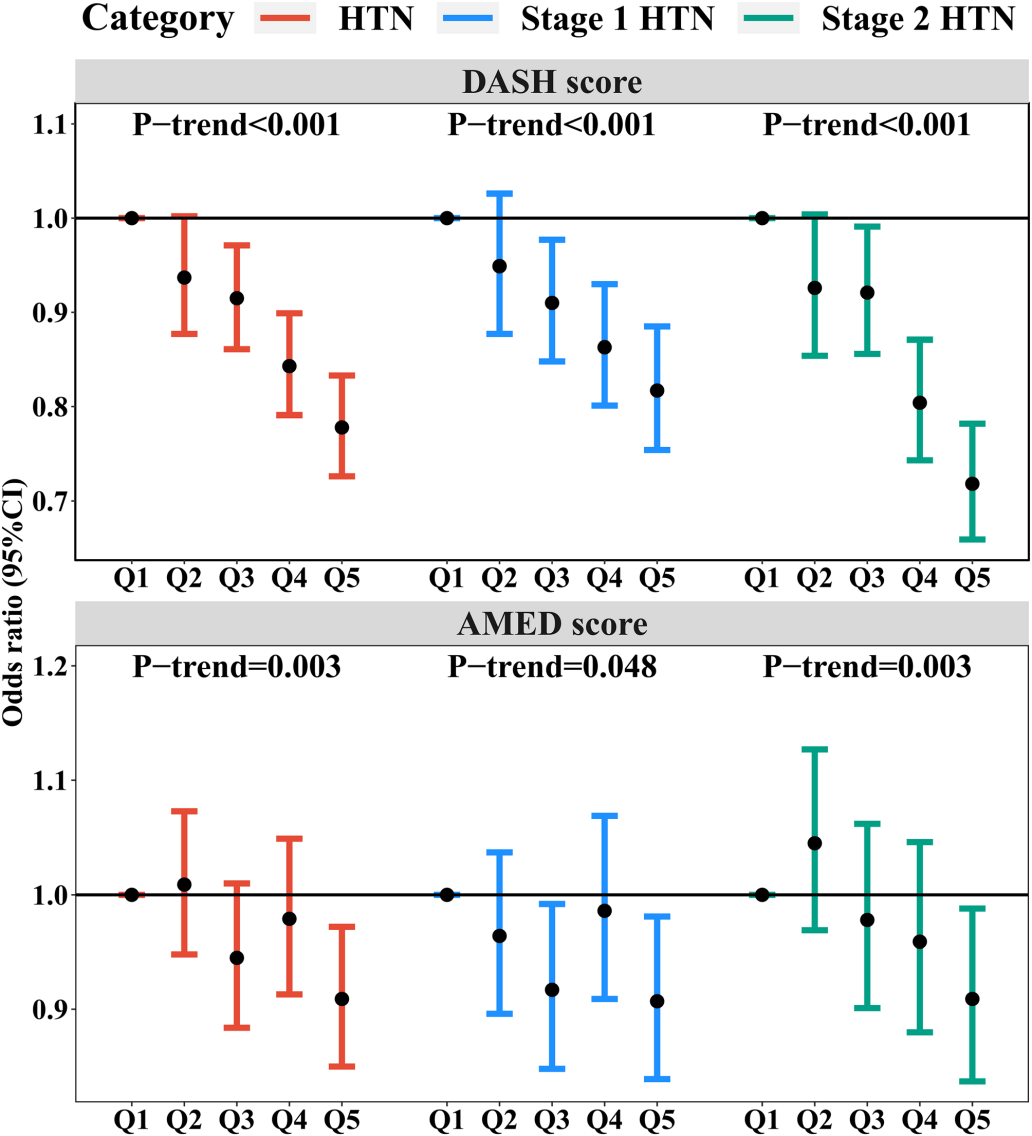
Odds ratios and 95% confidence intervals of risks of HTN, stage 1 HTN, and stage 2 HTN by quintiles of DASH and AMED scores. Note: Fully models were adjusted for age, sex, education, marital status, annual household income, smoking status, passive smoking, alcohol status, physical activity, outdoor time, BMI, household solid fuel use, HTN family history, residence, and region. *P*-trend indicates the *P* values of the trend test. Abbreviations: HTN, hypertension; DASH, dietary approaches to stop hypertension; AMED, althernative Mediterranean diet

Figure 2 (and Table S5) shows the association of DASH and AMED scores with the risk of HTN, stage 1 HTN, and stage 2 HTN. We observed a significant inverse relationship between increased DASH and AMED scores and risk of HTN, stage 1 HTN, and stage 2 HTN (all *P*-trend < 0.05). Compared to the lowest quintile (Q1), the highest quintile (Q5) of the DASH score showed inverse associations with the risk of HTN (OR = 0.78, 95%CI: 0.73, 0.83), stage 1 HTN (OR = 0.82, 95%CI: 0.75, 0.89), and stage 2 HTN (OR = 0.72, 95%CI: 0.66, 0.78). The Q5 of AMED score has a significant protective effect on HTN (OR = 0.91, 95%CI: 0.85, 0.97), stage 1 HTN (OR = 0.91, 95%CI: 0.84, 0.98), and stage 2 HTN (OR = 0.91, 95%CI: 0.84, 0.99), but the effect was lower than that of DASH score. Although DASH and AMED scores have several of the same components, they have different protective effects against HTN. The Q5 of AMED score only reduced about 9% of risks of HTN, stage 1 HTN, and stage 2 HTN than the lowest quintiles of AMED score. A single-component analysis was conducted by alternately subtracting one component at a time from the original score to better understand the discrepant results between DASH and AMED scores. The exclusion of each component of the DASH score can cause a decrease in the protective effect of the DASH score (Table S6). The beneficial effect on HTN risk of the DASH score was reduced the most (69.1%) when dairy was excluded from the DASH score, followed by fruits (33.3%), legumes, and nuts (30.9%). The protective effect on the risk of stage 1 HTN was the top three by dairy (69.7%), legumes and nuts (28.8%), and whole grains (28.8%). For stage 2 HTN risk, Dairy products (68.3%), legumes and nuts (35.6%), and fruits (28.8%) ranked in the top three for their protective effect in the DASH score. However, in a single-component analysis of AMED score (Table S7), the MUFA: SFA and red meat even showed harmful effects on HTN (26.5% and −14.7%), stage 1 HTN (23.1% and 7.7%), and stage 2 HTN (27.9% and 14.0%). Legumes and nuts components included in AMED score contributed most of the proportion to the beneficial effects of AMED with HTN (73.5%), stage 1 HTN (80.8%), and stage 2 HTN (74.4%).

**Fig. 3:**
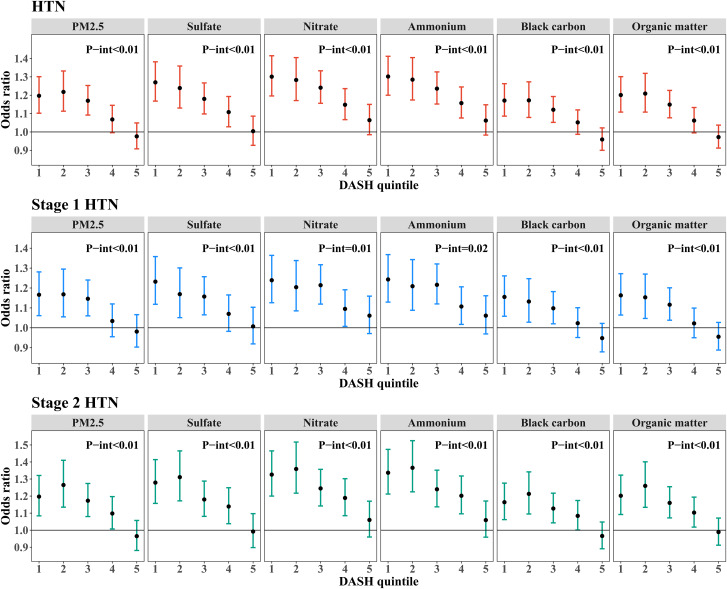
Odds ratios and 95% confidence intervals of HTN, stage 1 HTN, and stage 2 HTN associated with PM_2.5_ and its constituents by quintiles of DASH score. Note: Fully models were adjusted for age, sex, education, marital status, annual household income, smoking status, passive smoking, alcohol status, physical activity, outdoor time, BMI, household solid fuel use, HTN family history, residence, and region. *P*-int indicates the *P* values of the modifying effects of DASH score. Abbreviations: HTN, hypertension; PM_2.5_, fine particulate matter; IQR, interquartile range; DASH, dietary approaches to stop hypertension

**Fig. 4:**
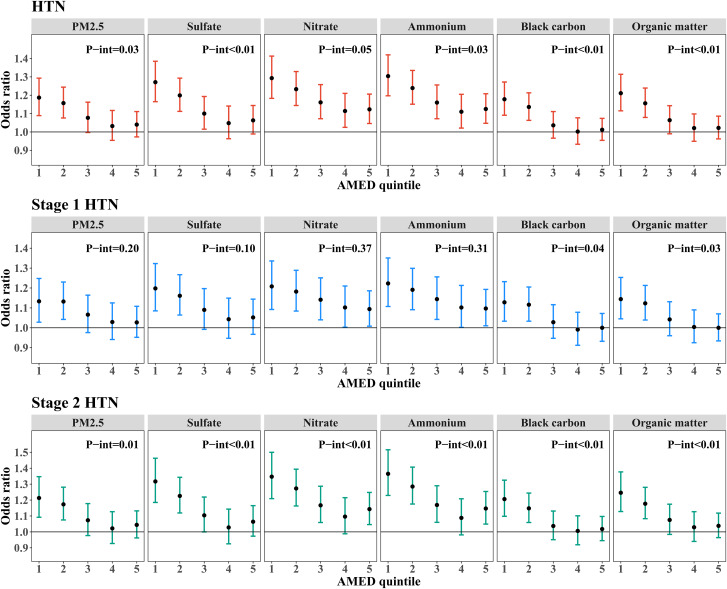
Odds ratios and 95% confidence intervals of HTN, stage 1 HTN, and stage 2 HTN associated with PM_2.5_ and its constituents by quintiles of AMED score. Note: Fully models were adjusted for age, sex, education, marital status, annual household income, smoking status, passive smoking, alcohol status, physical activity, outdoor time, BMI, household solid fuel use, HTN family history, residence, and region. *P*-int indicates the *P* values of the modifying effects of AMED score. Abbreviations: HTN, hypertension; PM_2.5_, fine particulate matter; IQR, interquartile range; AMED, alternative Mediterranean diet

Figure 3 (and Table S8) displays the interaction between DASH score and air pollution on HTN, stage 1 HTN, and stage 2 HTN. As the DASH quintiles rose, the relations between PM_2.5_ and its components and the risk of HTN weakened (*P*-interaction < 0.01), and these associations became statistically insignificant in the Q5 of DASH score. For per IQR increase in PM_2.5_, ${\text{SO}}_4^{2-}$, ${\text{NO}}_3^{-}$, ${\text{NH}}_4^{+}$, BC, and OM, the participants in the Q1 of DASH score had HTN risks with ORs (95% CI) of 1.20 (1.10, 1.30), 1.27 (1.17, 1.38), 1.30 (1.20, 1.42), 1.30 (1.20, 1.41), 1.17 (1.09, 1.26), and 1.20 (1.11, 1.30), whereas those in the largest DASH quintiles had lower HTN risks with ORs (95% CI) of 0.98 (0.91, 1.05), 1.00 (0.93, 1.09), 1.06 (0.99, 1.15), 1.06 (0.98, 1.15), 0.96 (0.90, 1.02), and 0.97 (0.91, 1.04). Similarly, we also found that the DASH score modified the relation between PM_2.5_ and its constituents with stage 1 HTN (*P*-interaction < 0.05) and stage 2 HTN (*P*-interaction < 0.01), with these associations becoming attenuated as the DASH score increasing. As shown in Fig. 4 (and Table S9), the associations between PM_2.5_ and its five constituents and risks of HTN and stage 2 HTN reduced with increasing quintiles of AMED score (except for ${\text{NO}}_3^{-}$-HTN association, all the *P*-interaction < 0.05). Similarly, the associations of BC and OM with stage 1 HTN were reduced with higher AMED score quintiles. Although the relationships between PM_2.5_ and its other three chemical components and stage 1 HTN risk trended downward across the AMED quintiles, the interactions were not statistically significant (*P*-interaction were from 0.37 for ${\text{NO}}_3^{-}$ to 0.10 for ${\text{SO}}_4^{2-}$).

**Fig. 5:**
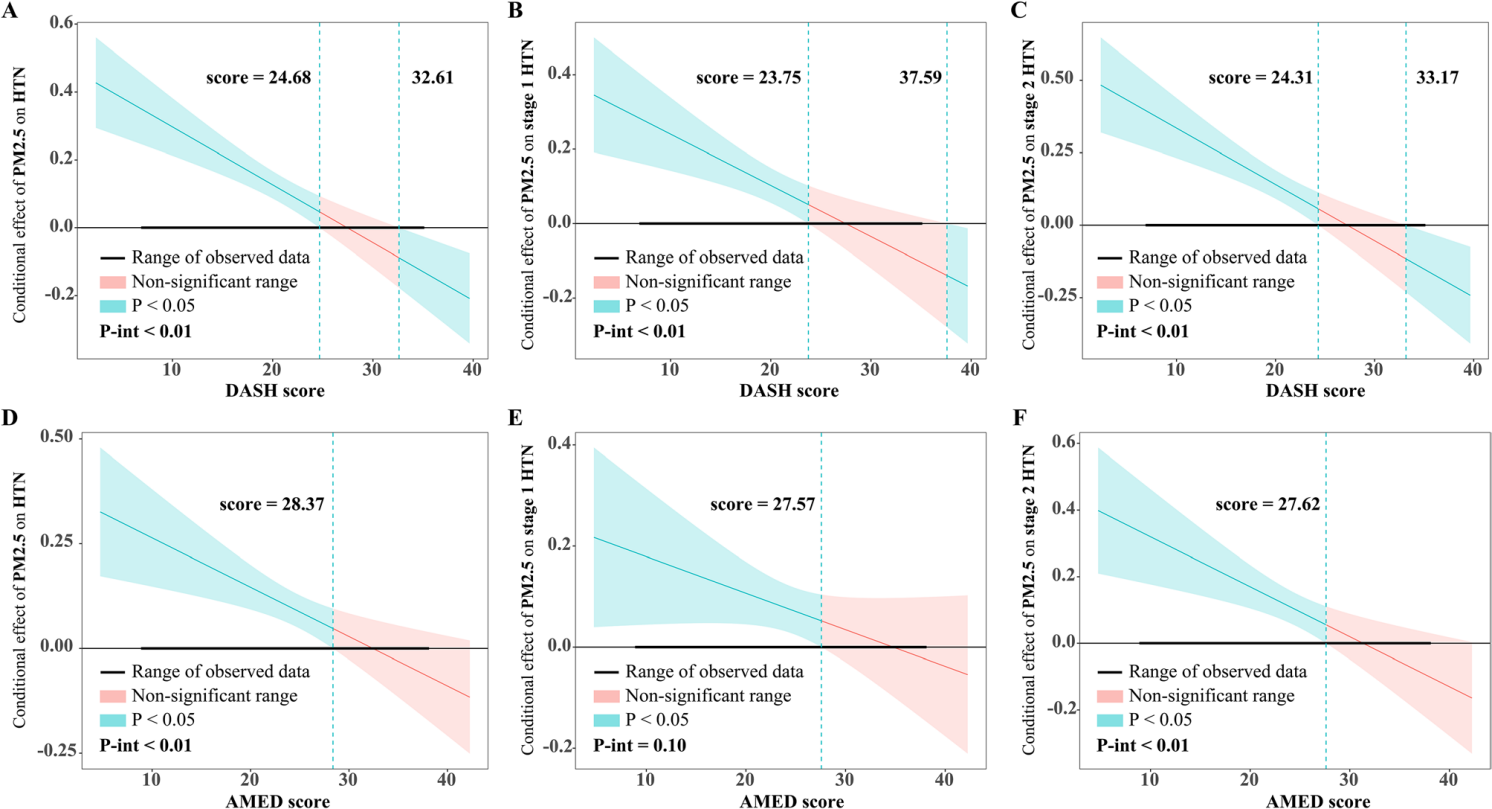
The Johnson-Neyman plots of the modifying effects of DASH and AMED scores on the relationship between long-term exposure to PM_2.5_ and risk of HTN, stage 1 HTN, and stage 2 HTN. Note: Fully models were adjusted for age, sex, education, marital status, annual household income, smoking status, passive smoking, alcohol status, physical activity, outdoor time, BMI, household solid fuel use, HTN family history, residence, and region. PM_2.5_ concentration or dietary scores were included in the model as continuous variables. *P*-int indicates the *P* values of the modifying effects of DASH/AMED score. Abbreviations: HTN, hypertension; PM_2.5_, fine particulate matter; IQR, interquartile range; DASH, dietary approaches to stop hypertension. AMED, alternative Mediterranean diet

Table S10 shows that when DASH/AMED scores were included in the model as continuous variables, there were still statistically significant interactions between dietary patterns and air pollution (*P*-interaction < 0.01), except for the interaction of AMED with PM_2.5_ (*P*-interaction = 0.10), ${\text{NO}}_3^{-}$ (*P*-interaction = 0.20), ${\text{NH}}_4^{+}$ (*P*-interaction = 0.14) on the risk of stage 1 HTN. Figure 5 presents the Johnson-Neyman plots of the interaction between dietary patterns and PM_2.5_ exposure. The analysis indicated that the association between PM_2.5_ exposure and risk of HTN, stage 1 HTN, and stage 2 HTN became weaker as DASH/AMED scores increased, even becoming non-significant at a certain level. Furthermore, the adverse effects of PM_2.5_ were even reversed when the DASH score was sufficiently high (score > 32.61 for HTN, score > 33.17 for stage 2 HTN), which was not observed in the AMED score. The Johnson-Neyman plots of the interaction of PM_2.5_ constituents with dietary scores can be seen in Fig. S2 and Fig. S3, with similar results to the interaction between PM_2.5_ and dietary scores.

Figure S4 depicts the estimates of DASH-modified relationships between PM_2.5_ and its components and HTN categories across different age groups. The results suggested that the interaction between DASH score and PM_2.5_ and its constituents on HTN, stage 1 HTN, and stage 2 HTN remained statistically significant in the < 65 years age group but not in the ≥ 65 years age group. A similar pattern to all participants was observed for the significant interaction between AMED score and PM_2.5_ and its constituents on HTN, stage 1 HTN, and stage 2 HTN in the < 65 years age group, whereas all the interactions were statistically non-significant in the ≥ 65 years age group (Fig. S5).

Additionally, we examined whether a single dietary component altered the association between air pollution and HTN classification. For HTN (Fig. S6 and Table S11) and stage 2 HTN (Fig. S8 and Table S13), adverse effects of PM_2.5_ and its five components were significantly decreased in participants who consumed more fruits, vegetables, dairy, and whole grains, except for the effect modification of the ${\text{NO}}_3^{-}$-HTN, ${\text{NO}}_3^{-}$-stage 2 HTN, ${\text{NH}}_4^{+}$-HTN, and ${\text{NH}}_4^{+}$-stage 2 HTN associations by vegetable score, ${\text{NO}}_3^{-}$-HTN and ${\text{NO}}_3^{-}$-stage 2 HTN associations by dairy score, and the BC-stage 2 HTN association by whole grain score. However, for stage 1 HTN (Fig. S7 and Table S12), the PM_2.5_-, SO^2-^_4_-, ${\text{NO}}_3^{-}$-, and ${\text{NH}}_4^{+}$- stage 1 HTN associations were significantly decreased in participants with consumed more whole grains, and those who consumed more dairy had a lower BC-stage 1 HTN and OM-stage 1 HTN associations.

We found that the relations between PM_2.5_ and its constituents with HTN, stage 1 HTN, and stage 2 HTN remained robust when using average concentrations of PM_2.5_ and its components for 1-, 2-, and 4-year exposure windows (Table S14). After further adjusting for preexisting diseases, the results showed that the interactions between DASH and AMED with PM_2.5_ and its chemical constituents on HTN, stage 1 HTN, and stage 2 HTN were still robust (Fig. S9). The results were also similar in the two-pollutant model with additional adjustment for ozone concentrations (Fig. S10), except that the effect modification of NO^-^_3_-HTN by AMED score became statistically significant. Even after including participants who had previously been diagnosed with HTN in this study, we observed that the DASH and AMED scores altered the correlations between PM_2.5_ and its constituents and HTN (Fig. S11).

## Discussion

In this nationwide study including 47,501 Chinese adults aged 18 ~ 79 years, we found that increased risk of HTN, stage 1 HTN, and stage 2 HTN were associated with long-term exposure to PM_2.5_ and its constituents and that the DASH and Mediterranean diets generally modified these associations. In particular, the effect modifications of the DASH diet in PM_2.5_ and its components-induced stage 1 HTN remained significant, as those with a higher DASH score had a significantly lower risk of stage 1 HTN. However, subgroup analyses revealed that the above-mentioned modifying effects were age-specific, with statistical significance observed only in the < 65 age group. Moreover, in the single dietary component analyses, consuming more fruits, vegetables, whole grains, and dairy produce decreased the incidence of HTN and stage 2 HTN caused by PM_2.5_ and its constituents, with stage 1 HTN risk significantly attenuated with increasing whole grains. The results provide an approach to implementing dietary interventions at the individual level to prevent earlier HTN caused by air pollution, especially in young and middle-aged individuals.

There is growing evidence that long-term exposure to PM_2.5_ increases the risk of HTN, especially in areas of high exposure [8, 9, 13, 47]. However, the above studies were based on the traditional definition of HTN (SBP/DBP ≥ 140/90 mmHg). In 2017, the ACC/AHA published a new HTN guideline, defining HTN as SBP/DBP ≥ 130/80 mmHg [26]. A stricter definition of HTN emphasized the importance of focusing on the earlier stages of HTN. In this study, PM_2.5_ exposure could significantly increase the risk of HTN, stage 1 HTN, and stage 2 HTN defined by the new guideline, and the association between PM_2.5_ and stage 2 HTN was stronger than for stage 1 HTN. There are few studies on the association between PM_2.5_ and the newly defined HTN. A cohort study covering the Beijing-Tianjin-Hebei region of China showed that every 10 µg/m^3^ of PM_2.5_ concentration could increase 5% risk of stage 1 HTN and 7% risk of stage 2 HTN, which is consistent with our results [48]. Our results were supported by the previous epidemiological studies showing a linear or log-linear association between PM_2.5_ and SBP/DBP levels [49–51]. It is worth noting that according to the traditional definition of HTN, there is also a significant association between prehypertension and air pollutants. A cross-sectional study based on 33 communities in China found that long-term exposure to ambient air pollution was more strongly associated with elevated BP in participants with prehypertension (SBP/DBP: 120 ~ 139/80 ~ 89 mmHg) than those with HTN (SBP/DBP ≥ 140/90 mmHg) [52]. Another study exploring the relationship between household air pollution and prehypertension and HTN had similar results [53]. The above results were inconsistent with ours, possibly because prehypertension includes stage 1 HTN and the different air pollutants in the study. These results highlighted the need for early intervention to prevent elevated blood pressure from PM_2.5_ exposure.

Like PM_2.5_, our study showed that chronic exposure to PM_2.5_ constituents was also significantly linked with HTN, stage 1 HTN, and stage 2 HTN, with ${\text{NO}}_3^{-}$ and ${\text{NH}}_4^{+}$ being the most substantial adverse effect, followed by ${\text{SO}}_4^{2-}$, OM, and BC. To our knowledge, only one study has examined the relationship between PM_2.5_ components and stage 1 HTN, which showed that PM_2.5_ components (${\text{NO}}_3^{-}$, ${\text{NH}}_4^{+}$, ${\text{SO}}_4^{2-}$, OM, and BC) were positively associated with the risk of stage 1 HTN, with the BC being the strongest adverse effect [54]. Due to the different units of ORs, the results between the two studies lacked comparability. In addition, several epidemiological studies have shown that PM_2.5_ constituents were associated with elevated BP and HTN. However, those results were inconsistent across different studies. A cohort study that included 14,331 Chinese adults showed that ${\text{SO}}_4^{2-}$ had the strongest effect on HTN, while OM had no significant effect [14]. Another national multi-center study in Chinese adults showed that OM, ${\text{NO}}_3^{-}$, and ${\text{NH}}_4^{+}$ were associated with DBP; BC and ${\text{NO}}_3^{-}$ were associated with SBP, and there was no association between ${\text{SO}}_4^{2-}$ and BP levels [15]. Differences in the chemical composition of PM_2.5_ across regions and time and differences in sensitivity to chemical composition across populations may have contributed to the heterogeneity of results across studies. Although the results of these studies were inconsistent, they all suggested that PM_2.5_ components contributed to the adverse effects of elevated BP.

The possible mechanism by which air pollution exposure leads to HTN is that they enter the body through the respiratory tract and cause oxidative stress and systemic inflammatory responses, which may lead to increased sympathetic nerves, affect hemodynamic responses, arterial remodeling, endothelial dysfunction, and thrombosis [55–61]. When these reactions are repeated, they may lead to increased total peripheral resistance and increased blood pressure [62].

Previous experimental and epidemiological research revealed that antioxidants and anti-inflammatory chemicals in the diet could help to mitigate the negative effects of air pollution on the cardiovascular system [19, 63, 64]. Similar mechanistic pathways provided a biological basis for implementing dietary interventions to minimize the harmful consequences of air pollution. Our results found that adherence to the DASH and AMED diets could reduce the risk of HTN, stage 1 HTN, and stage 2 HTN caused by PM_2.5_ and its constituents. The DASH diet also modified air pollution-stage 1 HTN association, with a lower risk of stage 1 HTN in those with higher DASH scores. Although the risk of stage 1 HTN decreased with the AMED score, the interaction between air pollution and the AMED score was insignificant, suggesting that the effect modification of the DASH diet was stronger than that of the AMED diet. Although DASH and AMED share several dietary components, several components differ, which may account for the different effects of the two dietary patterns. In single-component analyses, all seven items of the DASH score observed the protective effect against stage 1 HTN, with dairy products making the highest contribution. However, the major component of AMED that play a significant role against stage 1 HTN was legumes and nuts. By contrast, MUFA: SFA and red meat even showed harmful effects. Similar results were found investigating the association between HTN and dietary patterns in a multi-ethnic population in southwest China [36]. These results could partly explain the difference in the degree of modification of air pollution-induced stage 1 HTN by DASH and AMED scores.

DASH and Mediterranean diets were proven healthy dietary patterns rich in anti-inflammatory substances and antioxidants, such as vitamin D, vitamin E, vitamin C, B vitamins, omega-3 polyunsaturated fatty acids, and various phytochemicals [65–67]. These compounds may alleviate the inflammatory response and oxidative stress caused by air pollution exposure. Previous mechanistic research has found several possible biological pathways for diet-modifying effects. Animal studies have shown that supplementation with B vitamins, vitamin E, omega-3 fatty acids, vitamin D_3_ and blueberry anthocyanins helped to increase antioxidant levels, decrease inflammatory cytokine levels, reduce cardiomyocyte damage and improved electrocardiographic abnormalities in PM_2.5_-exposure rats [68–71]. Similar results were found in a randomized controlled trial where vitamin C, vitamin E and B-vitamin supplementation reduced elevated indicators of oxidative stress and altered gene methylation due to particulate matter exposure in adults [72, 73].

There is limited epidemiological evidence on whether dietary patterns can affect the relationship between air pollution and the occurrence of HTN. One study has examined the interaction of the DASH diet and particulate matter (PM) on HTN and found that adults in southwest China who followed the DASH diet had a lower risk of PM-induced HTN, which is consistent with our results [25]. Notably, our study population was a nationally representative sample of Chinese adults, which addressed the applicability of the findings on the effect modification of healthy eating patterns on the association of air pollution with HTN to the rest of China. Furthermore, we also found that the DASH diet altered the association of PM_2.5_ and its compositions with stage 1 HTN, which provided evidence for moving the dietary intervention point forward. Past studies exploring the interaction between dietary patterns and air pollution on cardiovascular disease mortality also support our results, as HTN is a major cause of cardiovascular disease [74]. A prospective cohort study (n = 548,845) based on the National Institutes of Health-American Association of Retired Persons (NIH-AAPP) Diet and Health Study showed that adherence to the Mediterranean diet could attenuate cardiovascular disease mortality associated with exposure to PM_2.5_ and nitrogen dioxide (NO_2_) [75]. An analysis of the UK Biobank study showed that a healthy diet (including vegetables, fruits, unprocessed red meat, fish, and processed meat) modified the relation between PM_2.5_, NO_X_, NO_2_, and all-cause death, but no significant interaction was found between air pollution and dietary eating patterns and mortality from CVD, which is inconsistent with our results [76]. Such discrepancy may be due in part to differences in the components of dietary patterns, which further suggested that only a diet consisting of a variety of dietary components rich in antioxidants and anti-inflammatory compounds may be able to protect the cardiovascular system from air pollution. In addition, the study based on the Nurses’ Health Study (NHS) did not find a significant interaction between the DASH diet and long-term exposure to PM_2.5_ on HTN, which is inconsistent with our results [23]. The difference in results may be mainly because the participants in the NHS study were a particular group of female nurses with narrow socioeconomic status and low obesity in the United States. Their PM_2.5_ exposure levels were much lower than those of the Chinese population. (Mean ± SD of PM_2.5_ exposure: 15.61 ± 4.24 µg/m^3^ vs. 53.4 ± 23.0 µg/m^3^). Another cohort study conducted in a Spanish population showed that the Mediterranean diet did not modify the relation between long-term exposure to PM_2.5_ and incident of HTN [24]. However, the sample size of this study ( n= 1,103) was small, and the PM_2.5_ exposure levels of subjects were much lower than those of our subjects. More large cohort studies are needed in the future to address this discrepancy.

In single-component analyses, we found that individuals who consumed more vegetables, fruits, dairy products, and whole grains had lower relations between air pollution and HTN and stage 2 HTN. The statistically significant modifying effect demonstrated by dairy products could further explain why the DASH diet is preferable to the Mediterranean diet. Additionally, significant modulation of air pollution-stage 1 HTN association by a single dietary component was observed only in whole grains and fruits, suggesting limited improvement from an individual dietary component. Due to the lack of comprehensive dietary information, there is insufficient epidemiological evidence to support the modifying effect of dairy and whole grains on the connection between air pollution and cardiovascular health. However, our results are consistent with the China Multi-Ethnic Cohort [25]. One analysis from the NIH-APP Diet and Health Study found that whole grains reduced PM_2.5_-induced cardiovascular disease mortality [75]. Dairy and whole grains are also abundant in anti-inflammatory substances and antioxidants such as B vitamins, vitamin A, vitamin D, and vitamin E. Several nutrient intervention studies also support our findings [72, 73]. The ameliorative effect of vegetables and fruits on cardiovascular diseases caused by air pollution has been widely demonstrated [22, 64, 71, 75, 62].

However, the results of subgroup analyses showed that the significant interaction between air pollution and the dietary pattern was observed only in the < 65 years age group (*P*-interaction < 0.05). For participants older than 65 years, the risks of HTN, stage 1 HTN, and stage 2 HTN associated with PM_2.5_ and its constituents decreased with increasing DASH and AMED scores, although the interaction effects between dietary patterns and air pollution were not statistically significant. Notably, the relation between air pollution and HTN categories stratified by DASH and AMED quintiles was statistically insignificant in the > 65 years age group. This result may be due to the age-specific association of air pollution with HTN. Therefore, we explored the relationship between air pollution and HTN status stratified by age group and found that the associations between PM_2.5_ and its chemical components and HTN, stage 1 HTN, and stage 2 HTN were statistically significant in the < 65 years age group, but not in the ≥ 65 years age group (Table S15). Several previous studies have also not found an association between long-term exposure to PM_2.5_ and HTN in the elderly [9, 23, 77–79], consistent with our findings. Age is the main influencing factor of elevated blood pressure, and the prevalence of HTN in the elderly is significantly higher than that in the young and middle-aged, which may lead to the lack of observed relation between PM_2.5_ exposure and the risk of HTN in the elderly. In addition, another reason may be since older adults spend more time indoors, and outdoor ambient PM_2.5_ exposure at residences commonly used in past analyses may not be a major source of PM_2.5_ exposure in older adults. More epidemiological studies are needed on the interaction of air pollution and dietary patterns on cardiovascular disease in the elderly.

This study has several strengths. First, our study population is a nationally representative sample with a wide range of air pollution exposures. Our findings can be used as a reference for LMICs with high air pollution levels. Second, we collected a wide range of detailed individual-level information during the survey, such as lifestyle, diet habits, physical activity, and household fuel information, which allowed us to adjust as fully as possible for confounding variables. Third, we used generally used index scores to quantify diet quality and explored whether diets reduce the harmful effects of air pollution on cardiovascular health in terms of both dietary patterns and dietary components. These findings could be more easily translated into dietary intervention guidelines or policies. Finally, we revealed a modifying effect of dietary patterns on stage 1 HTN caused by PM_2.5_ and its components, which provided a basis for implementing early dietary interventions at the individual level to prevent HTN and elevated BP.

However, there are also some limitations in this study. First, the cross-sectional design limits the interpretation of the causal relationship between air pollution and incident of HTN. Second, the average concentration of residents for three years may not reflect the actual exposure level of an individual to PM_2.5_ and its constituents, leading to exposure misclassification. Third, there have been inevitable recall biases in the dietary data collected by the FFQ. Fourth, although we have included as many potential confounders as possible, some variables are still missing, such as psychosocial stress, personal protective equipment, and traffic noise. Finally, the strong correlations among constituents did not allow us to interpret independent associations from single components on HTN, and our exposure assessment approach failed to address this limitation.

## Conclusions

In summary, healthy dietary patterns can reduce long-term exposure to PM_2.5_ and its constituents-induced HTN as defined by the 2017 ACC/AHA guideline, especially in young and middle-aged individuals. Compared to the Mediterranean diet, DASH diet offers superior dietary guidance to prevent stage 1 HTN caused by air pollution. Adequate intake of foods rich in antioxidants can reduce the risk of HTN associated with air pollution. These findings suggested that adherence to an antioxidant and anti-inflammatory dietary pattern may be a viable strategy for individual-level dietary interventions to reduce the effects of air pollution on the earlier stage of HTN.

### Electronic supplementary material

Below is the link to the electronic supplementary material.

Supplementary Material 1

## Data Availability

The datasets generated and/or analyzed during the current study are not publicly available but are available from the corresponding author on reasonable request.

## Abbreviations

HTN: Hypertension
LMICs: Low- and Middle-Income Countries
PM_2.5_: Fine Particulate Matter
BP: Blood Pressure
DASH: Dietary Approaches to Stop Hypertension
ACC: American College of Cardiology
AHA: American Heart Association
SBP: Systolic Blood Pressure
DBP: Diastolic Blood Pressure
CVD: Cardiovascular Disease
CNHS: China Nutrition and Health Surveillance
CCDC: Chinese Center for Disease Control and Prevention
FFQ: Food Frequency Questionnaire
AMED: Alternative Mediterranean Diet
MUFA: Monounsaturated Fat Acid
SFA: Saturated Fat Acid
TAP: Tracking Air Pollution in China
CMAQ: Community Multiscale Air Quality
SO_4_^2-^: Sulfate
NO_3_^-^: Nitrate
NH_4_^+^: Ammonium
BC: Black Carbon
OM: Organic Matter
BMI: Body Mass Index
SD: Standard Deviations
ORs: Odds Ratios
CIs: Confidence Intervals
