# Supplementary material for: Association of fine particulate matter and its constituents with hypertension: the modifying effect of dietary patterns
Source: Environ Health. 2023 Aug 9;22:55. doi: 10.1186/s12940-023-01000-y (PMC10411005; doi:10.1186/s12940-023-01000-y)
Supplement: Supplementary file 1 — Supplementary Material 1 [file 12940_2023_1000_MOESM1_ESM.docx]

**Supplementary materials**

**Table S1:** Scoring criteria for the DASH and AMED score.

**Table S2:** Exchange factors of fatty acids for food groups.

**Table S3:** Diagnosis of multicollinearity in logistic regression models of the relationship between PM_2.5_ and HTN, stage 1 HTN, and stage 2 HTN.

**Table S4:** Characteristics of participants by PM_2.5_ quartiles.

**Table S5:** ORs and 95% CI for HTN, stage 1 HTN, and stage 2 HTN associated with a IQR increase in PM_2.5_ and its constituents, stratified by age.

**Table S6:** ORs associated with 25% score increment in DASH score and after alternate subtraction of each of its dietary components.

**Table S7:** ORs associated with 25% score increment in AMED score and after alternate subtraction of each of its dietary components.

**Table S8:** ORs and 95% CI associated with per IQR increase in PM_2.5_ and its constituents by quintiles of DASH score.

**Table S9:** ORs and 95% CI associated with per IQR increase in PM_2.5_ and its constituents by quintiles of AMED score.

**Table S10:** ORs (95% CI) and *P* values for the interaction between dietary patterns and PM_2.5_ and its components, keeping PM_2.5_ and its components and DASH/AMED scores as continuous variables.

**Table S11:** ORs and 95% CI of hypertension risk associated with per IQR increase in PM_2.5_ and its constituents by quintiles of dietary components score.

**Table S12:** ORs and 95% CI of stage 1 hypertension risk associated with per IQR increase in PM_2.5_ and its constituents by quintiles of dietary components score.

**Table S13:** ORs and 95% CI of stage 2 hypertension risk associated with per IQR increase in PM_2.5_ and its constituents by quintiles of dietary components score.

**Table S14:** ORs and 95% CI of hypertension, stage 1hypertension, and stage 2 hypertension associated with particulate matter by different exposure windows.

**Table S15:** ORs and 95% CI for HTN, stage 1 HTN, and stage 2 HTN associated with a IQR increase in PM_2.5_ and its constituents, stratified by age.

**Fig. S1:** Flowchart of the study population.

**Fig. S2:** The Johnson-Neyman plots of the modifying effects of DASH scores on the relationship between long-term exposure to PM2.5 constituents and risk of HTN, stage 1 HTN, and stage 2 HTN.

**Fig. S3:** The Johnson-Neyman plots of the modifying effects of AMED scores on the relationship between long-term exposure to PM2.5 constituents and risk of HTN, stage 1 HTN, and stage 2 HTN.

**Fig. S4:** ORs and 95% CI of stage 1 hypertension risk associated with per IQR increase in PM_2.5_ and its constituents by quintiles of DASH score in different age groups.

**Fig. S5:** ORs and 95% CI of stage 1 hypertension risk associated with per IQR increase in PM_2.5_ and its constituents by quintiles of AMED score in different age groups.

**Fig. S6:** ORs and 95% CI of hypertension risk associated with per IQR increase in PM_2.5_ and its constituents by quintiles of dietary component score.

**Fig. S7:** ORs and 95% CI of stage 1 hypertension risk associated with per IQR increase in PM_2.5_ and its constituents by quintiles of dietary component score.

**Fig. S8:** ORs and 95% CI of stage 2 hypertension risk associated with per IQR increase in PM_2.5_ and its constituents by quintiles of dietary component score.

**Fig. S9:** ORs and 95% CI associated with PM_2.5_ and its constituents by quintiles of DASH and AMED scores, and the model was further adjusted for preexisting diseases.

**Fig. S10:** ORs and 95% CI associated with PM_2.5_ and its constituents by quintiles of DASH and AMED scores, and the model was further adjusted for ozone concentrations.

**Fig. S11:** ORs and 95% CI associated with PM_2.5_ and its constituents by quintiles of DASH and AMED scores after including the participants diagnosed with hypertension into analyses.

# Table S1 Scoring criteria for the DASH and AMED score.

| **Dietary components** | **Foods** | **Criteria** | | |
| --- | --- | --- | --- | --- |
| Fresh vegetables | All fresh vegetables | The daily food intake of the study population was divided into quintiles  Q1=1 point  Q2=2 points  Q3=3 points  Q4=4 points  Q5=5 points | | |
| Fruits | Fresh fruits, dried fruits (non-processed) |  |  |  |
| Legumes and nuts | Soybeans, mixed beans (black beans/red beans/mung beans, etc.), bean products (tofu/soybean milk/dried beans/dried bean curd, etc.), nuts (peanuts/melon seeds/walnuts, etc.) |  |  |  |
| Dairy products | Full fat liquid milk, low fat skimmed liquid milk, whole milk powder, low fat milk powder, yoghurt, cheese |  |  |  |
| Whole grains | Maize and its products (corn flour/corn ballast, etc.), other coarse grains and products (buckwheat/millet, etc.) |  |  |  |
| Fish | Fish, prawns, crab, mollusks (squid/ shellfish/ snails/ sea cucumbers, etc.) |  |  |  |
| MUFA: SFA | From all kinds of foods and condiments |  |  |  |
| Red and processed meat | Pork, beef, mutton, other red meat (donkey / horse / rabbit etc.), red meat products | Reverse score | | |
| Sodium | Salt, condiments, and all foods |  |  |  |
| Alcohol | All alcoholic beverages | Moderate alcohol intake (g/day) | | score |
|  |  | For male | For female |  |
|  |  | (10, 30] | (5, 15] | 5 points |
|  |  | (0, 10] or (30, 40] | (0, 5] or (15, 25] | 4 points |
|  |  | 0 or (40, 45) | 0 or (25, 30] | 3 points |
|  |  | (45, 50) | (30, 35) | 2 points |
|  |  | >50 | >35 | 1 point |

Abbreviation: DASH for Dietary Approaches to Stop Hypertension; AMED for alternative Mediterranean diet; Q for quintiles; MUFA: SFA for the ratio of monounsaturated fatty acids to saturated fatty acids.

DASH score: Fresh vegetables, fruits, legumes and nuts, dairy products, whole grains, red and processed meat, and sodium

ANED score: Fresh vegetables, fruits, legumes and nuts, whole grains, fish, MUFA: SFA, red and processed meat, and Alcohol

# Table S2 Exchange factors of fatty acids for food groups.

| **Food groups** | **Unit (g)** | **SFA (g)** | **MUFA (g)** | **PUFA (g)** | **MUFA: SFA** |
| --- | --- | --- | --- | --- | --- |
| Rice and products | 100 | 0.30 | 0.20 | 0.30 | 0.67 |
| Wheat and products | 100 | 0.33 | 0.20 | 0.23 | 0.60 |
| Corn and products | 100 | 0.33 | 0.43 | 0.98 | 1.31 |
| Other cereals and products | 100 | 0.86 | 1.14 | 0.68 | 1.33 |
| Mixed beans | 100 | 0.17 | 0.17 | 0.48 | 1.00 |
| Potatoes | 100 | 0.00 | 0.10 | 0.10 | - |
| Fried pasta | 100 | 2.60 | 5.30 | 5.65 | 2.04 |
| Soybeans | 100 | 2.50 | 4.07 | 8.33 | 1.63 |
| Soybean milk | 100 | 0.45 | 0.35 | 0.35 | 0.78 |
| Tofu | 100 | 2.00 | 1.80 | 1.10 | 0.90 |
| Yuba | 100 | 5.43 | 6.60 | 8.57 | 1.21 |
| Shredded bean curd | 100 | 2.98 | 3.74 | 7.24 | 1.26 |
| Fresh Mushrooms | 100 | 0.00 | 0.00 | 0.15 | - |
| Dried mushrooms | 100 | 0.31 | 0.51 | 0.87 | 1.64 |
| Auricularia and tremella | 100 | 0.70 | 0.45 | 0.55 | 0.64 |
| Fresh Kelp | 100 | 2.50 | 2.60 | 0.70 | 1.04 |
| Dried Kelp and Nori | 100 | 0.60 | 0.15 | 0.10 | 0.25 |
| Fresh fruits | 100 | 2.87 | 0.34 | 0.23 | 0.12 |
| Dried fruits | 100 | 0.35 | 0.35 | 1.88 | 1.00 |
| Full fat liquid milk | 100 | 2.10 | 1.00 | 0.10 | 0.48 |
| Low fat, skimmed liquid milk | 100 | 1.00 | 0.40 | 0.00 | 0.40 |
| Whole milk powder | 100 | 12.00 | 6.20 | 1.10 | 0.52 |
| Low fat milk powder | 100 | 7.50 | 2.60 | 0.20 | 0.35 |
| Yoghurt | 100 | 1.60 | 0.60 | 0.10 | 0.38 |
| Cheese | 100 | 14.80 | 5.30 | 0.93 | 0.36 |
| Pork | 100 | 10.80 | 13.30 | 2.10 | 1.23 |
| Beef | 100 | 4.10 | 3.50 | 0.30 | 0.85 |
| Mutton | 100 | 4.20 | 2.40 | 0.80 | 0.57 |
| Poultry | 100 | 4.73 | 7.73 | 2.97 | 1.63 |
| Other meat | 100 | 1.42 | 1.73 | 0.84 | 1.22 |
| Meat products | 100 | 9.59 | 11.53 | 2.87 | 1.20 |
| Animal Offal | 100 | 2.22 | 1.90 | 0.75 | 0.86 |
| Marine fish | 100 | 1.04 | 1.26 | 0.68 | 1.21 |
| Freshwater fish | 100 | 0.95 | 1.09 | 0.79 | 1.15 |
| Shrimp | 100 | 0.45 | 0.48 | 0.34 | 1.06 |
| Crab | 100 | 0.29 | 0.32 | 0.32 | 1.10 |
| Mollusks | 100 | 0.60 | 0.42 | 0.26 | 0.70 |
| Fresh eggs | 100 | 3.78 | 4.42 | 0.86 | 1.17 |
| Salted eggs | 100 | 3.95 | 6.20 | 0.55 | 1.57 |
| Dried eggs | 100 | 3.40 | 4.30 | 0.85 | 1.26 |
| Nuts | 100 | 6.73 | 19.39 | 19.40 | 2.88 |
| Vegetable oils | 100 | 12.49 | 39.66 | 41.68 | 3.18 |
| Animal oils | 100 | 42.90 | 39.75 | 7.83 | 0.93 |

Abbreviation: SFA: saturated fatty acids; MUFA: monounsaturated fatty acids; PUFA: polyunsaturated fatty acids; MUFA: SFA for the ratio of monounsaturated fatty acids: saturated fatty acids

# Table S3 Diagnosis of multicollinearity in logistic regression models of the relationship between PM2.5 and HTN, stage 1 HTN, and stage 2 HTN.

| **Independent variables** | **HTN** | | |  | **Stage 1 HTN** | | |  | **Stage 2 HTN** | | |
| --- | --- | --- | --- | --- | --- | --- | --- | --- | --- | --- | --- |
|  | **GVIF** | **DF** | **GVIF^(1/(2*DF))^** |  | **GVIF** | **DF** | **GVIF^(1/(2*DF))^** |  | **GVIF** | **DF** | **GVIF^(1/(2*DF))^** |
| PM_2.5_ | 1.64 | 1 | 1.28 |  | 1.66 | 1 | 1.29 |  | 1.62 | 1 | 1.27 |
| Age | 1.47 | 1 | 1.21 |  | 1.47 | 1 | 1.21 |  | 1.44 | 1 | 1.20 |
| Sex | 2.11 | 1 | 1.45 |  | 2.08 | 1 | 1.44 |  | 2.09 | 1 | 1.45 |
| Education | 1.74 | 4 | 1.07 |  | 1.75 | 4 | 1.07 |  | 1.67 | 4 | 1.07 |
| Marital status | 1.23 | 3 | 1.03 |  | 1.23 | 3 | 1.04 |  | 1.18 | 3 | 1.03 |
| Annual household income | 1.30 | 3 | 1.04 |  | 1.30 | 3 | 1.04 |  | 1.29 | 3 | 1.04 |
| Smoking status | 2.03 | 1 | 1.42 |  | 2.01 | 1 | 1.42 |  | 1.99 | 1 | 1.41 |
| Passive smoking | 1.10 | 1 | 1.05 |  | 1.10 | 1 | 1.05 |  | 1.10 | 1 | 1.05 |
| Alcohol status | 1.43 | 3 | 1.06 |  | 1.41 | 3 | 1.06 |  | 1.42 | 3 | 1.06 |
| Physical activity | 1.10 | 2 | 1.02 |  | 1.11 | 2 | 1.03 |  | 1.09 | 2 | 1.02 |
| Outdoor time | 1.26 | 4 | 1.03 |  | 1.28 | 4 | 1.03 |  | 1.25 | 4 | 1.03 |
| BMI | 1.07 | 3 | 1.01 |  | 1.06 | 3 | 1.01 |  | 1.12 | 3 | 1.02 |
| Household solid fuel use | 1.32 | 1 | 1.15 |  | 1.33 | 1 | 1.15 |  | 1.31 | 1 | 1.14 |
| HTN family history | 1.09 | 1 | 1.04 |  | 1.08 | 1 | 1.04 |  | 1.10 | 1 | 1.05 |
| Residence | 1.37 | 1 | 1.17 |  | 1.38 | 1 | 1.18 |  | 1.36 | 1 | 1.16 |
| Region | 1.89 | 6 | 1.05 |  | 1.91 | 6 | 1.06 |  | 1.87 | 6 | 1.05 |

Abbreviation: HTN, hypertension; GVIF, general variance inflation factor; DF, degree of freedom; PM_2.5_, fine particulate matter; BMI, body mass index.

# Table S4 Characteristics of participants by PM_2.5_ quartiles.

| **Characteristics** | **PM_2.5_ quintiles (μg/m^3^)** | | | | |
| --- | --- | --- | --- | --- | --- |
|  | **Q1 (13.4~32.6)** | **Q2 (32.7~42.0)** | **Q3 (42.0~56.8)** | **Q4 (56.9~73.9)** | **Q5 (73.9~116.5)** |
| **Eligible participants** | 9491 | 9513 | 9497 | 9505 | 9495 |
| Age, years (SD) | 49.6 (13.7) | 50.2 (13.6) | 51.1 (13.8) | 51.6 (14.2) | 49.8 (13.9) |
| SBP, mm Hg (SD) | 129.5 (18.8) | 131.3 (19.2) | 131.2 (19.1) | 132.1 (18.7) | 133 (18.7) |
| DBP, mm Hg (SD) | 77.5 (11.1) | 77.5 (10.9) | 77.4 (10.9) | 78.2 (10.6) | 78.9 (10.7) |
| **Dietary score (SD)** |  |  |  |  |  |
| DASH | 18.3 (4.4) | 19.2 (4.3) | 20.3 (4.6) | 21.1 (4.5) | 22.3 (4.5) |
| AMED | 22.2 (4.3) | 23.4 (4.1) | 24.1 (4.2) | 25.1 (3.9) | 25.9 (3.9) |
| Fruits | 2.8 (1.4) | 2.9 (1.4) | 3 (1.4) | 3.1 (1.4) | 3.2 (1.4) |
| Vegetables | 3.0 (1.4) | 3.0 (1.5) | 3.1 (1.4) | 3.2 (1.4) | 3.2 (1.5) |
| Dairy | 2.1 (1.7) | 2.2 (1.6) | 2.6 (1.8) | 2.7 (1.8) | 2.6 (1.8) |
| Whole grains | 2.2 (1.5) | 2.5 (1.6) | 2.7 (1.6) | 2.7 (1.6) | 3.5 (1.6) |
| Legumes and nuts | 2.7 (1.4) | 2.8 (1.4) | 3.0 (1.4) | 3.2 (1.4) | 3.4 (1.4) |
| Fish | 3.2 (1.4) | 2.8 (1.4) | 2.9 (1.4) | 3.2 (1.4) | 2.8 (1.4) |
| MUFA: SFA | 2.6 (1.4) | 3.2 (1.4) | 3.1 (1.4) | 3.1 (1.4) | 3.1 (1.4) |
| Alcohol | 3.3 (0.8) | 3.3 (0.8) | 3.3 (0.8) | 3.3 (0.8) | 3.3 (0.8) |
| Red meat | 2.5 (1.4) | 3.0 (1.4) | 3.0 (1.4) | 3.1 (1.4) | 3.3 (1.4) |
| Sodium | 3.0 (1.4) | 2.9 (1.4) | 2.9 (1.4) | 3.1 (1.4) | 3.1 (1.4) |
| **Gender (%)** |  |  |  |  |  |
| Male | 4469 (47.1) | 4582 (48.2) | 4397 (46.3) | 4335 (45.6) | 4351 (45.8) |
| Female | 5022 (52.9) | 4931 (51.8) | 5100 (53.7) | 5170 (54.4) | 5144 (54.2) |
| **Education level (%)** |  |  |  |  |  |
| Illiteracy | 2932 (30.9) | 3026 (31.8) | 2358 (24.8) | 2002 (21.1) | 1602 (16.9) |
| Primary school | 2131 (22.5) | 2135 (22.4) | 1937 (20.4) | 1697 (17.9) | 1693 (17.8) |
| Junior high school | 2932 (30.8) | 2871 (30.2) | 2961 (31.2) | 3084 (32.5) | 3462 (36.5) |
| High school | 1062 (11.2) | 994 (10.5) | 1276 (13.4) | 1582 (16.6) | 1692 (17.8) |
| Junior college and above | 434 (4.6) | 487 (5.1) | 965 (10.2) | 1140 (12.0) | 1046 (11.0) |
| **Marital status (%)** |  |  |  |  |  |
| Married or cohabiting | 8677 (91.4) | 8867 (93.2) | 8741 (92.0) | 8728 (91.8) | 8842 (93.1) |
| Widowed | 278 (2.9) | 236 (2.5) | 295 (3.1) | 279 (2.9) | 202 (2.1) |
| Separated or divorced | 70 (0.8) | 71 (0.8) | 66 (0.7) | 78 (0.9) | 67 (0.8) |
| Never married | 466 (4.9) | 339 (3.5) | 395 (4.2) | 420 (4.4) | 384 (4.0) |
| **Annual family income, CNY (%)** |  |  |  |  |  |
| <20,000 | 2147 (22.6) | 2217 (23.3) | 2084 (21.9) | 1668 (17.6) | 1646 (17.3) |
| 20,000-49,999 | 3340 (35.2) | 3372 (35.5) | 3201 (33.7) | 2989 (31.5) | 3432 (36.2) |
| ≥50,000 | 2068 (21.8) | 2137 (22.5) | 2956 (31.1) | 3590 (37.7) | 3187 (33.5) |
| Unknown | 1936 (20.4) | 1787 (18.8) | 1256 (13.3) | 1258 (13.2) | 1230 (13.0) |
| **BMI,** kg/m^2^ **(%)** |  |  |  |  |  |
| 18.5-23.9 | 5198 (54.8) | 5100 (53.6) | 4919 (51.8) | 4404 (46.3) | 3904 (41.1) |
| <18.5 | 560 (5.9) | 512 (5.4) | 406 (4.3) | 316 (3.4) | 209 (2.2) |
| 24-27.9 | 2837 (29.9) | 2981 (31.3) | 3126 (32.9) | 3508 (36.9) | 3685 (38.8) |
| ≥28 | 896 (9.4) | 920 (9.7) | 1046 (11.0) | 1277 (13.4) | 1697 (17.9) |
| **Smoking status (%)** |  |  |  |  |  |
| Never | 6229 (65.6) | 6324 (66.5) | 6364 (67.0) | 6470 (68.1) | 6559 (69.1) |
| Smoking or quit smoking | 3262 (34.4) | 3189 (33.5) | 3133 (33.0) | 3035 (31.9) | 2936 (30.9) |
| **Passive smoking (%)** |  |  |  |  |  |
| No | 2904 (30.6) | 3038 (31.9) | 3005 (31.6) | 3244 (34.1) | 3449 (36.3) |
| Yes | 6587 (69.4) | 6475 (68.1) | 6492 (68.4) | 6261 (65.9) | 6046 (63.7) |
| **Alcohol consumption (%)** |  |  |  |  |  |
| Never | 5895 (62.1) | 6197 (65.1) | 6085 (64.1) | 6140 (64.6) | 5862 (61.7) |
| ≤3 times/ month | 1686 (17.8) | 1668 (17.5) | 1690 (17.8) | 1564 (16.5) | 1760 (18.5) |
| 1-4 times/ week | 958 (10.1) | 777 (8.2) | 798 (8.4) | 812 (8.5) | 869 (9.2) |
| ≥5 times/ week | 952 (10.0) | 871 (9.2) | 924 (9.7) | 989 (10.4) | 1004 (10.6) |
| **Household solid-fuel use** |  |  |  |  |  |
| No | 5475 (57.7) | 4602 (48.4) | 5755 (60.6) | 6951 (73.1) | 7155 (75.4) |
| Yes | 4016 (42.3) | 4911 (51.6) | 3742 (39.4) | 2554 (26.9) | 2340 (24.6) |
| **Physical activity (%)** |  |  |  |  |  |
| Low | 1976 (20.8) | 2209 (23.2) | 2045 (21.5) | 2295 (24.2) | 2634 (27.7) |
| Moderate | 2038 (21.5) | 2031 (21.4) | 2303 (24.3) | 2739 (28.8) | 2751 (29.0) |
| High | 5477 (57.7) | 5273 (55.4) | 5149 (54.2) | 4471 (47.0) | 4110 (43.3) |
| **Outdoor time, min (%)** |  |  |  |  |  |
| Quintile 1 [0,75] | 1554 (16.4) | 1602 (16.8) | 2050 (21.6) | 2459 (25.9) | 2005 (21.1) |
| Quintile 2 [75,149] | 1552 (16.4) | 1646 (17.3) | 1795 (18.9) | 2220 (23.4) | 1961 (20.7) |
| Quintile 3 [150,238] | 1736 (18.2) | 1951 (20.5) | 1935 (20.4) | 1851 (19.5) | 2110 (22.2) |
| Quintile 4 [240,358] | 1927 (20.3) | 1978 (20.8) | 1846 (19.4) | 1481 (15.6) | 1807 (19.0) |
| Quintile 5 [360,750] | 2722 (28.7) | 2336 (24.6) | 1871 (19.7) | 1494 (15.6) | 1612 (17.0) |
| **HTN family history (%)** |  |  |  |  |  |
| Yes | 2129 (22.4) | 2326 (24.5) | 2813 (29.6) | 3149 (33.1) | 3442 (36.3) |
| No | 7362 (77.6) | 7187 (75.5) | 6684 (70.4) | 6356 (66.9) | 6053 (63.7) |
| **Residence (%)** |  |  |  |  |  |
| Urban | 2338 (24.6) | 3042 (32.0) | 4045 (42.6) | 5155 (54.2) | 4982 (52.5) |
| Rural | 7153 (75.4) | 6471 (68.0) | 5452 (57.4) | 4350 (45.8) | 4513 (47.5) |
| **Region (%)** |  |  |  |  |  |
| North | 640 (6.7) | 212 (2.2) | 1068 (11.3) | 1200 (12.6) | 3742 (39.4) |
| Northeast | 1225 (12.9) | 1408 (14.8) | 833 (8.8) | 1408 (14.8) | 140 (1.5) |
| East | 1713 (18.1) | 2300 (24.2) | 2593 (27.3) | 3580 (37.7) | 2522 (26.6) |
| Central | 7 (0.1) | 742 (7.8) | 1194 (12.6) | 1567 (16.5) | 2536 (26.7) |
| Southwest | 2321 (24.5) | 902 (9.5) | 1389 (14.5) | 1273 (13.4) | 551 (5.8) |
| Northwest | 589 (6.2) | 2302 (24.2) | 1726 (18.2) | 477 (5.0) | 4 (0) |
| South | 2996 (31.5) | 1647 (17.3) | 694 (7.3) | 0 (0) | 0 (0) |
| **HTN categories** |  |  |  |  |  |
| Without HTN | 4470 (47.1) | 4241 (44.6) | 4168 (43.9) | 3912 (41.2) | 3706 (39.0) |
| HTN | 5021 (52.9) | 5272 (55.4) | 5329 (56.1) | 5593 (58.8) | 5789 (61.0) |
| Stage1 HTN | 2449 (25.8) | 2434 (25.6) | 2450 (25.8) | 2602 (27.4) | 2617 (27.6) |
| stage2 HTN | 2572 (27.1) | 2838 (29.8) | 2879 (30.3) | 2991 (31.4) | 3172 (33.4) |
| **Diabetes** |  |  |  |  |  |
| NO | 8951 (94.3) | 8922 (93.8) | 8879 (93.5) | 8731 (91.9) | 8663 (91.2) |
| YES | 540 (5.7) | 591 (6.2) | 618 (6.5) | 774 (8.1) | 832 (8.8) |
| **Dyslipidemia** |  |  |  |  |  |
| NO | 6203 (65.4) | 6458 (67.9) | 6177 (65.0) | 6231 (65.5) | 6362 (67.0) |
| Yes | 3288 (34.6) | 3055 (32.1) | 3320 (35.0) | 3274 (34.5) | 3133 (33.0) |
| **CVD** |  |  |  |  |  |
| NO | 9195 (96.9) | 9169 (96.4) | 9070 (95.5) | 9024 (94.9) | 9066 (95.5) |
| YES | 296 (3.1) | 344 (3.6) | 427 (4.5) | 481 (5.1) | 429 (4.5) |

Abbreviation:PM_2.5_, fine particulate matter; SBP, systolic blood pressure; DBP, diastolic blood pressure; DASH, dietary approaches to stop hypertension; AMED, alternative Mediterranean; MUFA: SFA, the ratio of monounsaturated to saturated fatty acids; BMI, body mass index; HTN, hypertension; CVD, cardiovascular disease.

# Table S5 Association between DASH and AMED scores with HTN, stage 1 HTN, and stage 2 HTN.

| **Dietary patterns** | **HTN** | |  | **Stage 1 HTN** | |  | **Stage 2 HTN** | |
| --- | --- | --- | --- | --- | --- | --- | --- | --- |
|  | **OR (95%CI)** | ***P*-trend** |  | **OR (95%CI)** | ***P*-trend** |  | **OR (95%CI)** | ***P*-trend** |
| **DASH Score** |  | <0.001 |  |  | <0.001 |  |  | <0.001 |
| Q1 | Reference |  |  | Reference |  |  | Reference |  |
| Q2 | 0.94 (0.88, 1.00) |  |  | 0.95 (0.88, 1.03) |  |  | 0.93 (0.85, 1.00) |  |
| Q3 | 0.92 (0.86, 0.97) |  |  | 0.91 (0.85, 0.98) |  |  | 0.92 (0.86, 0.99) |  |
| Q4 | 0.84 (0.79, 0.90) |  |  | 0.86 (0.80, 0.93) |  |  | 0.80 (0.74, 0.87) |  |
| Q5 | 0.78 (0.73, 0.83) |  |  | 0.82 (0.75, 0.89) |  |  | 0.72 (0.66, 0.78) |  |
| **AMED Score** |  | 0.003 |  |  | 0.048 |  |  | 0.003 |
| Q1 | Reference |  |  | Reference |  |  | Reference |  |
| Q2 | 1.01 (0.95, 1.07) |  |  | 0.96 (0.90, 1.04) |  |  | 1.05 (0.97, 1.13) |  |
| Q3 | 0.95 (0.88, 1.01) |  |  | 0.92 (0.85, 0.99) |  |  | 0.98 (0.90, 1.06) |  |
| Q4 | 0.98 (0.91, 1.05) |  |  | 0.99 (0.91, 1.07) |  |  | 0.96 (0.88, 1.05) |  |
| Q5 | 0.91 (0.85, 0.97) |  |  | 0.91 (0.84, 0.98) |  |  | 0.91 (0.84, 0.99) |  |

Abbreviations: DASH, dietary approach to stop hypertension; AMED, alternative Mediterranean; HTN, hypertension; OR, odds ratios; CI, confidence interval.

# Table S6 ORs associated with 25% score increment in DASH score and after alternate subtraction of each of its dietary component.

| **Dietary score** | **Odds ratio (95% CI) †** | ***P* value** | **Reduction in**  **apparent effect (%) ‡** |
| --- | --- | --- | --- |
| **Hypertension** |  |  |  |
| DASH overall | 0.919 (0.900 to 0.938) | <0.001 | 0 |
| DASH minus fruit | 0.946 (0.927 to 0.965) | <0.001 | 33.3 |
| DASH minus vegetables | 0.930 (0.911 to 0.950) | <0.001 | 13.6 |
| DASH minus dairy products | 0.975 (0.956 to 0.995) | 0.005 | 69.1 |
| DASH minus whole grain | 0.933 (0.914 to 0.952) | <0.001 | 17.3 |
| DASH minus sodium | 0.938 (0.919 to 0.958) | <0.001 | 23.5 |
| DASH minus legumes and nuts | 0.944 (0.924 to 0.963) | <0.001 | 30.9 |
| DASH minus red and processed meats | 0.926 (0.907 to 0.946) | <0.001 | 8.6 |
| **Stage 1 hypertension** |  |  |  |
| DASH overall | 0.934 (0.912 to 0.956) | <0.001 | 0 |
| DASH minus fruit | 0.952 (0.930 to 0.975) | <0.001 | 27.3 |
| DASH minus vegetables | 0.939 (0.916 to 0.963) | <0.001 | 7.6 |
| DASH minus dairy products | 0.980 (0.958 to 1.004) | 0.056 | 69.7 |
| DASH minus whole grain | 0.953 (0.930 to 0.976) | <0.001 | 28.8 |
| DASH minus sodium | 0.949 (0.926 to 0.972) | <0.001 | 22.7 |
| DASH minus legumes and nuts | 0.953 (0.931 to 0.977) | <0.001 | 28.8 |
| DASH minus red and processed meats | 0.941 (0.919 to 0.964) | <0.001 | 10.6 |
| **Stage 2 hypertension** |  |  |  |
| DASH overall | 0.896 (0.873 to 0.919) | <0.001 | 0 |
| DASH minus fruit | 0.933 (0.910 to 0.957) | <0.001 | 35.6 |
| DASH minus vegetables | 0.912 (0.888 to 0.936) | <0.001 | 15.4 |
| DASH minus dairy products | 0.967 (0.943 to 0.992) | 0.003 | 68.3 |
| DASH minus whole grain | 0.903 (0.881 to 0.926) | <0.001 | 6.7 |
| DASH minus sodium | 0.919 (0.895 to 0.942) | <0.001 | 22.1 |
| DASH minus legumes and nuts | 0.926 (0.903 to 0.950) | <0.001 | 28.8 |
| DASH minus red and processed meats | 0.903 (0.880 to 0.926) | <0.001 | 6.7 |

Abbreviation: DASH, dietary approaches to stop hypertension; CI, confidence interval.

†, For each DASH score after alternate subtraction of each of its dietary components, the originally estimated coefficients of logistic regression were multiplied by 25/29 and then exponentiated to correct for correct for 35-point scale to preserve comparability. The models were adjusted for age, sex, education, marital status, annual household income, smoking status, passive smoking, alcohol status, physical activity, outdoor time, BMI, household solid fuel use, hypertension family history, setting, region, and the corresponding subtracted component.

‡, Reduction in apparent effect (%) = (OR_DASH overall_ - OR_DASH minus component_)/ (OR_DASH overall_ - 1) *100%, which represents the reduction in the beneficial effect of subtracting certain dietary components from the DASH score.

# Table S7 ORs associated with 25% score increment in AMED score and after alternate subtraction of each of its dietary components.

| **Dietary score** | **Odds ratio (95% CI)** † | ***P* value** | **Reduction in**  **apparent effect (%)** ‡ |
| --- | --- | --- | --- |
| **Hypertension** |  |  |  |
| AMED overall | 0.966 (0.947 to 0.985) | 0.001 | 0 |
| AMED minus fruit | 0.986 (0.967 to 1.005) | 0.092 | 58.8 |
| AMED minus vegetables | 0.973 (0.954 to 0.992) | 0.002 | 20.6 |
| AMED minus alcohol | 0.979 (0.960 to 0.999) | 0.019 | 38.2 |
| AMED minus whole grain | 0.971 (0.951 to 0.992) | 0.002 | 14.7 |
| AMED minus fish | 0.985 (0.966 to 1.004) | 0.073 | 55.9 |
| AMED minus legumes and nuts | 0.991 (0.971 to 1.011) | 0.305 | 73.5 |
| AMED red and processed meats | 0.961 (0.942 to 0.981) | <0.001 | -14.7 |
| AMED minus MUFA: SFA | 0.957 (0.938 to 0.977) | <0.001 | -26.5 |
| **Stage 1 hypertension** |  |  |  |
| AMED overall | 0.974 (0.952 to 0.997) | 0.025 | 0 |
| AMED minus fruit | 0.989 (0.968 to 1.011) | 0.277 | 57.7 |
| AMED minus vegetables | 0.977 (0.956 to 1.000) | 0.025 | 11.5 |
| AMED minus alcohol | 0.984 (0.961 to 1.007) | 0.123 | 38.5 |
| AMED minus whole grain | 0.985 (0.961 to 1.01) | 0.171 | 42.3 |
| AMED minus fish | 0.984 (0.963 to 1.006) | 0.114 | 38.5 |
| AMED minus legumes and nuts | 0.995 (0.972 to 1.018) | 0.613 | 80.8 |
| AMED red and processed meats | 0.972 (0.95 to 0.996) | 0.008 | -7.7 |
| AMED minus MUFA: SFA | 0.968 (0.945 to 0.991) | 0.002 | -23.1 |
| **Stage 2 hypertension** |  |  |  |
| AMED overall | 0.957 (0.934 to 0.981) | 0.001 | 0 |
| AMED minus fruit | 0.985 (0.962 to 1.008) | 0.14 | 65.1 |
| AMED minus vegetables | 0.968 (0.945 to 0.992) | 0.003 | 25.6 |
| AMED minus alcohol | 0.975 (0.952 to 1.000) | 0.025 | 41.9 |
| AMED minus whole grain | 0.958 (0.933 to 0.983) | <0.001 | 2.3 |
| AMED minus fish | 0.985 (0.963 to 1.009) | 0.163 | 65.1 |
| AMED minus legumes and nuts | 0.989 (0.965 to 1.014) | 0.321 | 74.4 |
| AMED red and processed meats | 0.951 (0.927 to 0.975) | <0.001 | -14 |
| AMED minus MUFA: SFA | 0.945 (0.921 to 0.969) | <0.001 | -27.9 |

Abbreviation: AMED, alternative Mediterranean; CI, confidence interval, MUFA: SFA, the ratio of monounsaturated to saturated lipids. †, For each AMED score after alternate subtraction of each of its dietary components, the originally estimated coefficients of logistic regression were multiplied by 29/33 and then exponentiated to correct for correct for 40-point scale to preserve comparability. The models were adjusted for age, sex, education, marital status, annual household income, smoking status, passive smoking, alcohol status, physical activity, outdoor time, BMI, household solid fuel use, hypertension family history, setting, region, and the corresponding subtracted component.

‡, Reduction in apparent effect (%) = (OR_AMED overall_ - OR_AMED minus component_)/ (OR_AMED overall_ - 1) *100%, which represents the reduction in the beneficial effect of subtracting certain dietary components from the AMED score.

# Table S8 ORs and 95% CI associated with per IQR increase in PM_2.5_ and its constituents by quintiles of DASH score.

| **DASH**  **Score** | **Hypertension** | |  | **Stage 1 hypertension** | |  | **Stage 2 hypertension** | |
| --- | --- | --- | --- | --- | --- | --- | --- | --- |
|  | **OR (95CI%)** | ***P*-int** |  | **OR (95CI%)** | ***P*-int** |  | **OR (95CI%)** | ***P*-int** |
| **PM_2.5_** |  | <0.01 |  |  | <0.01 |  |  | <0.01 |
| Q1 | 1.20 (1.10, 1.30) |  |  | 1.17 (1.06, 1.28) |  |  | 1.20 (1.08, 1.32) |  |
| Q2 | 1.22 (1.11, 1.33) |  |  | 1.17 (1.06, 1.30) |  |  | 1.27 (1.14, 1.41) |  |
| Q3 | 1.17 (1.09, 1.25) |  |  | 1.15 (1.06, 1.24) |  |  | 1.17 (1.08, 1.27) |  |
| Q4 | 1.07 (1.00, 1.15) |  |  | 1.03 (0.96, 1.12) |  |  | 1.10 (1.01, 1.20) |  |
| Q5 | 0.98 (0.91, 1.05) |  |  | 0.98 (0.90, 1.07) |  |  | 0.97 (0.88, 1.06) |  |
| **SO2- 4** |  | <0.01 |  |  | <0.01 |  |  | <0.01 |
| Q1 | 1.27 (1.17, 1.38) |  |  | 1.23 (1.12, 1.36) |  |  | 1.28 (1.16, 1.41) |  |
| Q2 | 1.24 (1.13, 1.36) |  |  | 1.17 (1.05, 1.30) |  |  | 1.31 (1.17, 1.47) |  |
| Q3 | 1.18 (1.10, 1.27) |  |  | 1.16 (1.07, 1.26) |  |  | 1.18 (1.08, 1.29) |  |
| Q4 | 1.11 (1.03, 1.19) |  |  | 1.07 (0.98, 1.17) |  |  | 1.14 (1.04, 1.25) |  |
| Q5 | 1.00 (0.93, 1.09) |  |  | 1.01 (0.92, 1.10) |  |  | 0.99 (0.90, 1.10) |  |
| **NO- 3** |  | <0.01 |  |  | 0.01 |  |  | <0.01 |
| Q1 | 1.30 (1.20, 1.42) |  |  | 1.24 (1.13, 1.36) |  |  | 1.33 (1.20, 1.47) |  |
| Q2 | 1.28 (1.17, 1.41) |  |  | 1.20 (1.09, 1.34) |  |  | 1.36 (1.22, 1.52) |  |
| Q3 | 1.24 (1.16, 1.33) |  |  | 1.21 (1.12, 1.32) |  |  | 1.25 (1.14, 1.36) |  |
| Q4 | 1.15 (1.07, 1.24) |  |  | 1.10 (1.01, 1.19) |  |  | 1.19 (1.09, 1.30) |  |
| Q5 | 1.06 (0.99, 1.15) |  |  | 1.06 (0.97, 1.16) |  |  | 1.06 (0.96, 1.17) |  |
| **NH+ 4** |  | <0.01 |  |  | 0.02 |  |  | <0.01 |
| Q1 | 1.30 (1.20, 1.41) |  |  | 1.24 (1.13, 1.37) |  |  | 1.34 (1.21, 1.47) |  |
| Q2 | 1.29 (1.17, 1.41) |  |  | 1.21 (1.09, 1.34) |  |  | 1.37 (1.23, 1.53) |  |
| Q3 | 1.24 (1.15, 1.33) |  |  | 1.22 (1.12, 1.32) |  |  | 1.24 (1.14, 1.35) |  |
| Q4 | 1.16 (1.08, 1.25) |  |  | 1.11 (1.02, 1.21) |  |  | 1.20 (1.10, 1.32) |  |
| Q5 | 1.06 (0.98, 1.15) |  |  | 1.06 (0.97, 1.16) |  |  | 1.06 (0.96, 1.17) |  |
| **BC** |  | <0.01 |  |  | <0.01 |  |  | <0.01 |
| Q1 | 1.17 (1.09, 1.26) |  |  | 1.16 (1.06, 1.26) |  |  | 1.16 (1.06, 1.28) |  |
| Q2 | 1.17 (1.08, 1.27) |  |  | 1.13 (1.03, 1.25) |  |  | 1.21 (1.10, 1.34) |  |
| Q3 | 1.12 (1.05, 1.19) |  |  | 1.10 (1.02, 1.18) |  |  | 1.13 (1.04, 1.22) |  |
| Q4 | 1.05 (0.99, 1.12) |  |  | 1.02 (0.95, 1.10) |  |  | 1.08 (1.00, 1.17) |  |
| Q5 | 0.96 (0.90, 1.02) |  |  | 0.95 (0.88, 1.02) |  |  | 0.97 (0.89, 1.05) |  |
| **OM** |  | <0.01 |  |  | <0.01 |  |  | <0.01 |
| Q1 | 1.20 (1.11, 1.30) |  |  | 1.16 (1.06,1.27) |  |  | 1.20 (1.09, 1.32) |  |
| Q2 | 1.21 (1.11, 1.32) |  |  | 1.15 (1.05,1.27) |  |  | 1.26 (1.13, 1.40) |  |
| Q3 | 1.15 (1.08, 1.23) |  |  | 1.12 (1.04,1.20) |  |  | 1.16 (1.07, 1.26) |  |
| Q4 | 1.06 (1.00, 1.13) |  |  | 1.02 (0.95,1.10) |  |  | 1.10 (1.02, 1.19) |  |
| Q5 | 0.97 (0.91, 1.04) |  |  | 0.96 (0.89,1.03) |  |  | 0.99 (0.91, 1.07) |  |

Abbreviation: DASH, dietary approaches to stop hypertension; PM_2.5_, fine particulate matter; SO2- 4, sulfate; NO- 3, nitrate; NH+ 4, ammonium; BC, black carbon; OM, organic matter; IQR, interquartile range; P-int, P value for interaction; Q, quintile; OR, odds ratio.

# Table S9 ORs and 95% CI associated with per IQR increase in PM_2.5_ and its constituents by quintiles of AMED score.

| **AMED**  **Score** | **Hypertension** | |  | **Stage 1 hypertension** | |  | **Stage 2 hypertension** | |
| --- | --- | --- | --- | --- | --- | --- | --- | --- |
|  | **OR (95CI%)** | ***P*-int** |  | **OR (95CI%)** | ***P*-int** |  | **OR (95CI%)** | ***P*-int** |
| **PM_2.5_** |  | 0.03 |  |  | 0.20 |  |  | 0.01 |
| Q1 | 1.19 (1.09, 1.29) |  |  | 1.13 (1.03, 1.25) |  |  | 1.21 (1.09, 1.35) |  |
| Q2 | 1.16 (1.08, 1.24) |  |  | 1.13 (1.04, 1.23) |  |  | 1.17 (1.08, 1.28) |  |
| Q3 | 1.08 (1.00, 1.16) |  |  | 1.07 (0.98, 1.16) |  |  | 1.07 (0.98, 1.18) |  |
| Q4 | 1.03 (0.95, 1.12) |  |  | 1.03 (0.94, 1.13) |  |  | 1.02 (0.93, 1.13) |  |
| Q5 | 1.04 (0.97, 1.11) |  |  | 1.03 (0.95, 1.11) |  |  | 1.04 (0.96, 1.13) |  |
| **SO2- 4** |  | <0.01 |  |  | 0.10 |  |  | <0.01 |
| Q1 | 1.27 (1.17, 1.39) |  |  | 1.20 (1.09, 1.32) |  |  | 1.32 (1.19, 1.46) |  |
| Q2 | 1.20 (1.11, 1.29) |  |  | 1.16 (1.06, 1.27) |  |  | 1.23 (1.12, 1.34) |  |
| Q3 | 1.10 (1.02, 1.19) |  |  | 1.09 (0.99, 1.20) |  |  | 1.10 (1.00, 1.22) |  |
| Q4 | 1.05 (0.96, 1.14) |  |  | 1.04 (0.95, 1.15) |  |  | 1.03 (0.93, 1.14) |  |
| Q5 | 1.06 (0.99, 1.14) |  |  | 1.05 (0.97, 1.14) |  |  | 1.06 (0.97, 1.17) |  |
| **NO- 3** |  | 0.05 |  |  | 0.37 |  |  | <0.01 |
| Q1 | 1.29 (1.18, 1.41) |  |  | 1.21 (1.09, 1.34) |  |  | 1.35 (1.21, 1.50) |  |
| Q2 | 1.23 (1.14, 1.33) |  |  | 1.18 (1.08, 1.29) |  |  | 1.27 (1.16, 1.39) |  |
| Q3 | 1.16 (1.07, 1.26) |  |  | 1.14 (1.04, 1.25) |  |  | 1.17 (1.06, 1.29) |  |
| Q4 | 1.11 (1.03, 1.21) |  |  | 1.10 (1.00, 1.21) |  |  | 1.10 (0.99, 1.22) |  |
| Q5 | 1.12 (1.05, 1.21) |  |  | 1.09 (1.01, 1.19) |  |  | 1.14 (1.05, 1.25) |  |
| **NH+ 4** |  | 0.03 |  |  | 0.31 |  |  | <0.01 |
| Q1 | 1.30 (1.20, 1.42) |  |  | 1.22 (1.11, 1.35) |  |  | 1.37 (1.23, 1.52) |  |
| Q2 | 1.24 (1.15, 1.34) |  |  | 1.19 (1.09, 1.30) |  |  | 1.29 (1.18, 1.41) |  |
| Q3 | 1.16 (1.07, 1.26) |  |  | 1.14 (1.04, 1.26) |  |  | 1.17 (1.06, 1.29) |  |
| Q4 | 1.11 (1.02, 1.21) |  |  | 1.10 (1.00, 1.21) |  |  | 1.09 (0.98, 1.21) |  |
| Q5 | 1.13 (1.05, 1.21) |  |  | 1.10 (1.01, 1.19) |  |  | 1.15 (1.05, 1.25) |  |
| **BC** |  | <0.01 |  |  | 0.04 |  |  | <0.01 |
| Q1 | 1.18 (1.09, 1.27) |  |  | 1.13 (1.03, 1.23) |  |  | 1.21 (1.10, 1.33) |  |
| Q2 | 1.14 (1.06, 1.21) |  |  | 1.12 (1.03, 1.21) |  |  | 1.15 (1.06, 1.24) |  |
| Q3 | 1.04 (0.97, 1.11) |  |  | 1.03 (0.95, 1.12) |  |  | 1.04 (0.95, 1.13) |  |
| Q4 | 1.00 (0.93, 1.08) |  |  | 0.99 (0.91, 1.08) |  |  | 1.01 (0.92, 1.10) |  |
| Q5 | 1.01 (0.95, 1.07) |  |  | 1.00 (0.93, 1.07) |  |  | 1.02 (0.95, 1.10) |  |
| **OM** |  | <0.01 |  |  | 0.03 |  |  | <0.01 |
| Q1 | 1.21 (1.12, 1.31) |  |  | 1.14 (1.05, 1.25) |  |  | 1.25 (1.13, 1.38) |  |
| Q2 | 1.16 (1.08, 1.24) |  |  | 1.12 (1.04, 1.21) |  |  | 1.18 (1.08, 1.28) |  |
| Q3 | 1.06 (0.99, 1.14) |  |  | 1.04 (0.96, 1.13) |  |  | 1.08 (0.98, 1.17) |  |
| Q4 | 1.02 (0.95, 1.10) |  |  | 1.00 (0.93, 1.09) |  |  | 1.03 (0.94, 1.13) |  |
| Q5 | 1.02 (0.96, 1.09) |  |  | 1.00 (0.93, 1.07) |  |  | 1.04 (0.96, 1.12) |  |

Abbreviation: AMED, alternative Mediterranean; PM_2.5_, fine particulate matter; SO2- 4, sulfate; NO- 3, nitrate; NH+ 4, ammonium; BC, black carbon; OM, organic matter; IQR, interquartile range; P-int, P value for interaction; Q, quintile; OR, odds ratio; CI, confidence interval.

# Table S10 OR (95% CI) and *P* values for the interaction between dietary patterns and PM_2.5_ and its components, keeping PM_2.5_ and its components and DASH/AMED scores as continuous variables.

|  | **HTN** |  |  | **Stage 1 HTN** |  |  | **Stage 2 HTN** |  |
| --- | --- | --- | --- | --- | --- | --- | --- | --- |
|  | **OR (95%CI)** | ***P*-int** |  | **OR (95%CI)** | ***P*-int** |  | **OR (95%CI)** | ***P*-int** |
| **DASH score** | | | | | | | | |
| PM_2.5_ | 0.983 (0.976, 0.990) | <0.01 |  | 0.986 (0.979, 0.994) | <0.01 |  | 0.981 (0.972, 0.989) | <0.01 |
| SO2- 4 | 0.981 (0.974, 0.989) | <0.01 |  | 0.985 (0.977, 0.993) | <0.01 |  | 0.978 (0.969, 0.987) | <0.01 |
| NO- 3 | 0.984 (0.977, 0.991) | <0.01 |  | 0.988 (0.980, 0.996) | <0.01 |  | 0.981 (0.972, 0.989) | <0.01 |
| NH+ 4 | 0.984 (0.977, 0.991) | <0.01 |  | 0.988 (0.979, 0.996) | <0.01 |  | 0.980 (0.972, 0.989) | <0.01 |
| BC | 0.984 (0.978, 0.990) | <0.01 |  | 0.985 (0.978, 0.992) | <0.01 |  | 0.984 (0.976, 0.991) | <0.01 |
| OM | 0.982 (0.976, 0.989) | <0.01 |  | 0.984 (0.977, 0.992) | <0.01 |  | 0.982 (0.974, 0.99) | <0.01 |
| **AMED score** | | | | | | | | |
| PM_2.5_ | 0.988 (0.981, 0.996) | <0.01 |  | 0.993 (0.984, 1.001) | **0.10** |  | 0.985 (0.976, 0.994) | <0.01 |
| SO2- 4 | 0.984 (0.977, 0.992) | <0.01 |  | 0.990 (0.981, 0.999) | 0.04 |  | 0.980 (0.970, 0.989) | <0.01 |
| NO- 3 | 0.988 (0.980, 0.996) | <0.01 |  | 0.994 (0.985, 1.003) | **0.20** |  | 0.983 (0.974, 0.993) | <0.01 |
| NH+ 4 | 0.987 (0.980, 0.995) | <0.01 |  | 0.993 (0.984, 1.002) | **0.14** |  | 0.982 (0.973, 0.992) | <0.01 |
| BC | 0.987 (0.980, 0.993) | <0.01 |  | 0.990 (0.982, 0.997) | <0.01 |  | 0.985 (0.977, 0.993) | <0.01 |
| OM | 0.986 (0.979, 0.993) | <0.01 |  | 0.989 (0.981, 0.997) | <0.01 |  | 0.984 (0.976, 0.993) | <0.01 |

Abbreviation: PM_2.5_, fine particulate matter; SO2- 4, sulfate; NO- 3, nitrate; NH+ 4, ammonium; BC, black carbon; OM; IQR, interquartile range; HTN, hypertension; IQR, interquartile range. Note: The models were adjusted for age, sex, education, marital status, annual household income, smoking status, passive smoking, alcohol status, physical activity, outdoor time, BMI, household solid fuel use, hypertension family history, residence, and region and remaining nine dietary components.

# Table S11 ORs and 95% CI of hypertension risk associated with per IQR increase in PM_2.5_ and its constituents by quintiles of dietary components score.

| **Components** | **PM_2.5_** |  |  | **Sulfate** |  |  | **Nitrate** |  |  | **Ammonium** |  |  | **Black carbon** |  |  | **Organic matter** |  |
| --- | --- | --- | --- | --- | --- | --- | --- | --- | --- | --- | --- | --- | --- | --- | --- | --- | --- |
|  | **OR (95CI%)** | ***P*-int** |  | **OR (95CI%)** | ***P*-int** |  | **OR (95CI%)** | ***P*-int** |  | **OR (95CI%)** | ***P*-int** |  | **OR (95CI%)** | ***P*-int** |  | **OR (95CI%)** | ***P*-int** |
| **Fruits** |  | **<0.01** |  |  | **0.02** |  |  | **0.03** |  |  | **0.03** |  |  | **<0.01** |  |  | **<0.01** |
| Q1 | 1.14 (1.05, 1.23) |  |  | 1.16 (1.07, 1.26) |  |  | 1.19 (1.09, 1.29) |  |  | 1.20 (1.11, 1.30) |  |  | 1.13 (1.05, 1.22) |  |  | 1.15 (1.07, 1.24) |  |
| Q2 | 1.17 (1.08, 1.26) |  |  | 1.20 (1.11, 1.30) |  |  | 1.26 (1.16, 1.36) |  |  | 1.26 (1.17, 1.36) |  |  | 1.12 (1.04, 1.2) |  |  | 1.15 (1.07, 1.23) |  |
| Q3 | 1.10 (1.03, 1.19) |  |  | 1.14 (1.05, 1.23) |  |  | 1.18 (1.09, 1.27) |  |  | 1.18 (1.10, 1.28) |  |  | 1.09 (1.02, 1.16) |  |  | 1.11 (1.03, 1.19) |  |
| Q4 | 1.00 (0.93, 1.08) |  |  | 1.03 (0.96, 1.12) |  |  | 1.08 (1.00, 1.17) |  |  | 1.09 (1.01, 1.17) |  |  | 0.97 (0.91, 1.04) |  |  | 0.98 (0.92, 1.05) |  |
| Q5 | 1.02 (0.95, 1.09) |  |  | 1.05 (0.98, 1.14) |  |  | 1.10 (1.02, 1.19) |  |  | 1.10 (1.02, 1.19) |  |  | 1.00 (0.94, 1.06) |  |  | 1.00 (0.94, 1.07) |  |
| **Vegetables** |  | **0.03** |  |  | **0.03** |  |  | 0.06 |  |  | 0.05 |  |  | **0.03** |  |  | **0.02** |
| Q1 | 1.13 (1.05, 1.21) |  |  | 1.18 (1.10, 1.28) |  |  | 1.22 (1.14, 1.32) |  |  | 1.23 (1.14, 1.32) |  |  | 1.10 (1.03, 1.18) |  |  | 1.12 (1.05, 1.20) |  |
| Q2 | 1.14 (1.04, 1.26) |  |  | 1.18 (1.06, 1.31) |  |  | 1.22 (1.10, 1.35) |  |  | 1.23 (1.11, 1.37) |  |  | 1.13 (1.03, 1.24) |  |  | 1.14 (1.04, 1.26) |  |
| Q3 | 1.12 (1.04, 1.2) |  |  | 1.14 (1.05, 1.23) |  |  | 1.18 (1.10, 1.28) |  |  | 1.18 (1.09, 1.27) |  |  | 1.08 (1.01, 1.15) |  |  | 1.09 (1.02, 1.17) |  |
| Q4 | 1.00 (0.94, 1.07) |  |  | 1.03 (0.96, 1.10) |  |  | 1.08 (1.00, 1.15) |  |  | 1.08 (1.01, 1.16) |  |  | 0.99 (0.93, 1.05) |  |  | 1.00 (0.94, 1.06) |  |
| Q5 | 1.05 (0.98, 1.13) |  |  | 1.10 (1.02, 1.19) |  |  | 1.14 (1.06, 1.23) |  |  | 1.15 (1.07, 1.24) |  |  | 1.02 (0.96, 1.10) |  |  | 1.02 (0.96, 1.10) |  |
| **Dairy** |  | **<0.01** |  |  | **<0.01** |  |  | 0.05 |  |  | **0.04** |  |  | **<0.01** |  |  | **<0.01** |
| Q1 | 1.12 (1.07, 1.18) |  |  | 1.16 (1.10, 1.22) |  |  | 1.20 (1.14, 1.26) |  |  | 1.20 (1.14, 1.26) |  |  | 1.09 (1.05, 1.14) |  |  | 1.11 (1.06, 1.16) |  |
| Q4 | 1.04 (0.97, 1.12) |  |  | 1.09 (1.01, 1.18) |  |  | 1.13 (1.05, 1.22) |  |  | 1.14 (1.06, 1.23) |  |  | 1.04 (0.98, 1.11) |  |  | 1.04 (0.97, 1.11) |  |
| Q5 | 1.00 (0.93, 1.07) |  |  | 1.01 (0.93, 1.09) |  |  | 1.08 (0.99, 1.17) |  |  | 1.08 (0.99, 1.17) |  |  | 0.97 (0.91, 1.03) |  |  | 0.99 (0.93, 1.06) |  |
| **Sodium** |  | 0.57 |  |  | 0.47 |  |  | 0.67 |  |  | 0.67 |  |  | 0.32 |  |  | 0.48 |
| Q1 | 1.08 (1.00, 1.16) |  |  | 1.13 (1.04, 1.22) |  |  | 1.16 (1.07, 1.25) |  |  | 1.15 (1.07, 1.25) |  |  | 1.07 (1.00, 1.14) |  |  | 1.06 (0.99, 1.14) |  |
| Q2 | 1.10 (1.03, 1.19) |  |  | 1.13 (1.04, 1.22) |  |  | 1.18 (1.09, 1.27) |  |  | 1.18 (1.09, 1.27) |  |  | 1.08 (1.01, 1.15) |  |  | 1.09 (1.02, 1.17) |  |
| Q3 | 1.04 (0.97, 1.12) |  |  | 1.06 (0.98, 1.14) |  |  | 1.11 (1.03, 1.20) |  |  | 1.12 (1.03, 1.20) |  |  | 1.01 (0.95, 1.08) |  |  | 1.04 (0.97, 1.11) |  |
| Q4 | 1.05 (0.98, 1.13) |  |  | 1.10 (1.02, 1.19) |  |  | 1.16 (1.07, 1.25) |  |  | 1.17 (1.08, 1.26) |  |  | 1.02 (0.95, 1.09) |  |  | 1.03 (0.96, 1.11) |  |
| Q5 | 1.12 (1.04, 1.20) |  |  | 1.16 (1.07, 1.25) |  |  | 1.19 (1.10, 1.29) |  |  | 1.20 (1.11, 1.30) |  |  | 1.09 (1.02, 1.17) |  |  | 1.10 (1.03, 1.18) |  |
| **Legumes and nuts** |  | 0.26 |  |  | 0.09 |  |  | 0.15 |  |  | 0.14 |  |  | 0.13 |  |  | 0.08 |
| Q1 | 1.15 (1.06, 1.24) |  |  | 1.21 (1.11, 1.31) |  |  | 1.24 (1.14, 1.35) |  |  | 1.24 (1.15, 1.35) |  |  | 1.13 (1.05, 1.22) |  |  | 1.16 (1.08, 1.25) |  |
| Q2 | 1.03 (0.95, 1.11) |  |  | 1.05 (0.97, 1.14) |  |  | 1.09 (1.00, 1.18) |  |  | 1.10 (1.02, 1.19) |  |  | 1.01 (0.94, 1.09) |  |  | 1.02 (0.95, 1.09) |  |
| Q3 | 1.09 (1.01, 1.17) |  |  | 1.12 (1.04, 1.22) |  |  | 1.19 (1.10, 1.28) |  |  | 1.19 (1.10, 1.29) |  |  | 1.05 (0.98, 1.12) |  |  | 1.06 (0.99, 1.14) |  |
| Q4 | 1.06 (0.98, 1.13) |  |  | 1.07 (0.99, 1.15) |  |  | 1.12 (1.04, 1.21) |  |  | 1.12 (1.03, 1.20) |  |  | 1.02 (0.96, 1.09) |  |  | 1.04 (0.98, 1.12) |  |
| Q5 | 1.09 (1.01, 1.17) |  |  | 1.13 (1.05, 1.22) |  |  | 1.17 (1.08, 1.26) |  |  | 1.18 (1.09, 1.27) |  |  | 1.06 (1.00, 1.14) |  |  | 1.07 (1.00, 1.14) |  |
| **Red meat** |  | 0.26 |  |  | 0.10 |  |  | 0.27 |  |  | 0.23 |  |  | 0.06 |  |  | 0.13 |
| Q1 | 1.11 (1.03, 1.2) |  |  | 1.18 (1.09, 1.28) |  |  | 1.22 (1.12, 1.32) |  |  | 1.22 (1.13, 1.32) |  |  | 1.10 (1.03, 1.18) |  |  | 1.11 (1.03, 1.20) |  |
| Q2 | 1.07 (0.99, 1.15) |  |  | 1.09 (1.01, 1.18) |  |  | 1.12 (1.04, 1.22) |  |  | 1.12 (1.04, 1.21) |  |  | 1.06 (0.99, 1.13) |  |  | 1.07 (1.00, 1.15) |  |
| Q3 | 1.03 (0.95, 1.10) |  |  | 1.06 (0.98, 1.14) |  |  | 1.12 (1.04, 1.21) |  |  | 1.13 (1.05, 1.22) |  |  | 0.98 (0.92, 1.05) |  |  | 1.00 (0.93, 1.07) |  |
| Q4 | 1.05 (0.98, 1.13) |  |  | 1.07 (0.99, 1.16) |  |  | 1.12 (1.04, 1.21) |  |  | 1.13 (1.05, 1.22) |  |  | 1.03 (0.96, 1.10) |  |  | 1.05 (0.98, 1.12) |  |
| Q5 | 1.13 (1.05, 1.22) |  |  | 1.18 (1.09, 1.28) |  |  | 1.21 (1.12, 1.32) |  |  | 1.22 (1.13, 1.32) |  |  | 1.10 (1.03, 1.18) |  |  | 1.11 (1.03, 1.19) |  |
| **Whole grains** |  | **<0.01** |  |  | **<0.01** |  |  | **<0.01** |  |  | **<0.01** |  |  | **0.02** |  |  | **<0.01** |
| Q1 | 1.18 (1.11, 1.25) |  |  | 1.22 (1.15, 1.30) |  |  | 1.27 (1.19, 1.35) |  |  | 1.28 (1.21, 1.36) |  |  | 1.12 (1.06, 1.18) |  |  | 1.15 (1.09, 1.22) |  |
| Q3 | 1.06 (0.97, 1.16) |  |  | 1.07 (0.98, 1.17) |  |  | 1.14 (1.04, 1.25) |  |  | 1.13 (1.03, 1.23) |  |  | 1.03 (0.95, 1.11) |  |  | 1.05 (0.97, 1.14) |  |
| Q4 | 1.04 (0.97, 1.11) |  |  | 1.07 (0.99, 1.15) |  |  | 1.12 (1.04, 1.21) |  |  | 1.11 (1.03, 1.20) |  |  | 1.02 (0.96, 1.09) |  |  | 1.02 (0.96, 1.09) |  |
| Q5 | 1.00 (0.94, 1.07) |  |  | 1.02 (0.95, 1.10) |  |  | 1.05 (0.98, 1.13) |  |  | 1.06 (0.98, 1.14) |  |  | 1.01 (0.95, 1.07) |  |  | 1.02 (0.96, 1.08) |  |
| **MUFA:SFA** |  | 0.49 |  |  | 0.76 |  |  | 0.60 |  |  | 0.62 |  |  | 0.68 |  |  | 0.49 |
| Q1 | 1.09 (1.02, 1.18) |  |  | 1.12 (1.03, 1.21) |  |  | 1.17 (1.08, 1.27) |  |  | 1.17 (1.08, 1.27) |  |  | 1.05 (0.99, 1.13) |  |  | 1.06 (0.99, 1.14) |  |
| Q2 | 1.05 (0.97, 1.12) |  |  | 1.09 (1.01, 1.18) |  |  | 1.13 (1.05, 1.22) |  |  | 1.13 (1.05, 1.22) |  |  | 1.03 (0.96, 1.10) |  |  | 1.04 (0.97, 1.11) |  |
| Q3 | 1.04 (0.96, 1.12) |  |  | 1.09 (1.01, 1.18) |  |  | 1.12 (1.03, 1.20) |  |  | 1.13 (1.05, 1.22) |  |  | 1.03 (0.97, 1.11) |  |  | 1.04 (0.97, 1.11) |  |
| Q4 | 1.10 (1.02, 1.19) |  |  | 1.11 (1.03, 1.20) |  |  | 1.17 (1.09, 1.27) |  |  | 1.17 (1.08, 1.26) |  |  | 1.06 (0.99, 1.13) |  |  | 1.08 (1.01, 1.16) |  |
| Q5 | 1.11 (1.03, 1.20) |  |  | 1.16 (1.07, 1.26) |  |  | 1.21 (1.11, 1.31) |  |  | 1.22 (1.12, 1.32) |  |  | 1.10 (1.02, 1.17) |  |  | 1.12 (1.04, 1.20) |  |
| **Fish** |  | 0.28 |  |  | 0.79 |  |  | 0.34 |  |  | 0.39 |  |  | 0.75 |  |  | 0.65 |
| Q1 | 1.04 (0.97, 1.12) |  |  | 1.12 (1.04, 1.21) |  |  | 1.14 (1.05, 1.23) |  |  | 1.15 (1.07, 1.24) |  |  | 1.06 (0.99, 1.13) |  |  | 1.06 (0.99, 1.13) |  |
| Q2 | 1.07 (0.99, 1.15) |  |  | 1.10 (1.02, 1.19) |  |  | 1.13 (1.05, 1.22) |  |  | 1.13 (1.05, 1.21) |  |  | 1.06 (1.00, 1.14) |  |  | 1.07 (1.00, 1.15) |  |
| Q3 | 1.07 (1.00, 1.15) |  |  | 1.10 (1.02, 1.19) |  |  | 1.15 (1.06, 1.24) |  |  | 1.15 (1.07, 1.24) |  |  | 1.05 (0.98, 1.12) |  |  | 1.06 (0.99, 1.14) |  |
| Q4 | 1.07 (0.99, 1.15) |  |  | 1.09 (1.01, 1.18) |  |  | 1.15 (1.06, 1.25) |  |  | 1.15 (1.06, 1.25) |  |  | 1.02 (0.95, 1.09) |  |  | 1.03 (0.96, 1.11) |  |
| Q5 | 1.16 (1.07, 1.26) |  |  | 1.16 (1.07, 1.26) |  |  | 1.25 (1.15, 1.36) |  |  | 1.25 (1.15, 1.36) |  |  | 1.08 (1.01, 1.16) |  |  | 1.11 (1.03, 1.20) |  |
| **Alcohol** |  | 0.67 |  |  | 0.62 |  |  | 0.56 |  |  | 0.50 |  |  | 0.73 |  |  | 0.80 |
| Q1 | 0.98 (0.83, 1.15) |  |  | 0.97 (0.82, 1.16) |  |  | 1.01 (0.85, 1.21) |  |  | 1.00 (0.84, 1.19) |  |  | 0.96 (0.82, 1.12) |  |  | 0.98 (0.84, 1.15) |  |
| Q2 | 1.18 (0.77, 1.81) |  |  | 1.29 (0.82, 2.03) |  |  | 1.24 (0.79, 1.95) |  |  | 1.27 (0.81, 1.98) |  |  | 1.20 (0.80, 1.79) |  |  | 1.15 (0.77, 1.71) |  |
| Q3 | 1.07 (1.02, 1.13) |  |  | 1.12 (1.06, 1.18) |  |  | 1.16 (1.10, 1.22) |  |  | 1.17 (1.11, 1.23) |  |  | 1.06 (1.01, 1.10) |  |  | 1.07 (1.02, 1.12) |  |
| Q4 | 1.09 (1.02, 1.16) |  |  | 1.11 (1.04, 1.19) |  |  | 1.16 (1.09, 1.25) |  |  | 1.16 (1.09, 1.25) |  |  | 1.04 (0.98, 1.11) |  |  | 1.06 (0.99, 1.12) |  |
| Q5 | 1.13 (1.01, 1.27) |  |  | 1.13 (1.00, 1.28) |  |  | 1.21 (1.07, 1.36) |  |  | 1.20 (1.07, 1.35) |  |  | 1.07 (0.96, 1.20) |  |  | 1.10 (0.98, 1.22) |  |

Abbreviation: PM_2.5_, fine particulate matter; IQR, interquartile range; P-int, P value for interaction; Q, quintile; OR, odds ratio; CI, confidence interval; MUFA: SFA, the ratio of monounsaturated to saturated lipids.

The first, second and third quintiles of dairy intake are equal to 0, so they are all assigned score of 1.

The first and second quintiles of whole grain intake are equal to 0, so they are all assigned score of 1.

The models were adjusted for age, sex, education, marital status, annual household income, smoking status, passive smoking, alcohol status, physical activity, outdoor time, BMI, household solid fuel use, hypertension family history, residence, and region and remaining nine dietary components.

# Table S12 ORs and 95% CI of stage 1 hypertension risk associated with per IQR increase in PM_2.5_ and its constituents by quintiles of dietary components score.

| **Components** | **PM_2.5_** |  |  | **Sulfate** |  |  | **Nitrate** |  |  | **Ammonium** |  |  | **Black carbon** |  |  | **Organic matter** |  |
| --- | --- | --- | --- | --- | --- | --- | --- | --- | --- | --- | --- | --- | --- | --- | --- | --- | --- |
|  | **OR (95CI%)** | ***P*-int** |  | **OR (95CI%)** | ***P*-int** |  | **OR (95CI%)** | ***P*-int** |  | **OR (95CI%)** | ***P*-int** |  | **OR (95CI%)** | ***P*-int** |  | **OR (95CI%)** | ***P*-int** |
| **Fruits** |  | 0.09 |  |  | 0.20 |  |  | 0.30 |  |  | 0.30 |  |  | **0.03** |  |  | **0.02** |
| Q1 | 1.15 (1.05, 1.26) |  |  | 1.16 (1.06, 1.28) |  |  | 1.19 (1.08, 1.30) |  |  | 1.19 (1.09, 1.31) |  |  | 1.14 (1.04, 1.24) |  |  | 1.15 (1.05, 1.25) |  |
| Q2 | 1.12 (1.02, 1.22) |  |  | 1.15 (1.05, 1.26) |  |  | 1.19 (1.09, 1.30) |  |  | 1.20 (1.09, 1.31) |  |  | 1.08 (0.99, 1.17) |  |  | 1.09 (1.00, 1.18) |  |
| Q3 | 1.08 (1.00, 1.17) |  |  | 1.11 (1.01, 1.21) |  |  | 1.14 (1.05, 1.24) |  |  | 1.15 (1.05, 1.26) |  |  | 1.06 (0.98, 1.15) |  |  | 1.08 (1.00, 1.16) |  |
| Q4 | 0.99 (0.91, 1.08) |  |  | 1.02 (0.93, 1.11) |  |  | 1.06 (0.97, 1.16) |  |  | 1.06 (0.97, 1.16) |  |  | 0.97 (0.89, 1.04) |  |  | 0.97 (0.90, 1.05) |  |
| Q5 | 1.04 (0.96, 1.13) |  |  | 1.08 (0.99, 1.18) |  |  | 1.12 (1.03, 1.22) |  |  | 1.13 (1.03, 1.23) |  |  | 1.01 (0.94, 1.08) |  |  | 1.00 (0.93, 1.08) |  |
| **Vegetables** |  | 0.17 |  |  | 0.11 |  |  | 0.13 |  |  | 0.08 |  |  | 0.21 |  |  | 0.17 |
| Q1 | 1.10 (1.01, 1.19) |  |  | 1.15 (1.05, 1.25) |  |  | 1.17 (1.08, 1.28) |  |  | 1.18 (1.09, 1.29) |  |  | 1.07 (0.99, 1.16) |  |  | 1.08 (1.00, 1.17) |  |
| Q2 | 1.12 (1.00, 1.26) |  |  | 1.14 (1.01, 1.29) |  |  | 1.18 (1.04, 1.33) |  |  | 1.20 (1.06, 1.35) |  |  | 1.11 (1.00, 1.23) |  |  | 1.12 (1.00, 1.24) |  |
| Q3 | 1.10 (1.01, 1.19) |  |  | 1.12 (1.03, 1.22) |  |  | 1.16 (1.06, 1.26) |  |  | 1.16 (1.06, 1.27) |  |  | 1.06 (0.98, 1.14) |  |  | 1.06 (0.99, 1.14) |  |
| Q4 | 0.99 (0.92, 1.07) |  |  | 1.01 (0.93, 1.09) |  |  | 1.04 (0.96, 1.13) |  |  | 1.04 (0.96, 1.13) |  |  | 0.98 (0.91, 1.05) |  |  | 0.98 (0.92, 1.05) |  |
| Q5 | 1.08 (1.00, 1.18) |  |  | 1.13 (1.04, 1.24) |  |  | 1.17 (1.07, 1.28) |  |  | 1.19 (1.09, 1.30) |  |  | 1.04 (0.96, 1.12) |  |  | 1.03 (0.96, 1.12) |  |
| **Dairy** |  | 0.14 |  |  | 0.10 |  |  | 0.26 |  |  | 0.28 |  |  | 0.05 |  |  | 0.06 |
| Q1 | 1.10 (1.04, 1.17) |  |  | 1.14 (1.07, 1.21) |  |  | 1.16 (1.10, 1.23) |  |  | 1.17 (1.10, 1.24) |  |  | 1.08 (1.02, 1.14) |  |  | 1.08 (1.03, 1.14) |  |
| Q4 | 1.04 (0.95, 1.13) |  |  | 1.08 (0.99, 1.18) |  |  | 1.11 (1.02, 1.21) |  |  | 1.12 (1.03, 1.23) |  |  | 1.03 (0.95, 1.11) |  |  | 1.01 (0.94, 1.10) |  |
| Q5 | 1.01 (0.93, 1.10) |  |  | 1.02 (0.93, 1.12) |  |  | 1.08 (0.98, 1.18) |  |  | 1.08 (0.98, 1.19) |  |  | 0.97 (0.90, 1.05) |  |  | 0.99 (0.92, 1.06) |  |
| **Sodium** |  | 0.95 |  |  | 0.94 |  |  | 0.99 |  |  | 0.99 |  |  | 0.77 |  |  | 0.88 |
| Q1 | 1.08 (0.99, 1.18) |  |  | 1.12 (1.02, 1.23) |  |  | 1.14 (1.04, 1.25) |  |  | 1.13 (1.03, 1.25) |  |  | 1.06 (0.98, 1.15) |  |  | 1.05 (0.97, 1.14) |  |
| Q2 | 1.09 (1.00, 1.19) |  |  | 1.11 (1.02, 1.22) |  |  | 1.15 (1.05, 1.26) |  |  | 1.16 (1.06, 1.27) |  |  | 1.07 (0.99, 1.16) |  |  | 1.07 (0.99, 1.16) |  |
| Q3 | 1.05 (0.97, 1.14) |  |  | 1.07 (0.98, 1.17) |  |  | 1.12 (1.03, 1.22) |  |  | 1.13 (1.03, 1.23) |  |  | 1.02 (0.94, 1.10) |  |  | 1.03 (0.96, 1.11) |  |
| Q4 | 1.05 (0.97, 1.14) |  |  | 1.09 (1.00, 1.20) |  |  | 1.14 (1.04, 1.24) |  |  | 1.15 (1.05, 1.26) |  |  | 1.01 (0.94, 1.10) |  |  | 1.02 (0.94, 1.10) |  |
| Q5 | 1.07 (0.99, 1.16) |  |  | 1.10 (1.00, 1.20) |  |  | 1.13 (1.04, 1.24) |  |  | 1.14 (1.04, 1.25) |  |  | 1.04 (0.97, 1.13) |  |  | 1.05 (0.97, 1.14) |  |
| **legumes and nuts** |  | 0.35 |  |  | 0.37 |  |  | 0.26 |  |  | 0.4 |  |  | 0.26 |  |  | 0.12 |
| Q1 | 1.14 (1.04, 1.25) |  |  | 1.18 (1.07, 1.29) |  |  | 1.21 (1.10, 1.33) |  |  | 1.20 (1.09, 1.32) |  |  | 1.12 (1.03, 1.22) |  |  | 1.14 (1.05, 1.24) |  |
| Q2 | 1.01 (0.92, 1.10) |  |  | 1.04 (0.95, 1.15) |  |  | 1.05 (0.96, 1.16) |  |  | 1.07 (0.98, 1.18) |  |  | 1.01 (0.93, 1.10) |  |  | 0.99 (0.92, 1.08) |  |
| Q3 | 1.06 (0.97, 1.15) |  |  | 1.09 (1.00, 1.19) |  |  | 1.15 (1.05, 1.26) |  |  | 1.16 (1.06, 1.27) |  |  | 1.01 (0.94, 1.10) |  |  | 1.02 (0.95, 1.11) |  |
| Q4 | 1.06 (0.98, 1.15) |  |  | 1.07 (0.98, 1.17) |  |  | 1.12 (1.03, 1.22) |  |  | 1.12 (1.02, 1.22) |  |  | 1.02 (0.94, 1.10) |  |  | 1.03 (0.95, 1.11) |  |
| Q5 | 1.08 (1.00, 1.17) |  |  | 1.12 (1.03, 1.22) |  |  | 1.16 (1.06, 1.26) |  |  | 1.17 (1.07, 1.27) |  |  | 1.06 (0.98, 1.14) |  |  | 1.05 (0.98, 1.13) |  |
| **Red meat** |  | 0.49 |  |  | 0.23 |  |  | 0.46 |  |  | 0.45 |  |  | 0.05 |  |  | 0.14 |
| Q1 | 1.11 (1.02, 1.21) |  |  | 1.17 (1.07, 1.28) |  |  | 1.20 (1.09, 1.31) |  |  | 1.21 (1.10, 1.33) |  |  | 1.11 (1.02, 1.20) |  |  | 1.10 (1.02, 1.20) |  |
| Q2 | 1.08 (1.00, 1.18) |  |  | 1.10 (1.01, 1.20) |  |  | 1.11 (1.02, 1.21) |  |  | 1.11 (1.02, 1.22) |  |  | 1.08 (1.00, 1.17) |  |  | 1.08 (1.00, 1.17) |  |
| Q3 | 1.02 (0.94, 1.11) |  |  | 1.04 (0.95, 1.14) |  |  | 1.11 (1.02, 1.21) |  |  | 1.12 (1.02, 1.22) |  |  | 0.95 (0.88, 1.03) |  |  | 0.97 (0.90, 1.05) |  |
| Q4 | 1.04 (0.96, 1.13) |  |  | 1.05 (0.96, 1.15) |  |  | 1.09 (1.00, 1.19) |  |  | 1.10 (1.01, 1.21) |  |  | 1.02 (0.94, 1.10) |  |  | 1.03 (0.95, 1.11) |  |
| Q5 | 1.10 (1.01, 1.20) |  |  | 1.14 (1.04, 1.26) |  |  | 1.18 (1.07, 1.29) |  |  | 1.18 (1.07, 1.30) |  |  | 1.06 (0.97, 1.15) |  |  | 1.06 (0.98, 1.14) |  |
| **Whole grains** |  | **0.03** |  |  | **0.03** |  |  | **0.03** |  |  | **0.01** |  |  | 0.18 |  |  | 0.07 |
| Q1 | 1.15 (1.07, 1.23) |  |  | 1.18 (1.10, 1.27) |  |  | 1.22 (1.14, 1.31) |  |  | 1.24 (1.15, 1.33) |  |  | 1.10 (1.03, 1.17) |  |  | 1.12 (1.05, 1.19) |  |
| Q3 | 1.06 (0.96, 1.17) |  |  | 1.08 (0.97, 1.19) |  |  | 1.14 (1.02, 1.26) |  |  | 1.13 (1.02, 1.25) |  |  | 1.02 (0.93, 1.12) |  |  | 1.04 (0.94, 1.14) |  |
| Q4 | 1.04 (0.96, 1.13) |  |  | 1.07 (0.98, 1.16) |  |  | 1.10 (1.01, 1.20) |  |  | 1.10 (1.01, 1.20) |  |  | 1.02 (0.95, 1.10) |  |  | 1.01 (0.94, 1.09) |  |
| Q5 | 1.00 (0.92, 1.08) |  |  | 1.02 (0.94, 1.11) |  |  | 1.05 (0.96, 1.14) |  |  | 1.05 (0.96, 1.14) |  |  | 1.00 (0.93, 1.07) |  |  | 1.00 (0.93, 1.07) |  |
| **MUFA: SFA** |  | 0.79 |  |  | 0.71 |  |  | 0.75 |  |  | 0.72 |  |  | 0.74 |  |  | 0.62 |
| Q1 | 1.08 (1.00, 1.18) |  |  | 1.09 (1.00, 1.20) |  |  | 1.14 (1.04, 1.25) |  |  | 1.14 (1.04, 1.25) |  |  | 1.04 (0.96, 1.12) |  |  | 1.04 (0.96, 1.12) |  |
| Q2 | 1.07 (0.99, 1.16) |  |  | 1.11 (1.02, 1.22) |  |  | 1.14 (1.05, 1.25) |  |  | 1.15 (1.05, 1.26) |  |  | 1.05 (0.97, 1.13) |  |  | 1.05 (0.97, 1.13) |  |
| Q3 | 1.03 (0.94, 1.12) |  |  | 1.08 (0.99, 1.18) |  |  | 1.10 (1.01, 1.20) |  |  | 1.12 (1.02, 1.22) |  |  | 1.02 (0.94, 1.1) |  |  | 1.01 (0.93, 1.09) |  |
| Q4 | 1.06 (0.98, 1.16) |  |  | 1.06 (0.97, 1.17) |  |  | 1.11 (1.02, 1.22) |  |  | 1.11 (1.01, 1.21) |  |  | 1.02 (0.95, 1.11) |  |  | 1.03 (0.96, 1.12) |  |
| Q5 | 1.1 (1.01, 1.20) |  |  | 1.16 (1.05, 1.27) |  |  | 1.19 (1.08, 1.31) |  |  | 1.20 (1.09, 1.33) |  |  | 1.09 (1.00, 1.18) |  |  | 1.10 (1.01, 1.19) |  |
| **Fish** |  | 0.06 |  |  | 0.30 |  |  | 0.11 |  |  | 0.15 |  |  | 0.34 |  |  | 0.29 |
| Q1 | 1.02 (0.93, 1.11) |  |  | 1.08 (0.99, 1.18) |  |  | 1.09 (1.00, 1.19) |  |  | 1.10 (1.01, 1.21) |  |  | 1.03 (0.95, 1.11) |  |  | 1.02 (0.95, 1.11) |  |
| Q2 | 1.06 (0.98, 1.15) |  |  | 1.09 (1.00, 1.19) |  |  | 1.11 (1.02, 1.21) |  |  | 1.11 (1.01, 1.21) |  |  | 1.06 (0.98, 1.15) |  |  | 1.05 (0.98, 1.14) |  |
| Q3 | 1.02 (0.94, 1.11) |  |  | 1.04 (0.96, 1.14) |  |  | 1.09 (1.00, 1.19) |  |  | 1.10 (1.01, 1.20) |  |  | 0.99 (0.92, 1.07) |  |  | 1.01 (0.93, 1.09) |  |
| Q4 | 1.08 (1.00, 1.18) |  |  | 1.11 (1.01, 1.22) |  |  | 1.16 (1.06, 1.27) |  |  | 1.17 (1.07, 1.28) |  |  | 1.03 (0.96, 1.12) |  |  | 1.04 (0.96, 1.12) |  |
| Q5 | 1.19 (1.09, 1.30) |  |  | 1.19 (1.08, 1.32) |  |  | 1.26 (1.14, 1.38) |  |  | 1.26 (1.15, 1.39) |  |  | 1.11 (1.02, 1.21) |  |  | 1.13 (1.04, 1.23) |  |
| **Alcohol** |  | 0.53 |  |  | 0.66 |  |  | 0.47 |  |  | 0.52 |  |  | 0.89 |  |  | 0.88 |
| Q1 | 0.97 (0.80, 1.17) |  |  | 0.98 (0.80, 1.20) |  |  | 1.00 (0.82, 1.22) |  |  | 1.00 (0.82, 1.22) |  |  | 0.96 (0.81, 1.15) |  |  | 0.96 (0.81, 1.15) |  |
| Q2 | 1.11 (0.67, 1.82) |  |  | 1.22 (0.72, 2.09) |  |  | 1.20 (0.72, 2.03) |  |  | 1.24 (0.73, 2.10) |  |  | 1.06 (0.65, 1.73) |  |  | 1.01 (0.64, 1.60) |  |
| Q3 | 1.05 (1.00, 1.11) |  |  | 1.09 (1.03, 1.15) |  |  | 1.12 (1.06, 1.19) |  |  | 1.13 (1.06, 1.20) |  |  | 1.04 (0.99, 1.09) |  |  | 1.04 (0.99, 1.10) |  |
| Q4 | 1.11 (1.03, 1.20) |  |  | 1.14 (1.05, 1.23) |  |  | 1.18 (1.09, 1.27) |  |  | 1.18 (1.09, 1.28) |  |  | 1.06 (0.99, 1.14) |  |  | 1.06 (0.99, 1.14) |  |
| Q5 | 1.11 (0.98, 1.26) |  |  | 1.10 (0.96, 1.27) |  |  | 1.19 (1.04, 1.36) |  |  | 1.18 (1.03, 1.36) |  |  | 1.04 (0.92, 1.18) |  |  | 1.06 (0.95, 1.20) |  |

Abbreviation: PM_2.5_, fine particulate matter; IQR, interquartile range; P-int, P value for interaction; Q, quintile; OR, odds ratio; CI, confidence interval; MUFA: SFA, the ratio of monounsaturated to saturated lipids.

The first, second and third quintiles of dairy intake are equal to 0, so they are all assigned score of 1.

The first and second quintiles of whole grain intake are equal to 0, so they are all assigned score of 1.

The models were adjusted for age, sex, education, marital status, annual household income, smoking status, passive smoking, alcohol status, physical activity, outdoor time, BMI, household solid fuel use, hypertension family history, residence, and region and remaining nine dietary components.

# Table S13 ORs and 95% CI of stage 2 hypertension risk associated with per IQR increase in PM_2.5_ and its constituents by quintiles of dietary components score.

| **Components** | **PM_2.5_** |  |  | **Sulfate** |  |  | **Nitrate** |  |  | **Ammonium** |  |  | **Black carbon** |  |  | **Organic matter** |  |
| --- | --- | --- | --- | --- | --- | --- | --- | --- | --- | --- | --- | --- | --- | --- | --- | --- | --- |
|  | **OR (95CI%)** | ***P*-int** |  | **OR (95CI%)** | ***P*-int** |  | **OR (95CI%)** | ***P*-int** |  | **OR (95CI%)** | ***P*-int** |  | **OR (95CI%)** | ***P*-int** |  | **OR (95CI%)** | ***P*-int** |
| **Fruits** |  | **<0.01** |  |  | **0.02** |  |  | **0.01** |  |  | **0.01** |  |  | **0.01** |  |  | **<0.01** |
| Q1 | 1.08 (0.98, 1.18) |  |  | 1.11 (1.01, 1.23) |  |  | 1.14 (1.04, 1.25) |  |  | 1.16 (1.05, 1.27) |  |  | 1.10 (1.01, 1.20) |  |  | 1.11 (1.01, 1.21) |  |
| Q2 | 1.20 (1.10, 1.32) |  |  | 1.23 (1.12, 1.36) |  |  | 1.31 (1.20, 1.44) |  |  | 1.32 (1.20, 1.45) |  |  | 1.14 (1.04, 1.24) |  |  | 1.19 (1.09, 1.30) |  |
| Q3 | 1.12 (1.02, 1.22) |  |  | 1.16 (1.06, 1.28) |  |  | 1.20 (1.09, 1.31) |  |  | 1.21 (1.10, 1.33) |  |  | 1.11 (1.02, 1.20) |  |  | 1.13 (1.03, 1.22) |  |
| Q4 | 1.00 (0.91, 1.09) |  |  | 1.03 (0.94, 1.14) |  |  | 1.09 (0.99, 1.20) |  |  | 1.10 (0.99, 1.21) |  |  | 0.98 (0.90, 1.06) |  |  | 1.00 (0.92, 1.08) |  |
| Q5 | 0.99 (0.90, 1.08) |  |  | 1.02 (0.92, 1.12) |  |  | 1.07 (0.97, 1.18) |  |  | 1.07 (0.97, 1.18) |  |  | 0.98 (0.90, 1.06) |  |  | 0.99 (0.92, 1.08) |  |
| **Vegetables** |  | **0.02** |  |  | **0.03** |  |  | 0.06 |  |  | 0.08 |  |  | **0.04** |  |  | **0.02** |
| Q1 | 1.15 (1.06, 1.25) |  |  | 1.21 (1.11, 1.33) |  |  | 1.26 (1.15, 1.38) |  |  | 1.27 (1.16, 1.39) |  |  | 1.12 (1.04, 1.22) |  |  | 1.15 (1.06, 1.24) |  |
| Q2 | 1.14 (1.01, 1.29) |  |  | 1.18 (1.04, 1.35) |  |  | 1.22 (1.08, 1.39) |  |  | 1.24 (1.09, 1.41) |  |  | 1.13 (1.01, 1.27) |  |  | 1.15 (1.02, 1.29) |  |
| Q3 | 1.12 (1.03, 1.23) |  |  | 1.14 (1.04, 1.25) |  |  | 1.19 (1.08, 1.30) |  |  | 1.18 (1.08, 1.30) |  |  | 1.09 (1.00, 1.18) |  |  | 1.12 (1.03, 1.21) |  |
| Q4 | 1.00 (0.92, 1.09) |  |  | 1.03 (0.95, 1.13) |  |  | 1.09 (1.01, 1.19) |  |  | 1.11 (1.01, 1.20) |  |  | 0.99 (0.92, 1.06) |  |  | 1.01 (0.93, 1.09) |  |
| Q5 | 1.00 (0.91, 1.09) |  |  | 1.04 (0.95, 1.15) |  |  | 1.09 (0.99, 1.19) |  |  | 1.10 (1.00, 1.21) |  |  | 1.00 (0.92, 1.08) |  |  | 1.00 (0.91, 1.08) |  |
| **Dairy** |  | **<0.01** |  |  | **<0.01** |  |  | 0.05 |  |  | **0.03** |  |  | **<0.01** |  |  | **0.03** |
| Q1 | 1.12 (1.05, 1.19) |  |  | 1.16 (1.09, 1.24) |  |  | 1.20 (1.13, 1.27) |  |  | 1.21 (1.14, 1.28) |  |  | 1.09 (1.04, 1.16) |  |  | 1.11 (1.05, 1.18) |  |
| Q4 | 1.06 (0.96, 1.16) |  |  | 1.11 (1.01, 1.22) |  |  | 1.15 (1.05, 1.27) |  |  | 1.16 (1.06, 1.28) |  |  | 1.06 (0.98, 1.15) |  |  | 1.07 (0.98, 1.16) |  |
| Q5 | 0.95 (0.87, 1.05) |  |  | 0.96 (0.87, 1.06) |  |  | 1.04 (0.94, 1.16) |  |  | 1.04 (0.93, 1.15) |  |  | 0.95 (0.87, 1.03) |  |  | 0.98 (0.90, 1.07) |  |
| **Sodium** |  | 0.13 |  |  | 0.09 |  |  | 0.19 |  |  | 0.23 |  |  | 0.08 |  |  | 0.13 |
| Q1 | 1.04 (0.95, 1.14) |  |  | 1.11 (1.00, 1.22) |  |  | 1.14 (1.04, 1.26) |  |  | 1.15 (1.04, 1.26) |  |  | 1.04 (0.96, 1.13) |  |  | 1.04 (0.96, 1.13) |  |
| Q2 | 1.11 (1.02, 1.22) |  |  | 1.14 (1.04, 1.25) |  |  | 1.21 (1.10, 1.32) |  |  | 1.20 (1.10, 1.32) |  |  | 1.08 (1.00, 1.17) |  |  | 1.11 (1.02, 1.20) |  |
| Q3 | 1.02 (0.93, 1.12) |  |  | 1.02 (0.93, 1.12) |  |  | 1.08 (0.98, 1.18) |  |  | 1.09 (0.99, 1.20) |  |  | 1.00 (0.92, 1.09) |  |  | 1.03 (0.95, 1.12) |  |
| Q4 | 1.03 (0.94, 1.13) |  |  | 1.08 (0.98, 1.19) |  |  | 1.14 (1.04, 1.26) |  |  | 1.16 (1.05, 1.27) |  |  | 1.00 (0.92, 1.09) |  |  | 1.03 (0.95, 1.12) |  |
| Q5 | 1.16 (1.06, 1.27) |  |  | 1.22 (1.10, 1.34) |  |  | 1.24 (1.13, 1.37) |  |  | 1.26 (1.14, 1.39) |  |  | 1.15 (1.06, 1.25) |  |  | 1.16 (1.07, 1.26) |  |
| **Legumes and nuts** |  | 0.71 |  |  | 0.28 |  |  | 0.47 |  |  | 0.50 |  |  | 0.54 |  |  | 0.57 |
| Q1 | 1.12 (1.02, 1.24) |  |  | 1.20 (1.09, 1.33) |  |  | 1.24 (1.12, 1.37) |  |  | 1.26 (1.14, 1.39) |  |  | 1.11 (1.02, 1.21) |  |  | 1.14 (1.04, 1.25) |  |
| Q2 | 1.04 (0.94, 1.14) |  |  | 1.05 (0.95, 1.16) |  |  | 1.11 (1.01, 1.23) |  |  | 1.12 (1.02, 1.22) |  |  | 1.01 (0.93, 1.10) |  |  | 1.04 (0.95, 1.13) |  |
| Q3 | 1.09 (1.00, 1.20) |  |  | 1.13 (1.03, 1.25) |  |  | 1.19 (1.08, 1.31) |  |  | 1.21 (1.10, 1.33) |  |  | 1.06 (0.98, 1.16) |  |  | 1.07 (0.99, 1.17) |  |
| Q4 | 1.05 (0.96, 1.15) |  |  | 1.06 (0.97, 1.17) |  |  | 1.13 (1.03, 1.24) |  |  | 1.12 (1.02, 1.23) |  |  | 1.03 (0.95, 1.12) |  |  | 1.06 (0.98, 1.15) |  |
| Q5 | 1.07 (0.98, 1.16) |  |  | 1.11 (1.01, 1.22) |  |  | 1.15 (1.05, 1.26) |  |  | 1.16 (1.05, 1.27) |  |  | 1.06 (0.98, 1.15) |  |  | 1.06 (0.98, 1.15) |  |
| **Red meat** |  | 0.46 |  |  | 0.30 |  |  | 0.59 |  |  | 0.50 |  |  | 0.32 |  |  | 0.43 |
| Q1 | 1.09 (0.99, 1.21) |  |  | 1.16 (1.05, 1.28) |  |  | 1.20 (1.09, 1.33) |  |  | 1.21 (1.09, 1.34) |  |  | 1.09 (0.99, 1.19) |  |  | 1.10 (1.00, 1.21) |  |
| Q2 | 1.05 (0.96, 1.16) |  |  | 1.07 (0.97, 1.18) |  |  | 1.13 (1.02, 1.25) |  |  | 1.12 (1.02, 1.23) |  |  | 1.03 (0.95, 1.12) |  |  | 1.06 (0.97, 1.16) |  |
| Q3 | 1.02 (0.93, 1.12) |  |  | 1.06 (0.96, 1.17) |  |  | 1.12 (1.01, 1.23) |  |  | 1.13 (1.03, 1.25) |  |  | 1.01 (0.92, 1.09) |  |  | 1.02 (0.94, 1.11) |  |
| Q4 | 1.06 (0.97, 1.15) |  |  | 1.08 (0.99, 1.19) |  |  | 1.14 (1.04, 1.25) |  |  | 1.15 (1.05, 1.26) |  |  | 1.03 (0.95, 1.12) |  |  | 1.06 (0.97, 1.15) |  |
| Q5 | 1.13 (1.04, 1.24) |  |  | 1.18 (1.07, 1.30) |  |  | 1.22 (1.11, 1.34) |  |  | 1.23 (1.12, 1.36) |  |  | 1.12 (1.03, 1.21) |  |  | 1.13 (1.04, 1.22) |  |
| **Whole grains** |  | **<0.01** |  |  | **<0.01** |  |  | **<0.01** |  |  | **<0.01** |  |  | 0.06 |  |  | **0.02** |
| Q1 | 1.19 (1.10, 1.28) |  |  | 1.24 (1.15, 1.34) |  |  | 1.29 (1.20, 1.39) |  |  | 1.31 (1.22, 1.42) |  |  | 1.13 (1.06, 1.21) |  |  | 1.17 (1.09, 1.26) |  |
| Q3 | 1.05 (0.94, 1.17) |  |  | 1.06 (0.95, 1.19) |  |  | 1.13 (1.01, 1.26) |  |  | 1.11 (1.00, 1.25) |  |  | 1.03 (0.93, 1.13) |  |  | 1.06 (0.96, 1.17) |  |
| Q4 | 1.02 (0.94, 1.12) |  |  | 1.06 (0.96, 1.16) |  |  | 1.12 (1.02, 1.23) |  |  | 1.11 (1.01, 1.22) |  |  | 1.02 (0.94, 1.10) |  |  | 1.03 (0.95, 1.11) |  |
| Q5 | 0.99 (0.91, 1.08) |  |  | 1.01 (0.93, 1.11) |  |  | 1.04 (0.96, 1.14) |  |  | 1.05 (0.96, 1.15) |  |  | 1.01 (0.93, 1.09) |  |  | 1.02 (0.95, 1.10) |  |
| **MUFA: SFA** |  | 0.33 |  |  | 0.67 |  |  | 0.49 |  |  | 0.53 |  |  | 0.53 |  |  | 0.45 |
| Q1 | 1.08 (0.98, 1.18) |  |  | 1.11 (1.01, 1.22) |  |  | 1.16 (1.05, 1.28) |  |  | 1.17 (1.06, 1.28) |  |  | 1.06 (0.97, 1.15) |  |  | 1.07 (0.98, 1.16) |  |
| Q2 | 1.00 (0.92, 1.10) |  |  | 1.06 (0.96, 1.17) |  |  | 1.10 (1.00, 1.22) |  |  | 1.14 (1.04, 1.25) |  |  | 0.99 (0.91, 1.08) |  |  | 1.01 (0.93, 1.10) |  |
| Q3 | 1.05 (0.96, 1.14) |  |  | 1.09 (0.99, 1.20) |  |  | 1.12 (1.02, 1.23) |  |  | 1.20 (1.09, 1.32) |  |  | 1.05 (0.97, 1.14) |  |  | 1.07 (0.98, 1.16) |  |
| Q4 | 1.13 (1.03, 1.24) |  |  | 1.15 (1.04, 1.26) |  |  | 1.22 (1.11, 1.34) |  |  | 1.13 (1.02, 1.25) |  |  | 1.08 (1.00, 1.18) |  |  | 1.11 (1.02, 1.21) |  |
| Q5 | 1.10 (1.01, 1.20) |  |  | 1.15 (1.04, 1.27) |  |  | 1.20 (1.09, 1.31) |  |  | 1.22 (1.1, 1.36) |  |  | 1.09 (1.00, 1.18) |  |  | 1.11 (1.02, 1.21) |  |
| **Fish** |  | 0.66 |  |  | 0.87 |  |  | 0.81 |  |  | 0.79 |  |  | 0.84 |  |  | 0.78 |
| Q1 | 1.03 (0.95, 1.13) |  |  | 1.12 (1.02, 1.24) |  |  | 1.15 (1.04, 1.26) |  |  | 1.17 (1.06, 1.28) |  |  | 1.06 (0.97, 1.15) |  |  | 1.06 (0.98, 1.15) |  |
| Q2 | 1.06 (0.98, 1.16) |  |  | 1.10 (1.01, 1.21) |  |  | 1.14 (1.04, 1.24) |  |  | 1.14 (1.04, 1.25) |  |  | 1.06 (0.98, 1.15) |  |  | 1.08 (0.99, 1.16) |  |
| Q3 | 1.11 (1.01, 1.21) |  |  | 1.14 (1.04, 1.26) |  |  | 1.19 (1.08, 1.30) |  |  | 1.20 (1.09, 1.32) |  |  | 1.08 (1.00, 1.17) |  |  | 1.11 (1.02, 1.20) |  |
| Q4 | 1.05 (0.96, 1.15) |  |  | 1.07 (0.96, 1.18) |  |  | 1.13 (1.03, 1.25) |  |  | 1.13 (1.02, 1.25) |  |  | 1.01 (0.93, 1.10) |  |  | 1.03 (0.95, 1.13) |  |
| Q5 | 1.12 (1.02, 1.24) |  |  | 1.11 (1.00, 1.24) |  |  | 1.21 (1.09, 1.35) |  |  | 1.22 (1.10, 1.36) |  |  | 1.06 (0.96, 1.16) |  |  | 1.10 (1.00, 1.21) |  |
| **Alcohol** |  | 0.50 |  |  | 0.21 |  |  | 0.35 |  |  | 0.23 |  |  | 0.30 |  |  | 0.47 |
| Q1 | 0.97 (0.80, 1.16) |  |  | 0.94 (0.77, 1.15) |  |  | 0.98 (0.80, 1.20) |  |  | 0.96 (0.79, 1.18) |  |  | 0.95 (0.80, 1.13) |  |  | 0.98 (0.82, 1.17) |  |
| Q2 | 1.30 (0.76, 2.21) |  |  | 1.45 (0.82, 2.59) |  |  | 1.32 (0.76, 2.30) |  |  | 1.36 (0.77, 2.37) |  |  | 1.40 (0.84, 2.33) |  |  | 1.32 (0.80, 2.18) |  |
| Q3 | 1.08 (1.02, 1.15) |  |  | 1.14 (1.07, 1.21) |  |  | 1.18 (1.11, 1.26) |  |  | 1.20 (1.13, 1.27) |  |  | 1.07 (1.02, 1.13) |  |  | 1.09 (1.03, 1.15) |  |
| Q4 | 1.04 (0.96, 1.13) |  |  | 1.06 (0.97, 1.16) |  |  | 1.12 (1.03, 1.23) |  |  | 1.13 (1.03, 1.23) |  |  | 1.01 (0.94, 1.09) |  |  | 1.03 (0.96, 1.12) |  |
| Q5 | 1.14 (0.99, 1.31) |  |  | 1.13 (0.98, 1.32) |  |  | 1.20 (1.04, 1.39) |  |  | 1.20 (1.04, 1.39) |  |  | 1.09 (0.95, 1.24) |  |  | 1.11 (0.97, 1.27) |  |

Abbreviation: PM_2.5_, fine particulate matter; IQR, interquartile range; P-int, P value for interaction; Q, quintile; OR, odds ratio; CI, confidence interval; MUFA: SFA, the ratio of monounsaturated to saturated lipids.

The first, second and third quintiles of dairy intake are equal to 0, so they are all assigned score of 1.

The first and second quintiles of whole grain intake are equal to 0, so they are all assigned score of 1.

The models were adjusted for age, sex, education, marital status, annual household income, smoking status, passive smoking, alcohol status, physical activity, outdoor time, BMI, household solid fuel use, hypertension family history, residence, and region and remaining nine dietary components.

# Table S14 ORs and 95% CI of hypertension, stage 1hypertension, and stage 2 hypertension associated with particulate matter by different exposure windows.

| **Pollutants (per IQR, μg/m^3^)** | **Exposure windows** | | |
| --- | --- | --- | --- |
|  | **One years** | **Two years** | **Four years** |
| **Hypertension OR (95%CI)** | | | |
| PM_2.5_ | 1.08 (1.04, 1.12) | 1.07 (1.03, 1.11) | 1.07 (1.03, 1.12) |
| Sulfate | 1.12 (1.08, 1.17) | 1.11 (1.07, 1.16) | 1.11 (1.06, 1.15) |
| Nitrate | 1.15 (1.11, 1.19) | 1.14 (1.10, 1.19) | 1.16 (1.11, 1.21) |
| Ammonium | 1.15 (1.11, 1.20) | 1.15 (1.11, 1.20) | 1.16 (1.12, 1.21) |
| Black carbon | 1.06 (1.03, 1.10) | 1.05 (1.02, 1.09) | 1.05 (1.01, 1.09) |
| Organic matter | 1.07 (1.04, 1.11) | 1.06 (1.03, 1.10) | 1.07 (1.03, 1.11) |
| **Stage 1 hypertension OR (95%CI)** | | | |
| PM_2.5_ | 1.06 (1.01, 1.10) | 1.05 (1.01, 1.10) | 1.06 (1.01, 1.11) |
| Sulfate | 1.09 (1.05, 1.14) | 1.09 (1.04, 1.14) | 1.09 (1.03, 1.14) |
| Nitrate | 1.12 (1.07, 1.17) | 1.11 (1.06, 1.17) | 1.12 (1.07, 1.18) |
| Ammonium | 1.12 (1.07, 1.17) | 1.12 (1.06, 1.17) | 1.13 (1.08, 1.18) |
| Black carbon | 1.04 (1.00, 1.08) | 1.03 (0.99, 1.07) | 1.04 (0.99, 1.08) |
| Organic matter | 1.04 (1.00, 1.08) | 1.04 (0.99, 1.08) | 1.04 (1.00, 1.09) |
| **Stage 2 hypertension OR (95%CI)** | | | |
| PM_2.5_ | 1.09 (1.04, 1.14) | 1.08 (1.03, 1.13) | 1.08 (1.03, 1.13) |
| Sulfate | 1.14 (1.09, 1.19) | 1.13 (1.07, 1.18) | 1.11 (1.06, 1.17) |
| Nitrate | 1.17 (1.11, 1.23) | 1.16 (1.11, 1.22) | 1.17 (1.12, 1.24) |
| Ammonium | 1.17 (1.12, 1.22) | 1.17 (1.12, 1.23) | 1.18 (1.12, 1.24) |
| Black carbon | 1.08 (1.04, 1.13) | 1.06 (1.02, 1.11) | 1.06 (1.01, 1.10) |
| Organic matter | 1.09 (1.05, 1.14) | 1.08 (1.04, 1.13) | 1.08 (1.04, 1.13) |

Abbreviation: PM_2.5_, fine particulate matter; IQR, interquartile range; OR, odds ratio; CI, confidence interval.

The models were adjusted for age, sex, education, marital status, annual household income, smoking status, passive smoking, alcohol status, physical activity, outdoor time, BMI, household solid-fuel use, hypertension family history, residence, and region.

# Table S15 ORs and 95% CI for HTN, stage 1 HTN, and stage 2 HTN associated with a IQR increase in PM_2.5_ and its constituents, stratified by age.

|  | **HTN** | **Stage 1 HTN** | **Stage 2 HTN** |
| --- | --- | --- | --- |
| **PM_2.5_** |  |  |  |
| <65 years | 1.09 (1.04, 1.13) | 1.08 (1.03, 1.13) | 1.10 (1.04, 1.15) |
| ≥65 years | 0.98 (0.87, 1.09) | 0.93 (0.81, 1.06) | 1.00 (0.89, 1.13) |
| **SO2- 4** |  |  |  |
| <65 years | 1.12 (1.08, 1.17) | 1.11 (1.05, 1.17) | 1.14 (1.08, 1.20) |
| ≥65 years | 1.02 (0.91, 1.15) | 0.96 (0.83, 1.11) | 1.05 (0.93, 1.19) |
| **NO- 3** |  |  |  |
| <65 years | 1.17 (1.12, 1.22) | 1.15 (1.09, 1.20) | 1.19 (1.13, 1.26) |
| ≥65 years | 1.05 (0.93, 1.18) | 0.97 (0.84, 1.12) | 1.09 (0.96, 1.23) |
| **NH+ 4** |  |  |  |
| <65 years | 1.18 (1.13, 1.23) | 1.15 (1.10, 1.21) | 1.20 (1.14, 1.27) |
| ≥65 years | 1.07 (0.91, 1.12) | 0.98 (0.85, 1.13) | 1.11 (0.98, 1.25) |
| **BC** |  |  |  |
| <65 years | 1.06 (1.02, 1.10) | 1.05 (1.00, 1.09) | 1.07 (1.02, 1.12) |
| ≥65 years | 1.01 (0.91, 1.12) | 0.96 (0.85, 1.09) | 1.03 (0.92, 1.15) |
| **OM** |  |  |  |
| <65 years | 1.07 (1.03, 1.11) | 1.05 (1.01, 1.10) | 1.09 (1.04, 1.14) |
| ≥65 years | 1.04 (0.93, 1.15) | 0.96 (0.85, 1.09) | 1.07 (0.96, 1.20) |

Abbreviation: PM_2.5_, fine particulate matter; SO2- 4, sulfate; NO- 3, nitrate; NH+ 4, ammonium; BC, black carbon; OM; IQR, interquartile range; HTN, hypertension; IQR, interquartile range.

The models were adjusted for age, sex, education, marital status, annual household income, smoking status, passive smoking, alcohol status, physical activity, outdoor time, BMI, household solid fuel use, HTN family history, residence, and region.

**
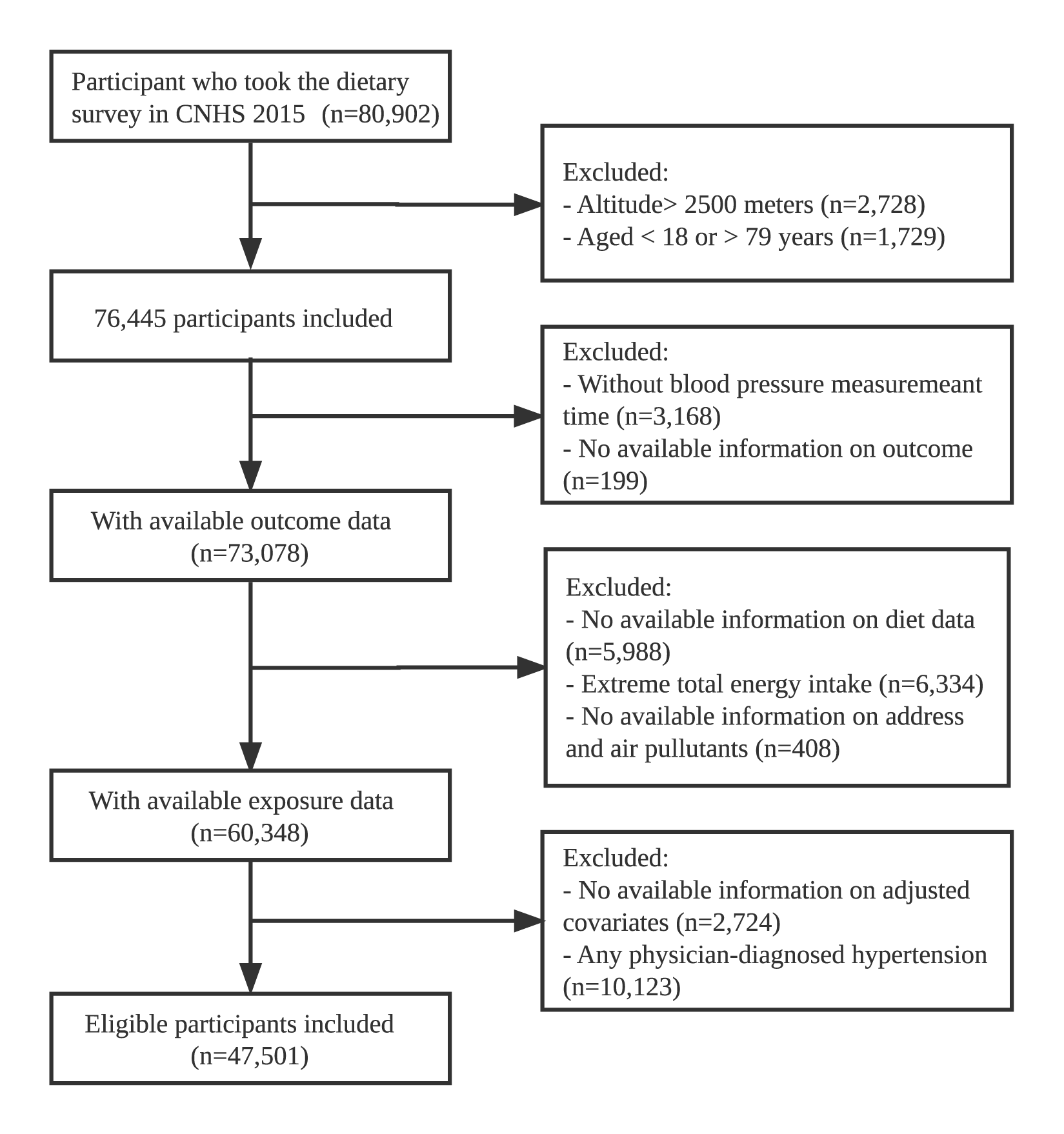
**

# Fig. S1 Flowchart of the study population.


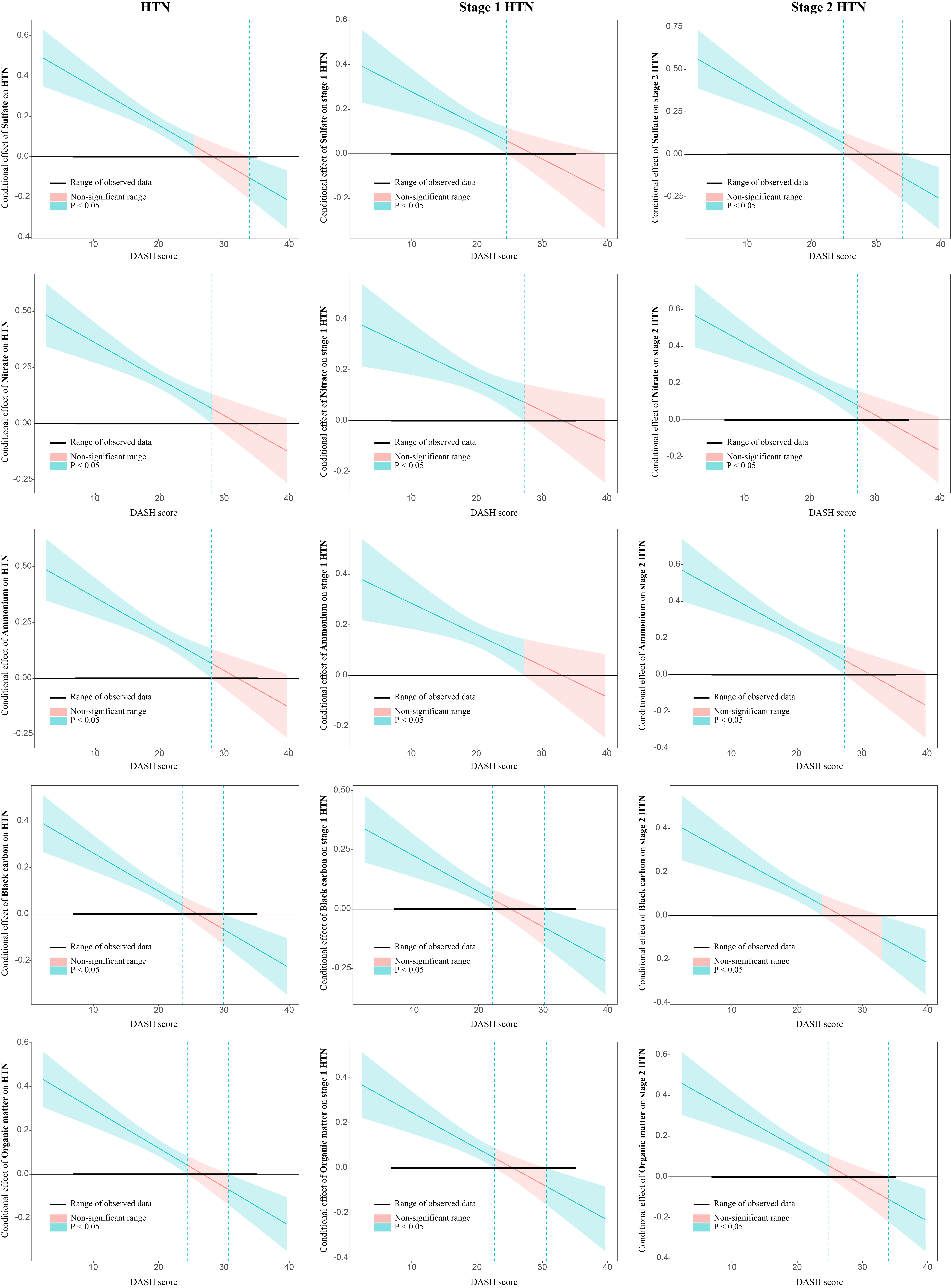


# Fig. S2 The Johnson-Neyman plots of the modifying effects of DASH scores on the relationship between long-term exposure to PM_2.5_ constituents and risk of HTN, stage 1 HTN, and stage 2 HTN.


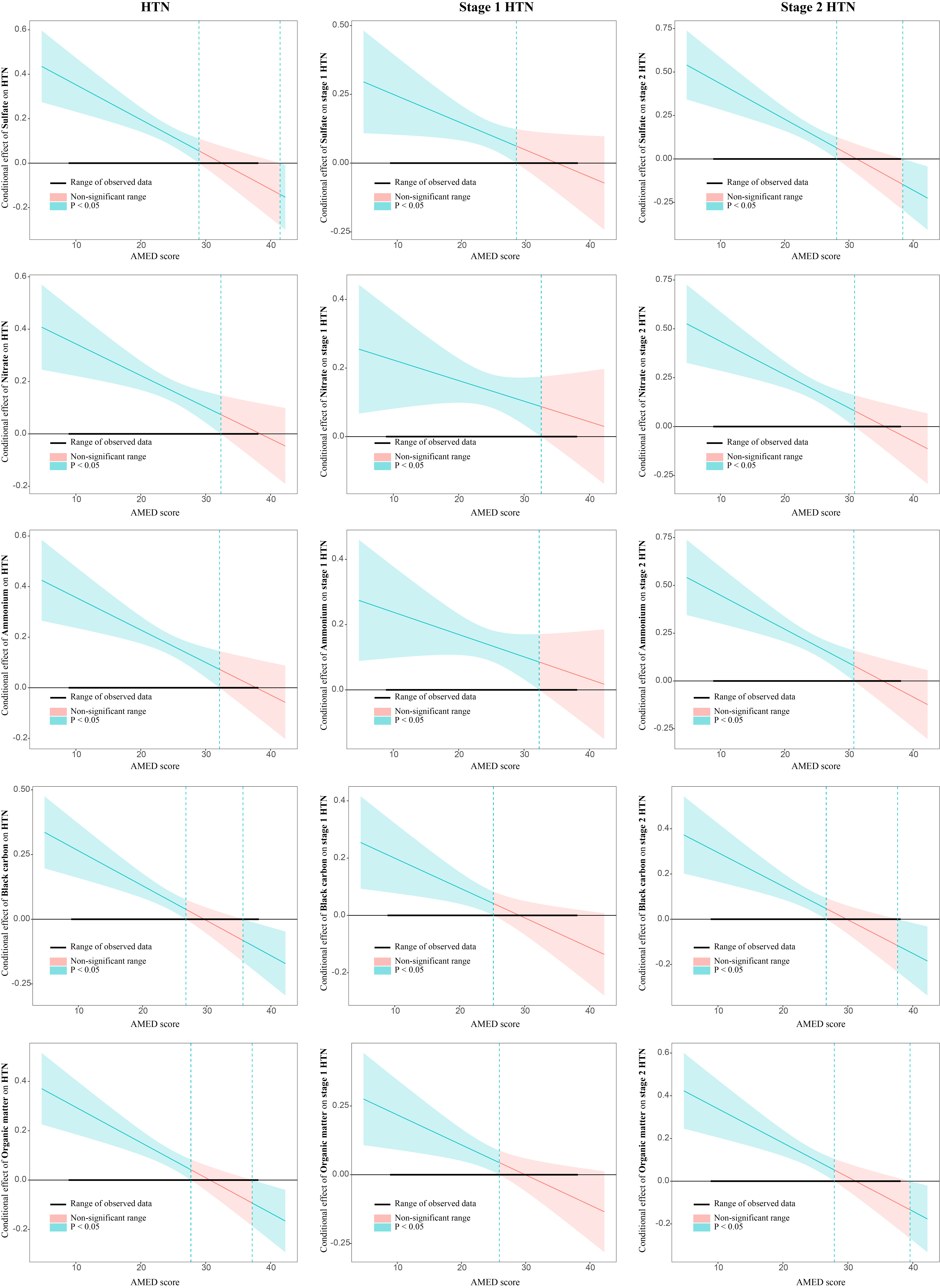


# Fig. S3 The Johnson-Neyman plots of the modifying effects of AMED scores on the relationship between long-term exposure to PM_2.5_ constituents and risk of HTN, stage 1 HTN, and stage 2 HTN.


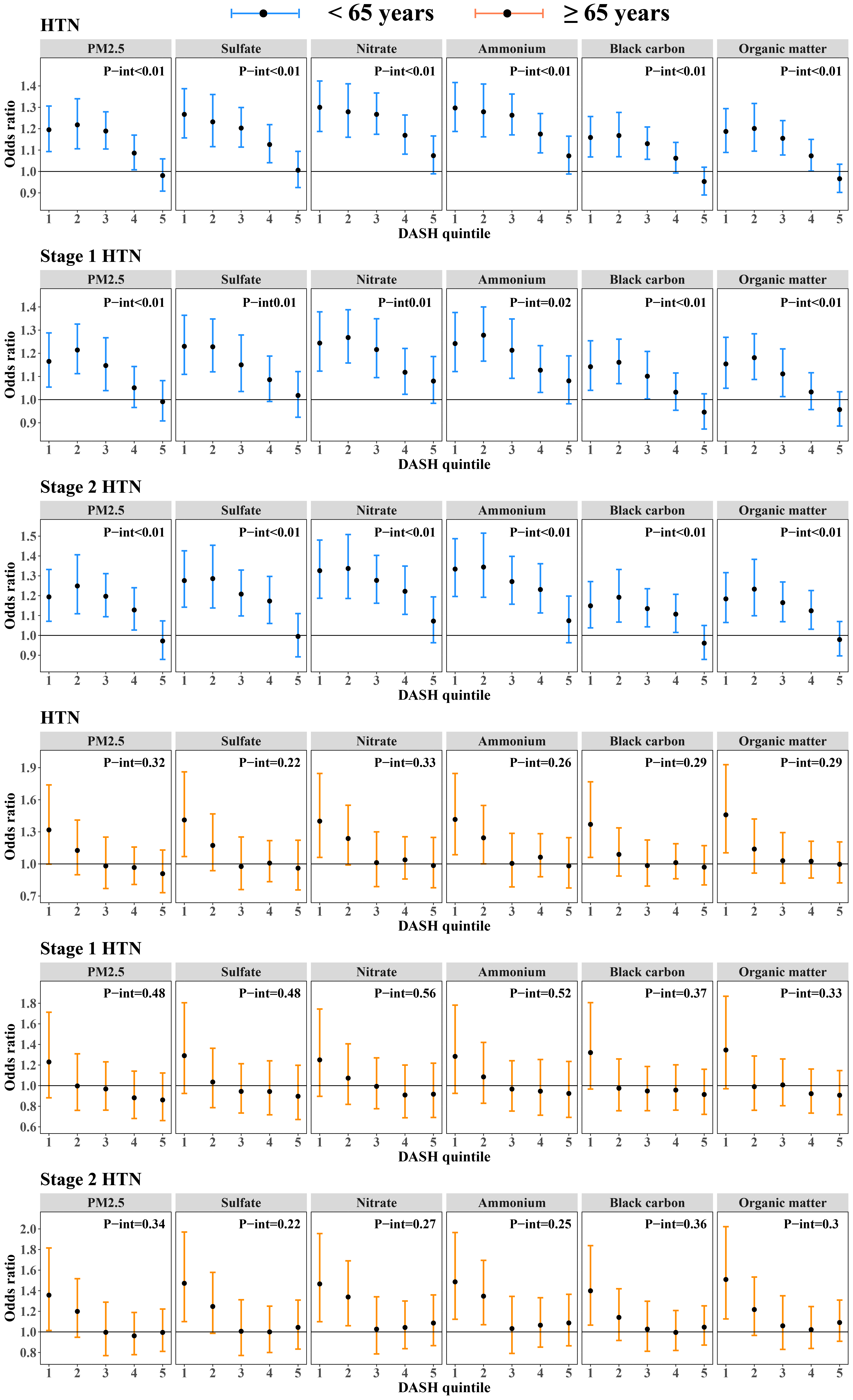


# Fig. S4 ORs and 95% CI associated with per IQR increase in PM_2.5_ and its constituents by quintiles of DASH score in different age groups.


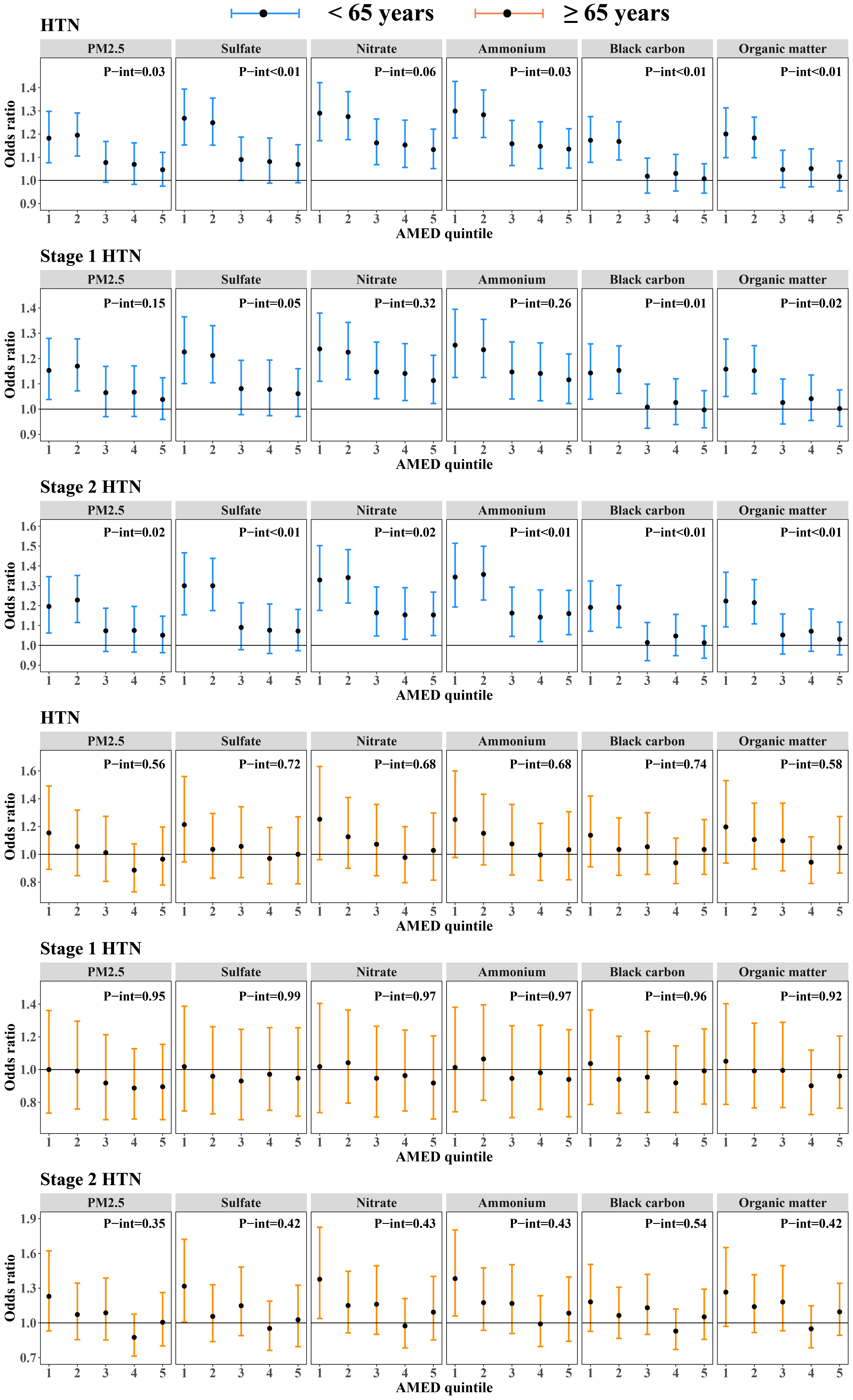


# Fig. S5 ORs and 95% CI associated with per IQR increase in PM_2.5_ and its constituents by quintiles of AMED score in different age groups.


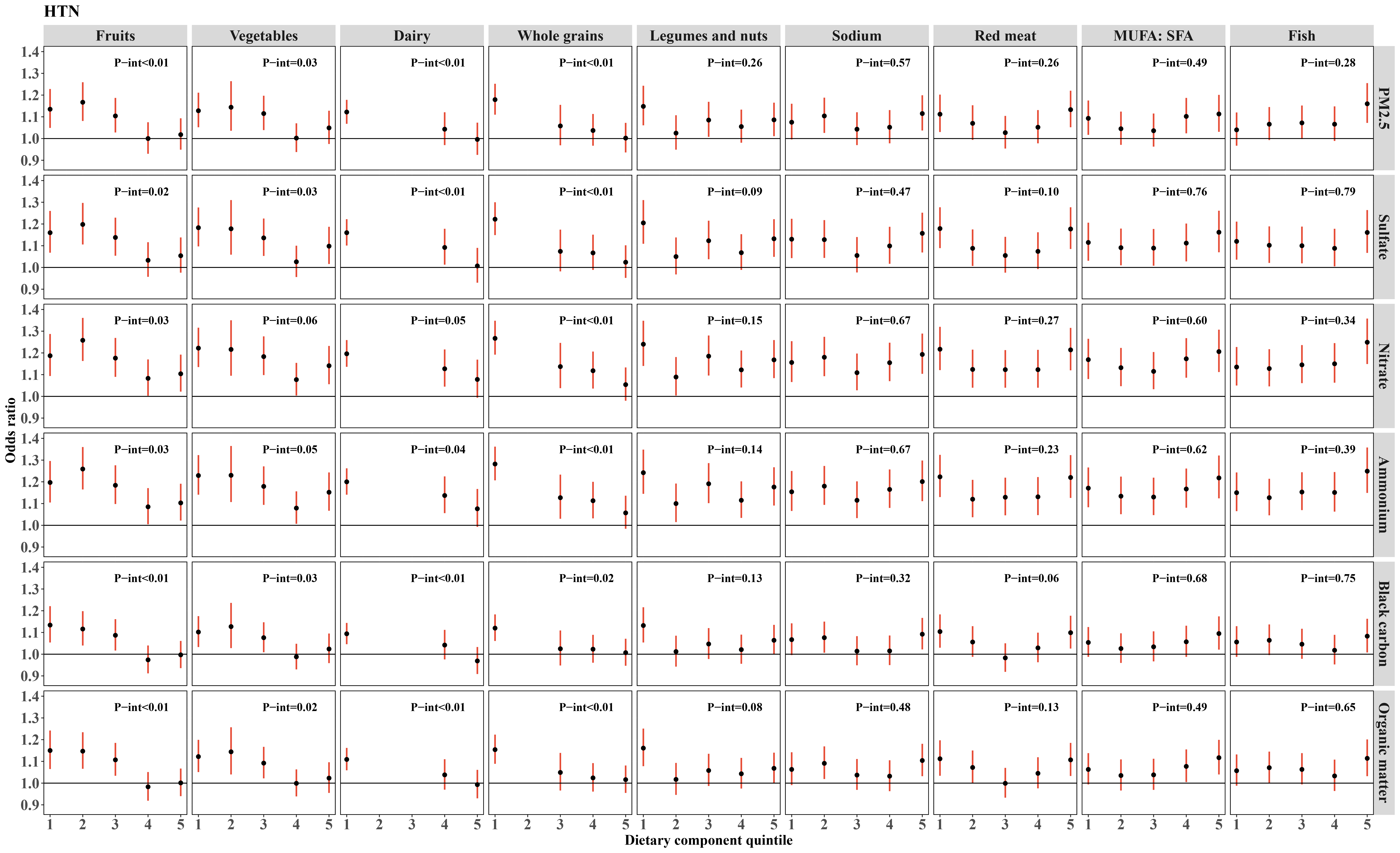


# Fig. S6 ORs and 95% CI of hypertension risk associated with per IQR increase in PM_2.5_ and its constituents by quintiles of dietary component score.


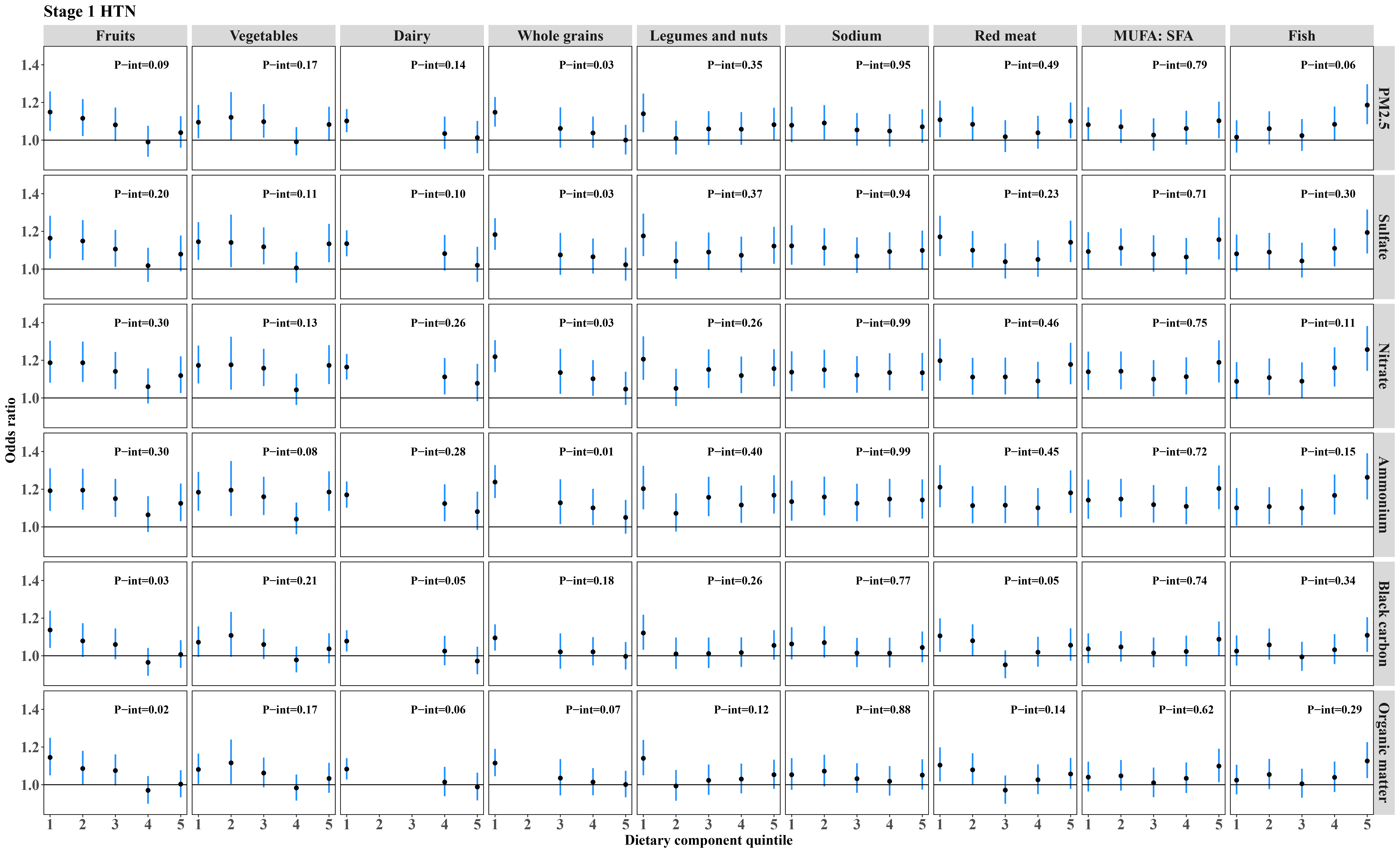


# Fig. S7 ORs and 95% CI of stage 1 hypertension risk associated with per IQR increase in PM_2.5_ and its constituents by quintiles of dietary component score.


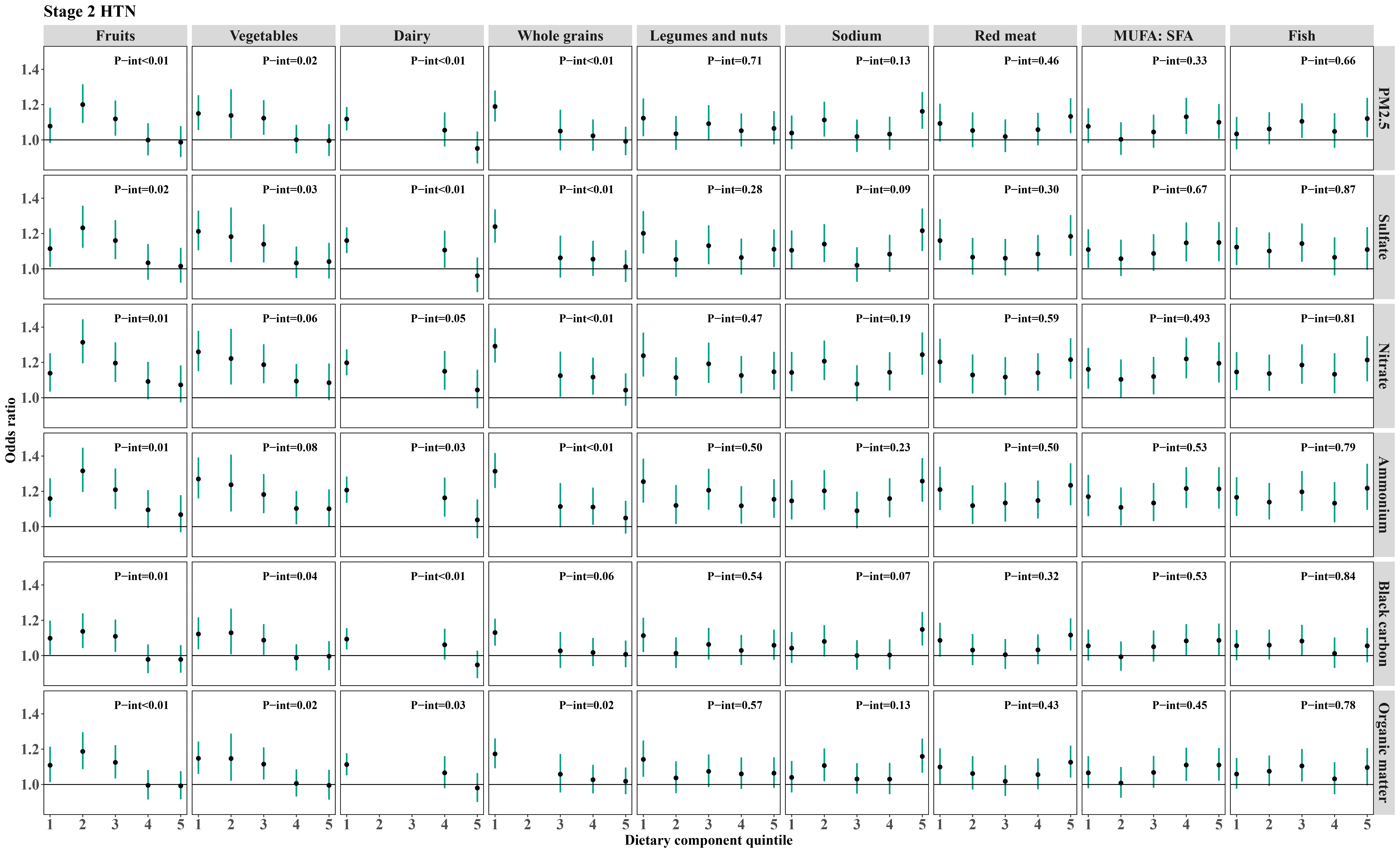


# Fig. S8 ORs and 95% CI of stage 2 hypertension risk associated with per IQR increase in PM_2.5_ and its constituents by quintiles of dietary component score.


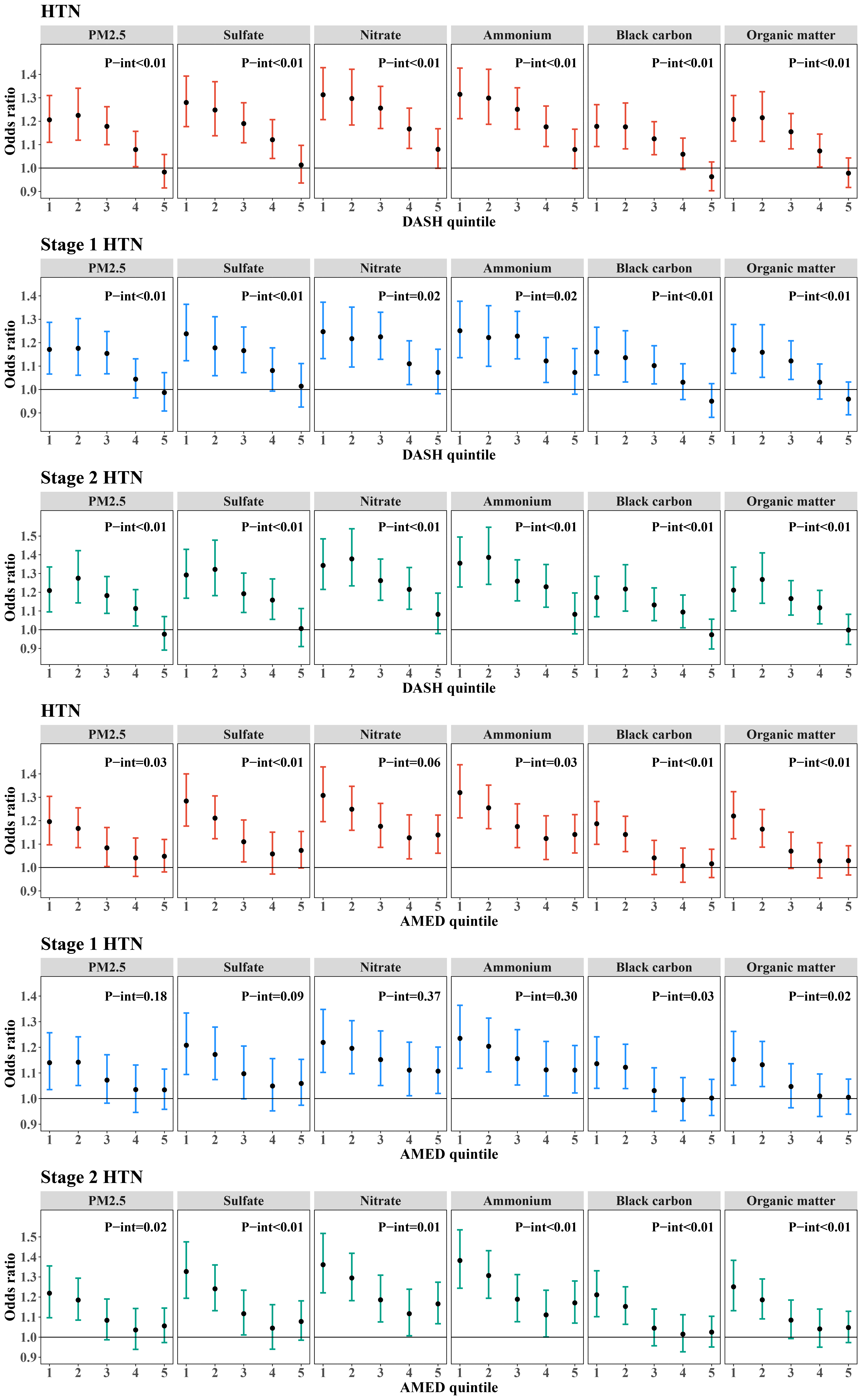


# Fig. S9 ORs and 95% CI associated with PM_2.5_ and its constituents by quintiles of DASH and AMED scores, and the model was further adjusted for preexisting (diabetes, hyperlipidemia, and cardiovascular disease).


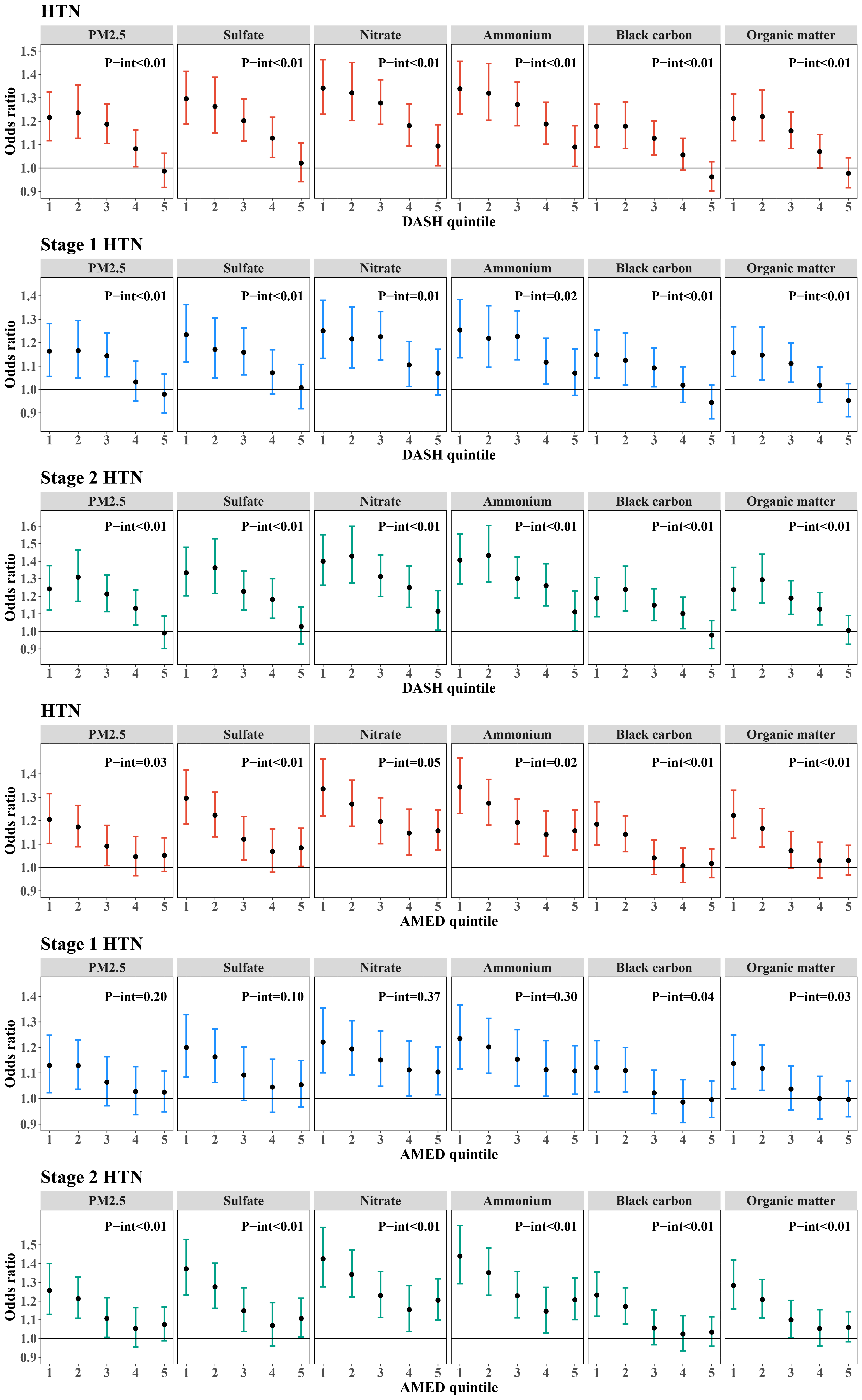


# Fig. S10 ORs and 95% CI associated with PM_2.5_ and its constituents by quintiles of DASH and AMED scores, and the model was further adjusted for ozone concentrations.


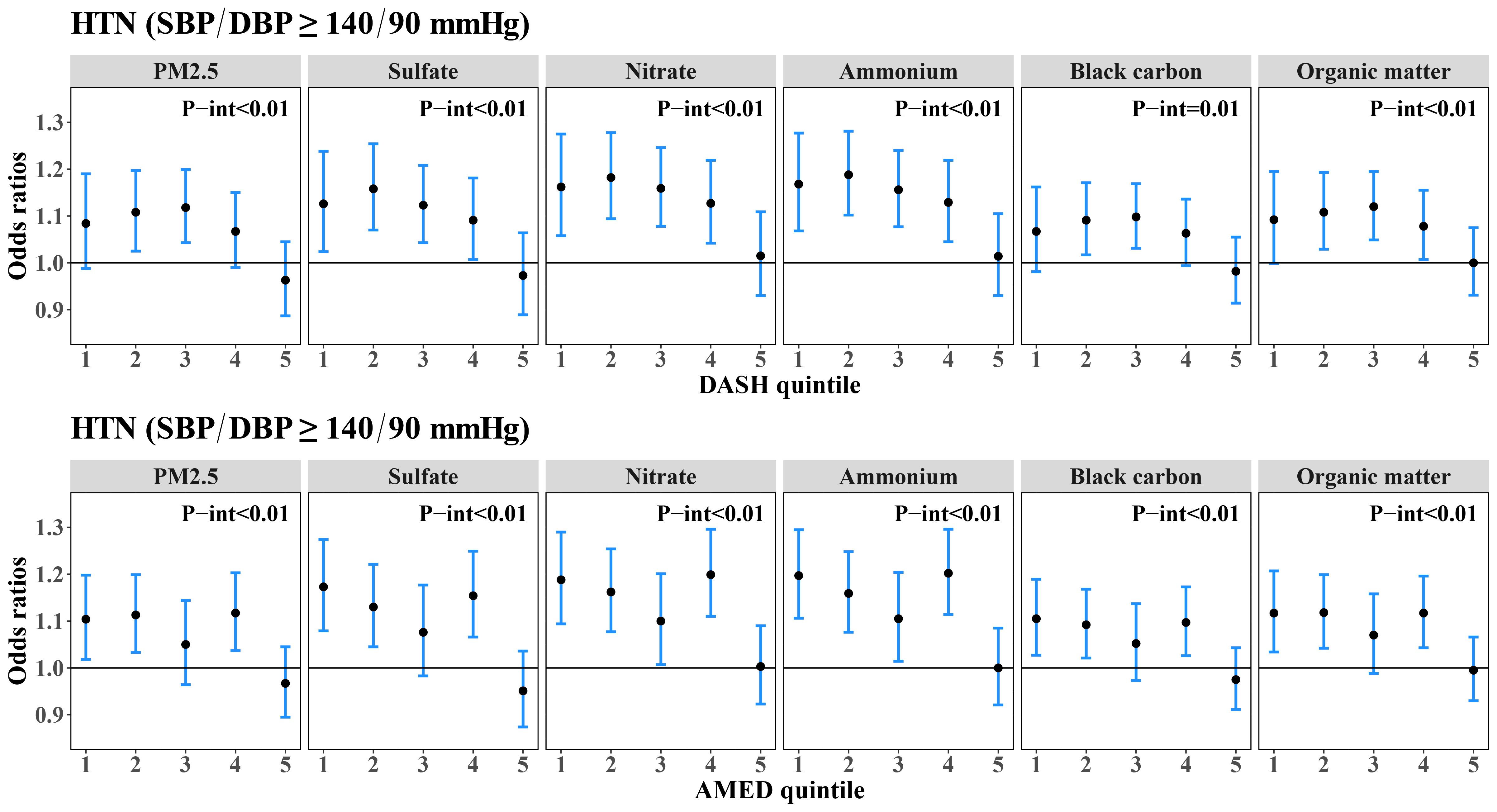


# Fig. S11 ORs and 95% CI associated with PM_2.5_ and its constituents by quintiles of DASH and AMED score after including the participants diagnosed with hypertension into analyses.
